# Association between dichotomized VASARI feature and overall survival in glioblastoma patients: a single-institution propensity score matching analysis

**DOI:** 10.1186/s40644-024-00754-z

**Published:** 2024-08-18

**Authors:** Yu Han, Yu-yao Wang, Yang Yang, Shu-qi Qiao, Zhi-cheng Liu, Guang-bin Cui, Lin-feng Yan

**Affiliations:** 1 Department of Radiology & Functional and Molecular Imaging Key Lab of Shaanxi Province, Tangdu Hospital, The Air Force Medical University, 569 Xinsi Road, Xi’an, 710038 Shaanxi Province China; 2 Department of Radiology, The 987th Hospital of Joint Logistic Support Force, People’s Liberation Army, 45# Dongfeng Road, Jintai District, Baoji, 721004 Shaanxi Province China

**Keywords:** Overall survival, Glioblastoma, Magnetic resonance imaging, Visually accessible rembrandt images

## Abstract

**Objectives:**

This study aimed to investigate the intra- and inter-observer consistency of the Visually Accessible Rembrandt Images (VASARI) feature set before and after dichotomization, and the association between dichotomous VASARI features and the overall survival (OS) in glioblastoma (GBM) patients.

**Methods:**

This retrospective study included 351 patients with pathologically confirmed IDH1 wild-type GBM between January 2016 and June 2022. Firstly, VASARI features were assessed by four radiologists with varying levels of experience before and after dichotomization. Cohen’s kappa coefficient (κ) was calculated to measure the intra- and inter-observer consistency. Then, after adjustment for confounders using propensity score matching, Kaplan-Meier curves were used to compare OS differences for each dichotomous VASARI feature. Next, patients were randomly stratified into a training set (*n* = 211) and a test set (*n* = 140) in a 3:2 ratio. Based on the training set, Cox proportional hazards regression analysis was adopted to develop combined and clinical models to predict OS, and the performance of the models was evaluated with the test set.

**Results:**

Eleven VASARI features with κ value of 0.61–0.8 demonstrated almost perfect agreement after dichotomization, with the range of κ values across all readers being 0.874–1.000. Seven VASARI features were correlated with GBM patient OS. For OS prediction, the combined model outperformed the clinical model in both training set (C-index, 0.762 vs. 0.723) and test set (C-index, 0.812 vs. 0.702).

**Conclusion:**

The dichotomous VASARI features exhibited excellent inter- and intra-observer consistency. The combined model outperformed the clinical model for OS prediction.

**Supplementary Information:**

The online version contains supplementary material available at 10.1186/s40644-024-00754-z.

## Introduction

Glioblastoma (GBM) is the most common primary malignant brain tumor [1]. The current standard of care for GBM is surgery followed by concurrent chemoradiation and adjuvant temozolomide [2], resulting in a median overall survival (OS) of 8 months [3]. Despite the poor prognosis, 30% and 10% of GBM patients achieve an OS of more than 2 and 5 years, respectively [4]. Aggressive therapy is required for this subset of patients. Therefore, accurate prognostic assessment is crucial for guiding clinical decision-making.

Several clinical factors have been identified as prognostic indicators, including age, gender, pre-operative Karnofsky performance status (KPS) score, extent of resection and treatment plan [5–7]. Although molecular biomarkers such as isocitrate dehydrogenase (IDH) mutation and O^6^-methylguanine DNA methyltransferase (MGMT) promoter methylation status, aid in the precise stratification of GBM survival [8], current methods for obtaining such information are always invasive, costly, and vulnerable to sampling errors resulting from GBM’s high heterogeneity. Therefore, noninvasive prognostic stratification tools are urgently needed.

Magnetic resonance imaging (MRI), as a noninvasive imaging modality, plays an important role in the diagnosis and prognosis assessment of GBM patients. Radiomics, as an emerging field of research, focuses on developing novel prognostic biomarkers through data-driven analysis of radiologic images [9]. However, growing evidence has confirmed that many factors affect the quality of the radiomics models, including image acquisition and normalization, segmentation, feature extraction, and computational statistics [10]. Furthermore, understanding the underlying biological rationale behind radiomics features remains challenging [11]. While radiomics has primarily academic application, its use in real-world clinical settings is currently limited.

Visually Accessible Rembrandt Images (VASARI) [12], developed for accurate and reproducible glioma assessment based on conventional MRI, can improve clinical management through easier communication of results between radiologists and clinicians. In comparison with functional MRI and radiomics, evaluating VASARI features is simple, requiring no tumor segmentation or complicated post-processing, and its results are highly interpretable, making it an invaluable tool. Previous studies have reported that VASARI features were capable of predicting the OS of GBM patients [13–16]. Although these findings are promising, there is still room for improvement. Firstly, some of the terms used are not intuitive, such as the proportions of enhancing tissue, non-enhancing tissue, necrosis, and edema, which are categorized in six groups: < 5%, 6–33%, 34–67%, 68–95%, < 95%, and 100%. This can lead to low inter-reader agreement and poor reproducibility in the daily reporting system [17]. Additionally, no comparative studies were performed with balancing known variables such as age, gender, extent of resection, KPS score, and MGMT methylation status. Moreover, in the context of 5th edition of the WHO central nervous system (CNS) tumor classification, GBM has been redefined, excluding the IDH mutation subtype [18]. Therefore, it is necessary to re-evaluate the prognostic value of VASARI features for GBM patients.

In this study, we restricted the study population to IDH1 wild-type GBM. Our aims were threefold: (i) to dichotomize VASARI feature to achieve better repeatability and stability; (ii) to investigate the relationship between dichotomous VASARI feature and the OS using propensity score matching (PSM) to adjust for confounding factors (age, gender, KPS score, extent of resection, therapy, and MGMT_status); and (iii) to develop and validate a model for predicting OS of GBM patients by integrating clinicopathologic variables and VASARI features.

## Materials and methods

### Patients

This retrospective study was approved by the Ethics Committee of Tangdu Hospital (TDLL-20151013) and written informed consent was waived. From January 2016 to June 2022, a total of 464 patients with pathologically confirmed GBM (according to the WHO 2016 classification of CNS tumors) were consecutively collected.

**Fig. 1:**
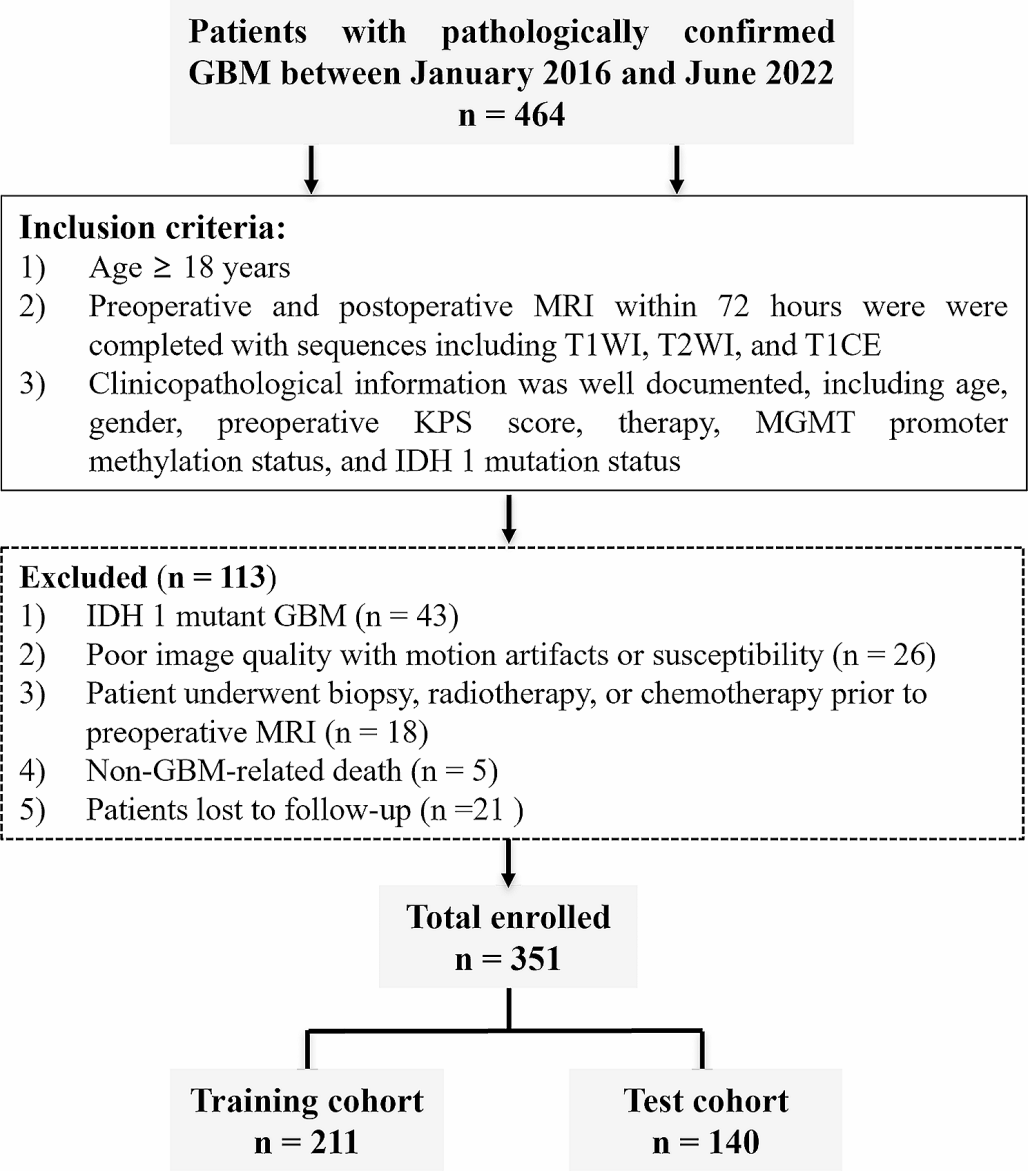
Flow diagram for patient selection

Inclusion criteria were: (i) age ≥ 18 years; (ii) preoperative and postoperative MRI were completed within 72 h, including sequences consisting of T1-weighted imaging (T1WI), T2-weighted imaging (T2WI), fluid attenuated inversion recovery (FLAIR), and contrast-enhanced T1WI (T1CE); (iii) clinicopathological information was well documented, including age, gender, preoperative KPS score, therapy, MGMT promoter methylation status, and IDH 1 mutation status. The exclusion criteria were: (i) IDH1 mutant GBM; (ii) the image quality was unsatisfactory due to susceptibility or motion artifacts; (iii) patient underwent biopsy, radiotherapy, or chemotherapy prior to preoperative MRI; (iv) deaths not related to GBM, such as acute cardiovascular and cerebrovascular events or COVID-19; (v) patients lost to follow-up immediately after discharge from the hospital following operation. Ultimately, 351 patients were enrolled. Figure 1 depicts the patient selection process.

### Study design

**Fig. 2:**
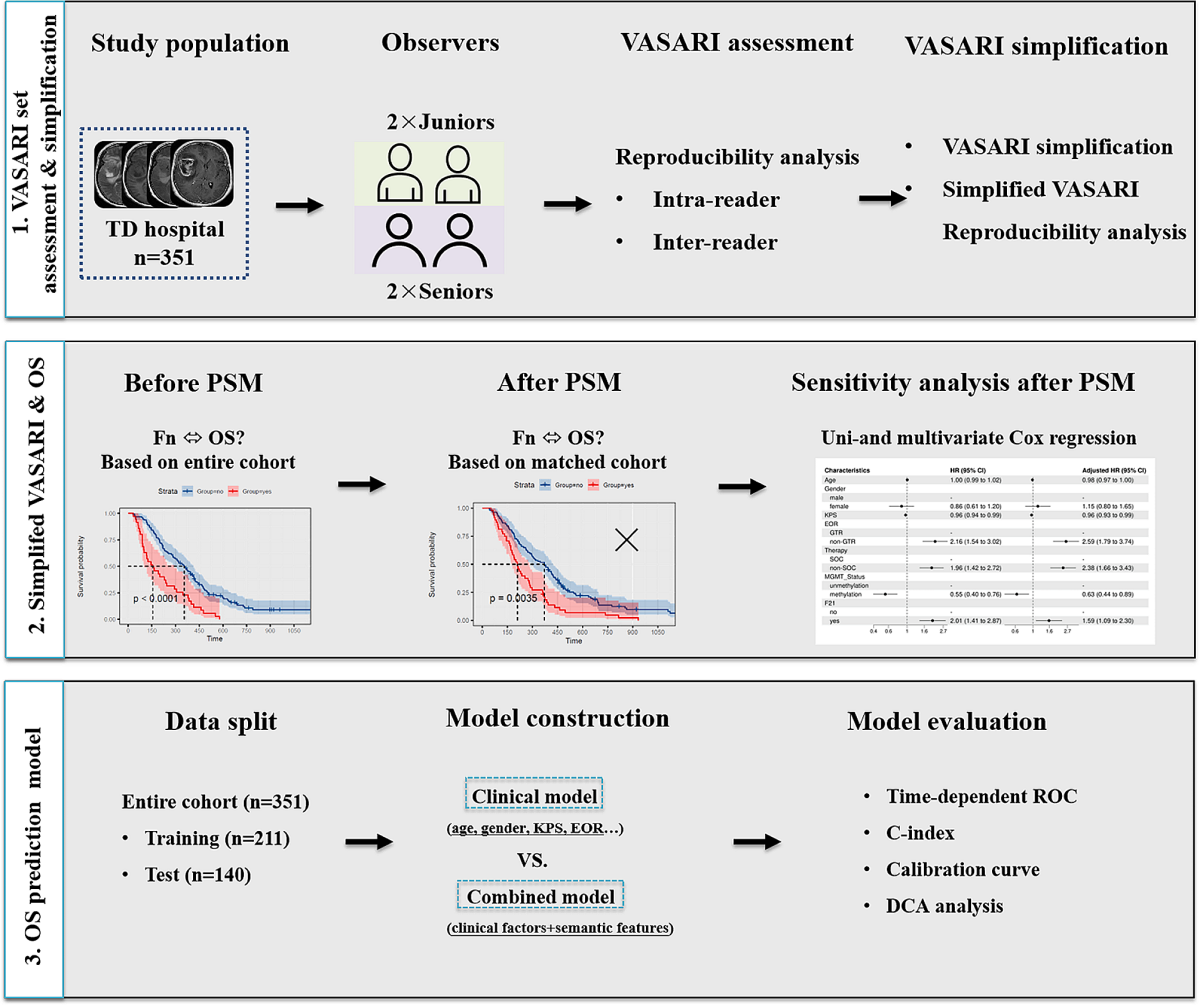
Study flow chart

The study pipeline is illustrated in Fig. 2. Initially, the consistency of VASARI feature extraction was evaluated by four radiologists with varying levels of experience. Next, 21 multi-categorical features were simplified to bi-categories. Features with κ value < 0.8 were re-evaluated for consistency. Subsequently, the relationship between dichotomous VASARI features and OS was assessed using the Kaplan-Meier survival curves on the entire cohort. For features with log-rank test *P* value < 0.05, PSM was used to control for confounders such as age, gender, KPS score, therapy, extent of resection, and MGMT promoter methylation status. Following PSM, the relationship between VASARI features and OS was re-assessed, and sensitivity analyses were conducted to evaluate the robustness of the findings. Finally, a clinical model and a combined model were constructed based on clinical, pathological, and VASARI features to predict the OS of GBM patients.

### Outcome and follow-up

The primary outcome of this study was OS, defined as days from the initial surgery to either the death or last outpatient follow-up visit. Patient follow-up data is regularly collected by specialists Y.L.Z and D.J.H through hospital chart reviews and telephone interviews. The last follow-up of patients ended on August 1, 2023.

### Definition of study variables

Baseline clinical information included age at diagnosis (treated as a continuous variable), gender (male or female), and preoperative KPS score (treated as a continuous variable). Therapy is categorized as either standard of care (SOC) or non-SOC. The standard of care encompasses a comprehensive treatment plan that includes maximal surgical resection followed by the Stupp protocol [2]. The neurosurgeon (L.W) and radiologist (L.F.Y) collaborated to determine the extent of resection (EOR), which was measured by comparing changes in tumor volume on MRI before and within 72 h after surgery. Grossly total resection (GTR) was defined as the removal of ≥ 90% of the tumor volume, while removal of < 90% was classified as non-GTR [19]. Two neuropathologists (L.L.F. and J.M.), with 10 and 8 years of experience in gliomas diagnosis, respectively, were blinded to patient information and re-evaluated the pathological data. Of these, molecular phenotypes including IDH1/2 mutation status (wild-type or mutant) and MGMT promoter methylation status (methylation or unmethylation), was evaluated using polymerase chain reaction and direct sequencing, respectively.

### MRI acquisitions

MRI studies were conducted at our institution using either a 3.0 T or 1.5 T unit from different scanners, with various parameters to reflect real-life inter-center heterogeneity. The brain tumor imaging protocol included T1WI, T2WI, FLAIR, and T1CE. The detailed acquisition parameters are summarized in the **Supplementary Table ****S1**.

### VASARI feature set: consistency analyses, dichotomization and reanalyses

The image analysis pipeline is shown in step 1 of Fig. 2. Before starting image analysis, all pre- and post-operative MRI images were anonymized. VASARI feature extraction was independently performed by four radiologists, consisting of two senior radiologists (Z.C.L and Y.H, with 10, 12 years of experience in brain tumor diagnosis, respectively) and two junior radiologists (J.Z and Y.Y.W, with 4, 6 years of experience in brain tumor diagnosis, respectively). Prior to feature extraction, each radiologist reviewed the VASARI criteria (**Supplementary Table S2**) and then participated in a 1-hour teaching session where the senior neuroradiologist (G.B.C, with 20 years of experience in brain tumor diagnosis) provided a review of VASARI feature scoring system for gliomas (https://wiki.nci.nih.gov/display/CIP/VASARI) and presented several example cases. It should be noted that feature F17 (diffusion) was not extracted in the present study cohort due to the absence of diffusion-weighted imaging sequences. After a 2-month washout period, reassessment was conducted by senior members of the junior and senior groups.

To improve the consistency of VASARI feature and increase its clinical utility, we simplified the 21 multi-categorical VASARI features into dichotomous ones, with criteria detailed in Table 1. Dichotomized VASARI feature is denoted as FnM. For those features with κ value < 0.8 before simplification, consistency analyses were conducted twice, with 3-month washout period, to evaluate the impact of the simplification.

**Table 1:** Dichotomized VASARI feature set definition and classification criteria

Feature	Name	Description	Options
**F1M**	Tumor Location	Location of lesion geographic epicenter (not all areas of involvement)	1 = Frontal 2 = Temporal/ Others
**F2M**	Side of Tumor Epicenter	Side of lesion epicenter	1 = Unilateral 2 = Center/Bilateral
**F3M**	Eloquent Brain	Does the geographic center or the enhancing component involve eloquent cortex (motor, language, vision) or key underlying white matter?	1 = None 2 = Yes
**F4M**	Enhancement Quality	[None, Mild, Moderate, Marked]. Qualitative degree of contrast enhancement is defined as having all or portions of the tumor that demonstrate significantly higher signal on the postcontrast T1W images compared to precontrast T1W images.	1 = None/Mild/Minimal 2 = Marked/Avid
**F5M**	Proportion Enhancing	What proportion of the entire tumor is enhancing?	1 = < 50% 2 = ≥ 50%
**F6M**	Proportion nCET	What proportion of the entire tumor is non-enhancing?	1 = < 50% 2 = ≥ 50%
**F7M**	Proportion Necrosis	What proportion of the entire tumor is necrosis?	1 = < 50% 2 = ≥ 50%
**F9M**	Multifocal or Multicentric	Multifocal is defined as having at least one region of tumor, either enhancing or nonenhancing, which is not contiguous with the dominant lesion and is outside the region of signal abnormality (edema) surrounding the dominant mass. This can be defined as those resulting from dissemination or growth by an established route, spread via commissural or other pathways, or via CSF channels or local metastases, whereas Multicentric are widely separated lesions in different lobes or different hemispheres that cannot be attributed to one of the previously mentioned pathways. Gliomatosis refers to generalized neoplastic transformation of the white matter of most of a hemisphere.	1 = None 2 = Multifocal/ Multicentric
**F10M**	T1/FLAIR RATIO	Tumor feature summary. [Mixed, expansive or infiltrative]. Expansive = size of pre-contrast T1abnormality (exclusive of signal intensity) approximates size of FLAIR abnormality. Mixed = Size of T1 abnormality moderately less than FLAIR envelope; Infiltrative = Size of pre-contrast T1 abnormality much smaller than size of FLAIR abnormality. (Use T2 if FLAIR is not provided)	1 = Expansive/Mixed 2 = Infiltrative
**F11M**	Thickness of enhancing margin	The scoring is not applicable if there is no contrast enhancement. If most of the enhancing rim Is thin, regular, and has homogenous enhancement the grade is thin. If most of the rim demonstrates nodular and/or thick enhancement, the grade is thick. If there is only solid enhancement and no rim, the grade is None.	1 = None/Thin 2 = Thick/solid
**F12M**	Definition of the enhancing margin	if most of the outside margin of the enhancement is well defined or poorly defined.	1 = Well-defined 2 = Poorly-defined
**F13M**	nCET	Whether the nCET exists. Nonenhancing tumor is defined as regions of T2W hyperintensity (less than the intensity of cerebrospinal fluid, with corresponding T1W hypointensity) that are associated with mass effect and architectural distortion, including blurring of the gray-white interface. (Assuming that the the entire abnormality may be comprised of: (1) an enhancing component, (2) a non-enhancing component, (3) a necrotic component and (4) a edema component.).	1 = No 2 = Yes
**F14M**	Proportion of Edema	What proportion of the entire abnormality is vasogenic edema?	1 = < 50% 2 = ≥ 50%
**F15M**	Edema Crosses Midline	Edema spans white matter commissures extending into contralateral hemisphere (exclusive of herniated ipsilateral tissue).	1 = No 2 = Yes
**F22M**	nCET tumor Crosses Midline	nCET crosses into contralateral hemisphere through white matter commissures (exclusive of herniated ipsilateral tissue).	1 = No 2 = Yes
**F23M**	Enhancing tumor Crosses Midline	Enhancing tissue crosses into contralateral hemisphere through white matter commisures (exclusive of herniated ipsilateral tissue).	1 = No 2 = Yes
**F26M**	Total resection of enhancing tumor	Whether the enhancing tumor was totally removed.	1 = No 2 = Yes
**F27M**	Total resection of nCET	Whether the nCET was totally removed.	1 = No 2 = Yes
**F28M**	Extent resection of edema	Estimate the proportion of edema removed.	1 = < 50% 2 = ≥ 50%
**F29M &F30M**	Lesion Size	Largest perpendicular (x-y) cross-sectional diameter of T2 signal abnormality (longest dimension X perpendicular dimension) measured on a single axial image only.	1 = < 4.25 cm 2 = ≥ 4.25 cm

Note: Dichotomized VASARI feature is denoted as FnM

### Statistical analysis

All statistical analyses were conducted by Y.Y. and J.H.L using R software (Version 3.5.3). Two-tailed *P* < 0.05 indicated a significant difference.

### Comparison of baseline characteristics

Continuous variables are presented as “mean ± standard deviation (SD)” or “median (interquartile range)” and were compared between groups using either the student *t* test or Mann-Whitney U test, depending on the normality of the data. Categorical variables are presented as “number (percentage)” and were compared between groups using chi-square or Fisher’s exact test.

### Consistency analysis for VASARI feature set

The consistency of VASARI features before and after dichotomization was assessed using Cohen Kappa analysis, in which the ordered VASARI features were analyzed using linearly weighted kappa coefficient [20].

### Propensity score matching

We employed PSM to mitigate the confounding effects of known predictors for GBM survival, thus enhancing the relevance of the results to real-world clinical practice. Propensity scores were calculated using logistic regression with the following covariates: age, gender, KPS score, EOR, therapy, and MGMT_status. Patients were then selected by 1:1 matching without replacement using the nearest-neighbor method based on their propensity scores. A caliper width of 0.1 standardized differences was used for matching [21, 22]. To boost the total sample size, other PSM ratios were considered, such as 1:2, 1:3, and 1:4 [21].

### Association of VASARI features with overall survival

As shown in step 2 of Fig. 2, Kaplan–Meier analysis with the log-rank test was used to compare the OS of both the before- and after-PSM cohorts. After PSM, we further conducted sensitivity analyses using univariable and multivariable Cox regression to determine whether the association between OS and VASARI feature remained stable. The following variables were adjusted in multivariable Cox regression including: age, gender, KPS score, EOR, therapy, and MGMT_status.

### Overall survival prediction model construction and performance evaluation

As shown in step 3 of Fig. 2, all patients were divided into a training set (*n* = 211) and test set (*n* = 140) at a ratio of 3:2 using random stratified sampling to ensure balanced patient characteristics. Multivariate Cox proportional hazards analyses with backward stepwise elimination were performed to develop OS prediction models using the training set. The performance of models was quantified with Harrell’s concordance probability index (C-index). Areas under the time-dependent ROC curves (AUC) for the 1-year, 2-year, and 3-year follow-ups were calculated and compared between models. Calibration curves and decision curves were plotted to assess the usefulness of the predictive models. The model-averaged importance of the variables was assessed by the best subsets approach [23].

## Results

### Patient characteristics

A total of 351 patients were enrolled. There were 228 men and 123 women, with a median age of 58 years (range: 51 − 65 years). Table 2 summarizes the patient characteristics of the entire cohort, as well as the training and test cohorts. The median OS of the entire cohort, training cohort and test cohort were 348 days, 320 days, and 363 days, respectively. No significant differences in age, gender, KPS score, therapy, EOR, or MGMT_status was observed between the training and test cohorts (all *P* > 0.05).

**Table 2:** Baseline characteristics of the patients

Characteristics	levels	Entire cohort (*n* = 351)	Training (*n* = 211)	Test (*n* = 140)	*P* ^†^
**OS**	Median (IQR)	348.00 (194.00-500.50)	320.00 (198.50–480.00)	363.00 (190.50–534.00)	0.408
**Age**	Median (IQR)	58.00 (51.00–65.00)	58.00 (50.00–65.00)	59.00 (52.00–66.00)	0.376
**Gender**	male	228 (64.96%)	143 (67.8%)	85 (60.7%)	0.214
	female	123 (35.04%)	68 (32.2%)	55 (39.3%)	
**KPS score**	Median (IQR)	90.00 (80.00–90.00)	90.00 (80.00–90.00)	90.00 (80.00–90.00)	0.468
**Therapy**	SOC	204 (58.12%)	120 (56.9%)	84 (60%)	0.638
	non-SOC	147 (41.88%)	91 (43.1%)	56 (40%)	
**EOR**	GTR	231 (65.81%)	134 (63.5%)	97 (69.3%)	0.316
	non-GTR	120 (34.19%)	77 (36.5%)	43 (30.7%)	
**MGMT_Status**	methylation	192 (54.70%)	119 (56.4%)	73 (52.1%)	0.500
	unmethylation	159 (45.30%)	92 (43.6%)	67 (47.9%)	

OS, overall survival; IQR, interquartile range; KPS, Karnofsky performance status; EOR, extent of resection; GTR, gross total resection; SOC, standard of care; MGMT, O^6^-methylguanine DNA methyltransferase

^†^*P* values were calculated to compare baseline characteristics between the training and test sets

### Consistency analyses for VASARI feature set before and after dichotomization

Table 3 presents the results of consistency analyses conducted on the VASARI feature set before and after dichotomization. The results indicate that the senior group outperformed the junior group in the consistency analyses. Before dichotomization, a total of 11 VASARI features with κ value < 0.8 were identified in both the junior and senior groups: F3 (eloquent brain), F5 (proportion enhancing), F6 (proportion nCET), F7 (proportion necrosis), F9 (multifocal or multicentric), F13 (definition of the non-enhancing margin), F14 (proportion of edema), F22 (nCET tumor crosses midline), F26 (extent of resection of enhancing tumor), F27 (extent resection of nCET) and F28 (extent resection of vasogenic edema). After dichotomization, the consistency of these aforementioned features improved, with κ value ranging from 0.874 to 1.000.

**Table 3:** Inter-observer and intra-observer consistency analyses of VASARI feature sets before and after dichotomization for junior and senior groups

VASARI	κ value (Senior radiologists)									κ value (Junior radiologists)					
	Inter-observer agreement				Intra-observer agreement				Inter-observer agreement					Intra-observer agreement	
	Before DM	After DM			Before DM	After DM			Before DM		After DM			Before DM	After DM
F1	0.887 (0.799–0.976)				0.960 (0.904-1.000)				0.824 (0.717–0.930)					0.905 (0.822–0.987)	
F2	0.983 (0.948-1.000)				1.000 (1.000–1.000)				0.966 (0.918-1.000)					1.000 (1.000–1.000)	
F3	0.794 (0.691–0.898)	0.992 (0.976-1.000)			0.873 (0.787–0.959)	1.000 (1.000–1.000)			0.726 (0.614–0.838)		0.961 (0.927–0.995)			0.794 (0.691–0.898)	0.978 (0.948-1.000)
F4	0.819 (0.720–0.918)				0.918 (0.846–0.989)				0.807 (0.705–0.908)					0.873 (0.787–0.959)	
F5	0.819 (0.720–0.918)	0.976 (0.949-1.000)			0.873 (0.787–0.959)	1.000 (1.000–1.000)			0.685 (0.570–0.801)		0.961 (0.927–0.995)			0.759 (0.650–0.867)	0.991(0.972-1.000)
F6	0.716 (0.602–0.829)	0.983 (0.960-1.000)			0.819 (0.720–0.918)	0.991 (0.973-1.000)			0.614 (0.496–0.732)		0.935 (0.890–0.979)			0.770 (0.663–0.877)	0.972 (0.942-1.000)
F7	0.748 (0.638–0.858)	0.983 (0.960-1.000)			0.799 (0.708–0.890)	1.000 (1.000–1.000)			0.622 (0.505–0.740)		0.959 (0.923–0.995)			0.705 (0.591–0.819)	1.000 (1.000–1.000)
F8	0.920 (0.850–0.989)				0.983 (0.950-1.000)				0.846 (0.752–0.939)					0.902 (0.825–0.979)	
F9	0.817 (0.737–0.897)	1.000 (1.000–1.000)			0.913 (0.854–0.971)	1.000 (1.000–1.000)			0.774 (0.691–0.857)		1.000 (1.000–1.000)			0.821 (0.736–0.907)	1.000 (1.000–1.000)
F10	0.826 (0.747–0.906)				0.880 (0.813–0.946)				0.801 (0.706–0.896)					0.836 (0.745–0.928)	
F11	0.858 (0.798–0.918)				0.901 (0.846–0.956)				0.814 (0.733–0.895)					0.874 (0.802–0.945)	
F12	0.877 (0.819–0.936)				0.863 (0.775–0.950)				0.809 (0.711–0.906)					0.823 (0.727–0.920)	
F13	0.764 (0.658–0.870)	0.965 (0.931–0.999)			0.811 (0.712–0.910)	0.983 (0.960-1.000)			0.612 (0.496–0.728)		0.948 (0.907–0.989)			0.721 (0.610–0.832)	0.959 (0.923–0.995)
F14	0.781 (0.683–0.878)	0.951 (0.912–0.990)			0.801 (0.704–0.898)	0.968 (0.936–0.999)			0.682 (0.604–0.760)		0.935 (0.891–0.980)			0.752 (0.669–0.836)	0.936 (0.893–0.980)
F15	1.000 (1.000–1.000)				1.000 (1.000–1.000)				0.960 (0.904-1.000)					0.981 (0.944-1.000)	
F16	0.981 (0.944-1.000)				1.000 (1.000–1.000)				0.941 (0.874-1.000)					0.960 (0.904-1.000)	
F18	0.901 (0.844–0.959)				0.966 (0.927-1.000)				0.830 (0.758–0.901)					0.893 (0.834–0.952)	
F19	0.954 (0.913–0.994)				0.973 (0.943-1.000)				0.807 (0.734–0.880)					0.874 (0.812–0.936)	
F20	0.851 (0.782–0.920)				0.972 (0.940-1.000)				0.857 (0.791–0.923)					0.866 (0.803–0.930)	
F21	0.956 (0.917–0.994)				1.000 (1.000–1.000)				0.932 (0.886–0.979)					0.983 (0.958-1.000)	
F22	0.769 (0.690–0.848)	0.928 (0.882–0.974)			0.886 (0.831–0.941)	0.960 (0.925–0.995)			0.703 (0.614–0.793)		0.905 (0.853–0.958)			0.768 (0.685–0.852)	0.921 (0.873–0.969)
F23	0.969 (0.935-1.000)				1.000 (1.000–1.000)				0.908 (0.850–0.967)					0.959 (0.920–0.999)	
F24	0.989 (0.968-1.000)				1.000 (1.000–1.000)				0.947 (0.901–0.993)					0.978 (0.949-1.000)	
F25	0.989 (0.968-1.000)				1.000 (1.000–1.000)				0.978 (0.949-1.000)					1.000 (1.000–1.000)	
F26	0.767 (0.719–0.814)	0.960 (0.925–0.995)			0.825 (0.781–0.870)	0.984 (0.962-1.000)			0.641 (0.534–0.761)		0.911 (0.860–0.963)			0.780 (0.732–0.828)	0.923 (0.876–0.970)
F27	0.779 (0.740–0.819)	0.929 (0.883–0.975)			0.794 (0.753–0.835)	0.961 (0.927–0.995)			0.705 (0.648–0.783)		0.896 (0.841–0.951)			0.756 (0.697–0.814)	0.908 (0.858–0.959)
F28	0.722 (0.662–0.782)	0.926 (0.879–0.974)			0.779 (0.725–0.833)	0.953 (0.916–0.990)			0.684 (0.617–0.750)		0.874 (0.814–0.934)			0.736 (0.679–0.794)	0.916 (0.867–0.965)
F29	0.956 (0.936–0.976)				0.968 (0.950–0.985)				0.900 (0.857–0.942)					0.946 (0.923–0.968)	
F30	0.945 (0.924–0.967)				0.971 (0.957–0.986)				0.937 (0.910–0.963)					0.961 (0.942–0.980)	

DM, dichotomization

### Association between VASARI features and overall survival before and after PSM

As shown in Table 4, the Kaplan-Meier curve analyses indicated that 11 VASARI features were associated with OS of GBM patients in the entire cohort. Subsequently, the distribution of confounders (age, gender, KPS, EOR, therapy, and MGMT_status) for these features was balanced between groups using PSM. The baseline characteristics, love plots for standardized mean difference, and Kaplan-Meier curves before and after matching are provided in the **Supplementary files (Table S3-S24**,** Figure ****S1****-****S1****1)**. After performing PSM, we identified 7 features that were significantly associated with shorter OS. These features are as follows: F2M (center/bilateral), F12M (poorly-defined), F15M (edema crosses midline), F19 (ependymal invasion), F21 (deep white matter invasion), F22M (nCET tumor crosses midline), and F23M (enhancing tumor crosses midline). Notably, the relationship between these features and OS remained consistent even after sensitivity analyses.

**Table 4:** Association between VASARI features and overall survival before and after propensity score matching

Variables	Before PSM				After PSM				Sensitivity analysis (after PSM)			
	*N*	OS (median)	*P*		*N*	OS (median)	*P*		HR(95%CI)	*P*	Adjusted HR (95%CI)	*P*
**F2M**												
Unilateral (-ref)	131	365			97	270						
center/bilateral	38	150	**< 0.001**		38	150	**0.01**		1.77 (1.20–2.60)	**0.004**	1.96 (1.32–2.89)	**< 0.001**
**F8**												
No	303	313			131	412						
Yes	48	472	**0.01**		48	472	0.39		0.73 (0.50–1.07)	0.103	0.69 (0.47–1.01)	0.057
**F9M**												
None	286	366			142	274						
multifocal/multicentric	65	281	**< 0.001**		62	281	0.38		1.11 (0.81–1.51)	0.514	0.86 (0.63–1.19)	0.372
**F12M**												
well-defined (-ref)	115	415			113	411						
poorly-defined	236	290	**< 0.001**		113	332	**0.007**		1.48 (1.10–1.98)	**0.009**	1.46 (1.08–1.96)	**0.013**
**F15M**												
no(-ref)	316	364			93	270						
Yes	35	145	**< 0.001**		35	145	**0.042**		1.66 (1.12–2.48)	**0.012**	1.75 (1.17–2.63)	**0.007**
**F16**												
no(-ref)	227	365			109	360						
Yes	124	306	**0.017**		109	303	0.35		1.15 (0.86–1.52)	0.346	1.06 (0.79–1.41)	0.704
**F19**												
no(-ref)	151	461			130	455						
Yes	200	267	**< 0.001**		130	303	**< 0.001**		2.24 (1.70–2.95)	**< 0.001**	2.02 (1.53–2.68)	**< 0.001**
**F21**												
no(-ref)	289	373			114	372						
Yes	62	210	**< 0.001**		48	210	**0.004**		2.01 (1.41–2.87)	**< 0.001**	1.59 (1.09–2.30)	**0.015**
**F22M**												
no(-ref)	281	371			150	326						
Yes	70	220	**< 0.001**		66	231	**0.002**		1.56 (1.16–2.10)	**0.004**	0.89 (0.89–1.67)	**0.011**
**F23M**												
no(-ref)	315	365			126	353						
Yes	36	150	**< 0.001**		35	155	**< 0.001**		1.92 (1.36–2.69)	**< 0.001**	1.48 (1.01–2.16)	**0.045**
**F24**												
no(-ref)	320	365			109	236						
Yes	31	221	**< 0.001**		31	221	0.37		1.34 (0.89-2.00)	0.161	1.18 (0.76–1.82)	0.468

HR, hazards ratio; OS, overall survival; PSM, propensity score matching; CI, confidence interval

Adjusted HR was for age, gender, KPS score, extent of resection, therapy, and MGMT_Status.

Bold values represent *P* < 0.05

### Performance evaluation of overall survival prediction models

**Fig. 3:**
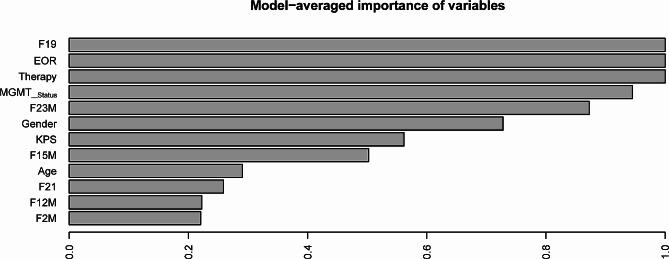
Analysis of model-averaged importance of candidate variables

The clinical and combined models were constructed using the training cohort. According to the model-averaged importance, F19, EOR, and therapy were the top-ranked variables (Fig. 3). The results of multivariate Cox proportional hazards regression analyses for both models are presented in the **Supplementary files (Table S25-S26)**.

As shown in Table 5, the combined model exhibited superior performance compared to the clinical model. In the training set, the C-index for the combined and clinical model were 0.762 (0.734–0.790), and 0.723 (0.688–0.758), respectively. In the test set, the C-index was 0.812 (0.773–0.851), and 0.702 (0.660–0.744) for the combined and clinical models, respectively.

**Table 5:** Comparison of discrimination performance between clinical and combined model in the training and test cohorts

	Training cohort (*n* = 211)					Test cohort (*n* = 140)		
		Clinical model	Combined model	*P*		Clinical model	Combined model	*P*
**1-year OS_AUC (95%CI)**		0.786 (0.724–0.848)	0.832 (0.765–0.899)	**0.039**		0.797 (0.737–0.857)	0.811 (0.739–0.883)	0.563
**2-year OS_AUC (95%CI)**		0.741 (0.584–0.897)	0.809 (0.735–0.884)	**< 0.001**		0.710 (0.566–0.853)	0.787 (0.646–0.928)	**0.046**
**3-year OS_AUC (95%CI)**		0.799 (0.719–0.879)	0.853 (0.791–0.915)	**0.021**		0.762 (0.650–0.874)	0.879 (0.783–0.975)	**< 0.001**
**C-index (95%CI)**		0.723 (0.688–0.758)	0.762 (0.734–0.790)	NA		0.702 (0.660–0.744)	0.812 (0.773–0.851)	NA

AUC, area under the receiver operating characteristics curve; CI, confidence interval; OS, overall survival; NA, not available

Clinical model: age + gender + EOR + KPS score + therapy + MGMT_status.

Combined model: age + gender + EOR + KPS score + therapy + MGMT_status + F15M + F19 + F21 + F23M.

Bold values represent *P* < 0.05

Table 5 also shows the time-dependent discrimination measures for death up to 3 years for the clinical and combined models. Based on training cohort, the combined model achieved better performance than the clinical model for predicting 1-year OS (combined model: AUC, 0.832 vs. clinical model: AUC, 0.786, *P* = 0.039), 2-year OS (combined model: AUC, 0.809 vs. clinical model: AUC, 0.741, *P* < 0.001), and 3-year OS (combined model: AUC, 0.853 vs. clinical model: AUC, 0.799, *P* = 0.021). Similarly, in the test set, the AUC values were higher for the combined model compared to the clinical model, and the difference was statistically significant, except for 1-year OS.

**Fig. 4:**
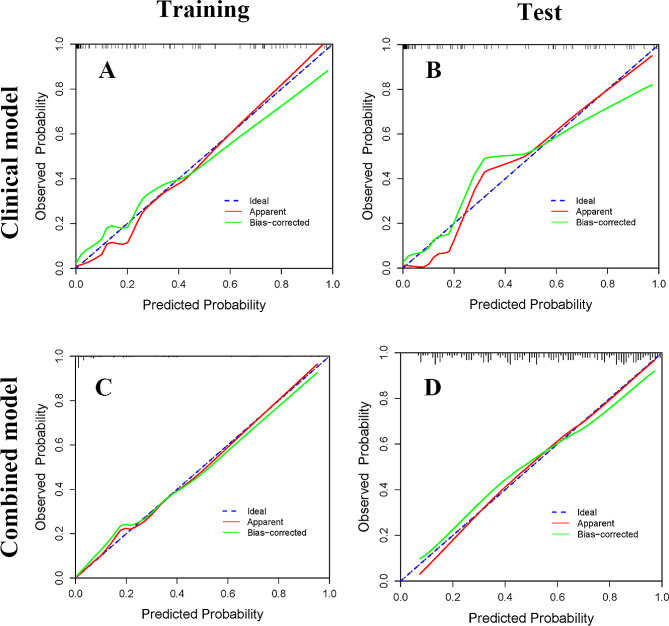
Calibration curves for the clinical model (**A**, training set; **B**, test set) and combined model (**C**, training set; **D**), test set)

**Fig. 5:**
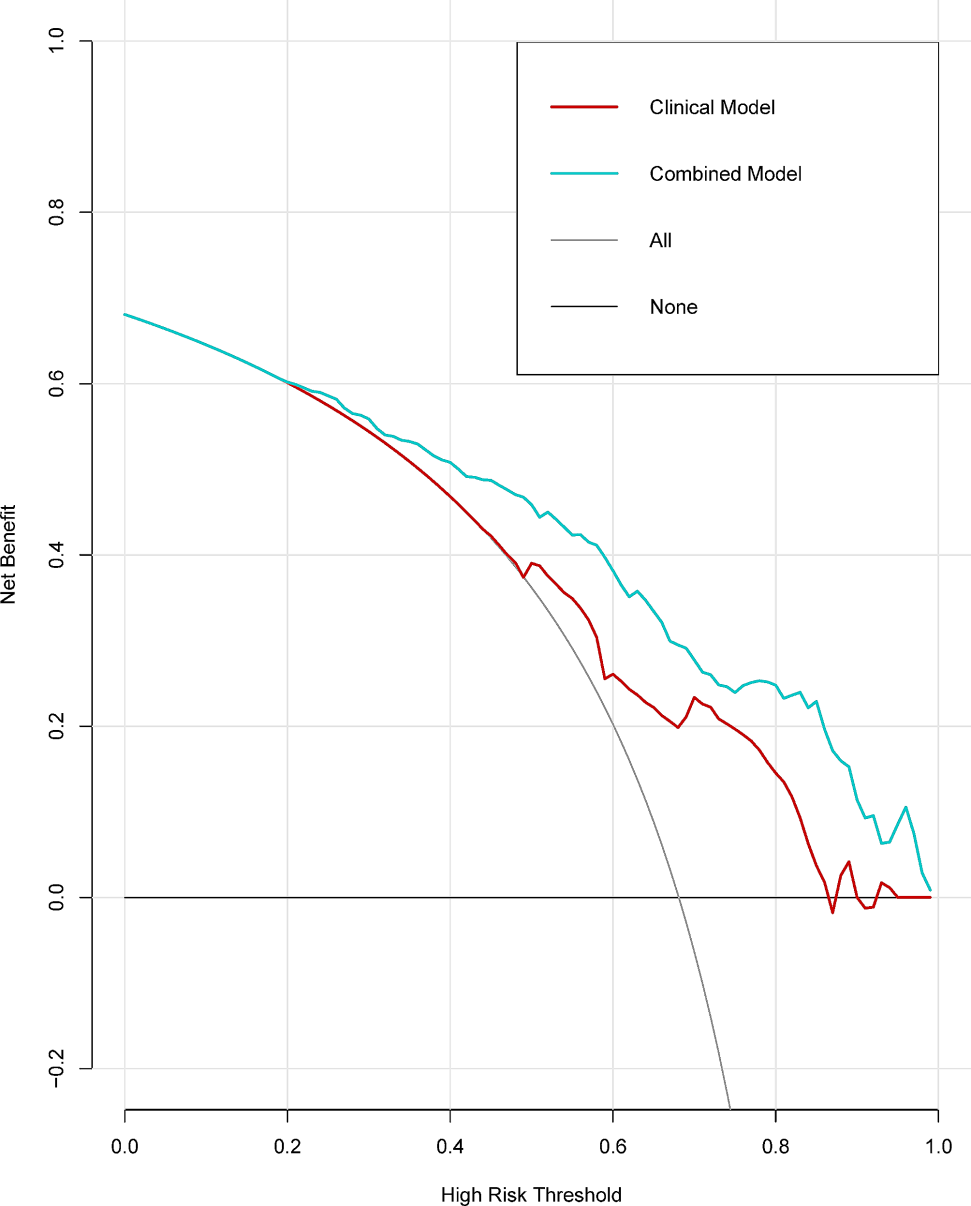
Decision curve analysis for clinical and combined model

The calibration curves of the two models exhibited a good agreement between predicted and actual observed probabilities for both the training and test cohorts (Fig. 4A-D). Decision curve analysis demonstrated that the combined model provided a higher net benefit for predicting GBM OS compared to the clinical model across the threshold probability range of 0.2–1.0 (Fig. 5).

## Discussion

In our study, we simplified certain VASARI features to maximize reproducibility and stability, regardless of the radiologist’s level of experience. Then, after controlling for confounders using PSM, seven VASARI features were shown to be associated with shorter OS in GBM patients, namely F2M (center/bilateral), F12M (poorly-defined), F15M (edema crosses midline), F19 (ependymal invasion), F21 (deep white matter invasion), F22M (nCET tumor crosses midline), and F23M (enhancing tumor crosses midline). Ultimately, the combined model, consisting of clinicopathologic variables and VASARI features, demonstrated superior predictive performance for OS compared to the clinical model.

The VASARI feature set was chosen for investigation in this study due to two primary considerations. Firstly, the 29 VASARI features extracted exclusively from conventional MRI (T1WI, T2WI, FLAIR and T1CE) are standard sequences for the initial diagnosis of suspected tumor cases. These sequences are routinely accessible in both teaching and non-teaching hospitals. Furthermore, the assessment of MRI semantic features is less susceptible to the influence of acquisition equipment, scanning parameters, and post-processing algorithms compared to functional MRI. Despite the promising potential of emerging radiomics in prognostic evaluation, the VASARI feature set offers better practicality and interpretability for routine applications in radiology and neurosurgery.

Previous studies have confirmed the significant role of VASARI features in predicting genotyping and assessing treatment response and prognosis [15, 16, 24–27]. Most of the aforementioned studies were of single-center or single-model MRI data, and VASARI features were assessed by two raters. Any discrepancies were resolved through consultation or by involving a third qualified physician. However, in clinical practice, it is much more common for physicians to assess preoperative MRI from different hospitals independently. Consequently, there is a paucity of research investigating how a radiologist’s level of experience impacts the consistency assessment of VASARI features in a multi-model and multi-parameter setting. The present study addresses this gap and found that: (i) The consistency of radiologist’s assessment was better in the senior group than in the junior group, suggesting that the VASARI feature extraction was experience-dependent. (ii) The features with κ value of < 0.8 in both the senior and junior groups were mainly multicategorical variables such as necrosis, enhancement, edema, and the percentage of degree of tumor resection, as well as confounding features such as multicentricity or multifocality. Similar to our findings, Kim et al [17] and Chen et al [15] also found that some multicategorical VASARI features are challenging to assess with high agreement, consequently restricting their clinical utility. Therefore, only those features with high stability and reproducibility can provide reliable information for the diagnosis and management of glioma [28]. Based on the considerations mentioned above, therefore, we simplified the VASARI features into dichotomous variables. Although the simplified VASARI lose some detailed information, the consistency of feature assessment is significantly improved, in other words, even when independently assessed by low-seniority radiologist, high intra- and inter-observer consistency can still be obtained.

Numerous studies have investigated the association between VASARI features and GBM patient OS, yet a consensus has not been reached. Peeken et al. [26]. conducted VASARI feature extraction in 189 patients with GBM. The results of univariate Cox regression analysis indicated an association between OS and 10 VASARI features. These features include F9 (multifocal or multicentric), F24 (satellites), F19 (ependymal invasion), F21 (deep white matter invasion), F13 (definition of the non-enhancing margin), F26 (extent of resection of enhancing tumor), F27 (extent resection of nCET), F14 (proportion of edema), and F29 & F30 (lesion size). After assessing the significance of variables within the multivariate model, the critical features for predicting OS were identified as F9, F21, F24, and F19. Nicolas Jilwan et al. [29]. analyzed the MRI of 102 GBMs. In the univariable analysis, F1 (tumor location), F2 (side of tumor epicenter), F5 (proportion enhancing), and F10 (T1/FLAIR ratio) were related to OS, but after adjusting for clinical variables (chemotherapy) and HRAS copy number, only F5 was an independent predictor for OS. In another retrospective study cohort of 98 adult GBMs, the findings based on Kaplan-Meier analysis demonstrated a significant association between F1 (tumor location), F6 (proportion nCET), F7 (proportion necrosis), and OS. However, in the multivariate Cox regression analysis, only F6 and F7 were identified as independent predictors of OS [16]. In contrast to previous studies, in the study by Chen et al. [15]. VASARI features were found to be irrelevant to OS after retrospectively analyzing MRI data from 129 cases of GBM. The discrepancies observed in these findings could be due to several factors, including the limited size of the sample, the heterogeneous composition of the study population (comprising both IDH-mutant and wild-type GBM), and the insufficient implementation of rigorous confounding measures. According to the latest edition of the 2021 WHO guidelines for the classification of CNS tumors [18], IDH-mutant GBM was excluded. So, the relationship between VASARI features and OS in wild-type GBM need to be further elucidated. Based on the present study cohort, we identified seven MR semantic features that were associated with shorter patient OS after balancing potential confounders using PSM, namely F2M (center/bilateral), F12M (poorly-defined), F15M (edema crosses midline), F19 (ependymal invasion), F21 (deep white matter invasion), F22M (nCET tumor crosses midline), and F23M (enhancing tumor crosses midline), and this correlation persisted after sensitivity analyses. Notably, in terms of OS prediction, F19 is the top-ranked feature in terms of model-averaged importance among the seven features mentioned above. Consistent with our findings, several studies [30–32] have demonstrated that patients with subventricular zone involvement have a poor prognosis. Furthermore, our previous study [33] utilizing diffusion-weighted imaging and arterial spin labeling imaging to predict the methylation status of the MGMT promoter in IDH wild-type GBM indicated that patients with subventricular zone involvement predominantly exhibited a non-methylated MGMT promoter status and had a dismal prognosis.

There are several limitations in our study. Firstly, our study is a retrospective, single-center study with a small sample size, and the generalizability of its findings needs to be further verified in prospective studies. In addition, our study excluded some cases of all-cause mortality, which may introduce bias. Secondly, this study did not consider the factor of treatment options after tumor recurrence. This was due to the heterogeneity of treatment options available in our center following recurrence. Additionally, a review also highlighted the lack of available treatments to prolong the OS of recurrent GBM [34]. Finally, the radiologists involved in VASARI feature extraction were from the same institution, but consisted of individuals with different levels of experience, resembling actual clinical scenarios.

## Conclusions

In conclusion, the simplified VASARI features have improved reproducibility and stability regardless of the radiologist’s level of experience. After adjusting for confounders, a subset of VASARI features correlates with OS in GBM patients. Ultimately, the combined model constructed by combining clinicopathologic and VASARI features achieved better OS prediction efficacy than the clinical model, but its generalizability needs to be further validated in prospective studies.

### Electronic supplementary material

Below is the link to the electronic supplementary material.

Supplementary Material 1

## Data Availability

No datasets were generated or analysed during the current study.

## Abbreviations

EOR: extent of resection
GBM: glioblastoma
GTR: grossly total resection
IDH: isocitrate dehydrogenase
KPS: karnofsky performance status
MGMT O^6^: methylguanine DNA methyltransferase
MRI: magnetic resonance imaging
OS: overall survival
PSM: propensity score matching
SOC: standard of care
VASARI: visually accessible rembrandt images
