# Supplementary material for: Association between dichotomized VASARI feature and overall survival in glioblastoma patients: a single-institution propensity score matching analysis
Source: Cancer Imaging. 2024 Aug 18;24:109. doi: 10.1186/s40644-024-00754-z (PMC11330608; doi:10.1186/s40644-024-00754-z)
Supplement: Supplementary file 1 — Supplementary Material 1 [file 40644_2024_754_MOESM1_ESM.docx]

# MRI Scanning Protocols

### Table S1 MRI Scanning Protocols

| **Sequences** | **GE Discovery MR750 3.0 T** | **SIEMENS MAGNETOM Aera 1.5 T** | **GE Signa HDxt 1.5 T** | **Philips Ingenia CX 3.0 T** |
| --- | --- | --- | --- | --- |
| **T1WI (Axial)** | TR/TE = 1750/24 msec, matrix size = 256×256, FOV = 240 mm×240 mm, slice thickness = 5 mm, slice spacing = 1.5 mm | TR/TE = 450/10 msec, matrix size = 320×240, FOV = 230 mm×230 mm, slice thickness = 5.5 mm, slice spacing = 0.8 mm | TR/TE = 1750/24 msec, matrix size = 320×224, FOV = 250 mm×250 mm, slice thickness = 5.5 mm, slice spacing = 0.8 mm | TR/TE = 1840/24 msec, matrix size = 240×192, FOV = 240 mm×240 mm, slice thickness = 5 mm, slice spacing = 1.0 mm |
| **T2WI (Axial)** | TR/TE = 4247/93 msec, matrix size = 512×512, FOV = 240 mm×240 mm, slice thickness = 5 mm, slice spacing = 1.5 mm | TR/TE = 4300/109 msec, matrix size = 320×320, FOV = 230 mm×230 mm, slice thickness = 5.5 mm, slice spacing = 0.8 mm | TR/TE = 5340/85 msec, matrix size = 320×250, FOV = 240 mm×240 mm, slice thickness = 5.5 mm, slice spacing = 0.8 mm | TR/TE = 3140/122 msec, matrix size = 384×384, FOV = 230 mm×230 mm, slice thickness = 5 mm, slice spacing = 1.0 mm |
| **FLAIR**  **(Axial)** | TR/TE = 8000/165 msec, matrix size = 256×256, FOV = 240 mm×240 mm, slice thickness = 5 mm, slice spacing = 1.5 mm | TR/TE = 9000/78 msec, matrix size = 256×256, FOV = 230 mm×230 mm, slice thickness = 5 mm, slice spacing = 0.8 mm | TR/TE = 8500/155 msec, matrix size = 256×160, FOV = 240 mm×240 mm, slice thickness = 5.5 mm, slice spacing = 0.8 mm | TR/TE = 7645/130 msec, matrix size = 284×256, FOV = 240 mm×240 mm, slice thickness = 5 mm, slice spacing = 1.0 mm |
| **T2WI**  **(Sagittal)** | TR/TE = 4338/96 msec, matrix size = 384×384, FOV = 240 mm×240 mm, slice thickness = 5 mm, slice spacing = 1.0 mm | TR/TE = 4500/109 msec, matrix size = 320×320, FOV = 230 mm×230 mm, slice thickness = 5 mm, slice spacing = 0.8 mm | TR/TE = 5340/85 msec, matrix size = 320×256, FOV = 250 mm×250 mm, slice thickness = 5.5 mm, slice spacing = 1.0 mm | TR/TE = 3000/111 msec, matrix size = 288×288, FOV = 230 mm×230 mm, slice thickness = 5 mm, slice spacing = 1.0 mm |
| **T1CE**  **(Axial, Sagittal, and**  **Coronal planes)** | TR/TE = 1750/20 msec, matrix size = 320×256, FOV = 240 mm×240 mm, slice thickness = 5 mm, slice spacing = 1.5 mm | TR/TE = 484/7 msec, matrix size = 320×256, FOV = 230 mm×230 mm, slice thickness = 5.5 mm, slice spacing = 0.8 mm | TR/TE = 1750/24 msec, matrix size = 320×192, FOV = 240 mm×240 mm, slice thickness = 5.5 mm, slice spacing = 0.8 mm | TR/TE = 260/4.6 msec, matrix size = 340×276, FOV = 220 mm×220 mm, slice thickness = 5 mm, slice spacing = 1.0 mm |

# VASARI feature set

### Table S2 VASARI feature set definition and classification criteria.

| Feature number | Name | Description | Options |
| --- | --- | --- | --- |
| F1 | Tumor Location | Location of lesion geographic epicenter (not all areas of involvement) | 0 = -  1 = Frontal  2 = Temporal  3 = Insular  4 = Parietal  5 = Occipital  6 = Brainstem  7 = Cerebellum |
| F2 | Side of Tumor Epicenter | Side of lesion epicenter | 0 = -  1 = Right  2 = Center/Bilateral  3 = Left |
| F3 | Eloquent Brain | Does Does the geographic center or the enhancing component involve eloquent cortex (motor, language, vision) or key underlying white matter? | 0 = -  1= None  2 = Speech motor  3 = Speech receptive  4 = Motor  5 = Vision |
| F4 | Enhancement Quality | [None, Mild, Moderate, Marked]. Qualitative degree of contrast enhancement is defined as having all or portions of the tumor that demonstrate significantly higher signal on the postcontrast T1W images compared to precontrast T1W images. | 0 = -  1 = None  2 = Mild/Minimal  3 = Marked/Avid |
| F5 | Proportion Enhancing | [indeterminate, none (0%), <5%, 6-33%, 34-67%, 68-95%, >95%, All (100%)].  What proportion of the entire tumor is enhancing. (Assuming that the entire abnormality may be comprised of: (1) an enhancing component, (2) a non-enhancing component, (3) a necrotic component and (4) a edema component.) | 0 = -  1 = n/a  2 =None (0%)  3 = <5%  4 = 6-33%  5 = 34-67%  6 = 68-95%  7 = >95%  8 =All (100%)  9 = Indeterminate |
| F6 | Proportion nCET | [indeterminate, none (0%), <5%, 6-33%, 34-67%, 68-95%, >95%, All (100%)].  What proportion of the entire tumor is non-enhancing? Nonenhancing tumor is defined as regions of T2W hyperintensity (less than the intensity of cerebrospinal fluid, with corresponding T1W hypointensity) that are associated with mass effect and architectural distortion, including blurring of the gray-white interface. (Assuming that the the entire abnormality may be comprised of: (1) an enhancing component, (2) a non-enhancing component, (3) a necrotic component and (4) a edema component.) | 0 = -  1 = n/a  2 =None (0%)  3 = <5%  4 = 6-33%  5 = 34-67%  6 = 68-95%  7 = >95%  8 =All (100%)  9 = Indeterminate |
| F7 | Proportion Necrosis | [indeterminate, none (0%), <5%, 6-33% , 34-67%, 68-95%, >95%, All (100%)]. (Necrosis is defined as a region within the tumor that does not enhance or shows markedly diminished enhancement, is high on T2W and proton density images, is low on T1W images, and has an irregular border). (Assuming that the the entire abnormality may be comprised of: (1) an enhancing component, (2) a non-enhancing component, (3) a necrotic component and (4) a edema component.) | 0= -  1 = n/a  2 =None (0%)  3 = <5%  4 = 6-33%  5 = 34-67%  6 = 68-95%  7 = >95%  8 =All (100%)  9 = Indeterminate |
| F8 | Cyst(s) | Cysts are well defined, rounded, often eccentric regions of very bright T2W signal and low T1W signal essentially matching CSF signal intensity, with very thin, regular, smooth, nonenhancing or regularly enhancing walls, possibly with thin, regular, internal septations. | 0 = -  1 = No  2 = Yes |
| F9 | Multifocal or Multicentric | Multifocal is defined as having at least one region of tumor, either enhancing or nonenhancing, which is not contiguous with the dominant lesion and is outside the region of signal abnormality (edema) surrounding the dominant mass. This can be defined as those resulting from dissemination or growth by an established route, spread via commissural or other pathways, or via CSF channels or local metastases, whereas Multicentric are widely separated lesions in different lobes or different hemispheres that cannot be attributed to one of the previously mentioned pathways. Gliomatosis refers to generalized neoplastic transformation of the white matter of most of a hemisphere. | 0 = -  1 = n/a  2 = Multifocal  3 = Multicentric  4 = Gliomatosis |
| F10 | T1/FLAIR RATIO | Tumor feature summary. [Mixed, expansive or infiltrative]. Expansive = size of pre-contrast T1abnormality (exclusive of signal intensity) approximates size of FLAIR abnormality. Mixed = Size of T1 abnormality moderately less than FLAIR envelope; Infiltrative = Size of pre-contrast T1 abnormality much smaller than size of FLAIR abnormality. (Use T2 if FLAIR is not provided) | 0 = -  1 = Expansive (T1~FLAIR)  2 = Mixed  (T1<FLAIR)  3 = Infiltrative  (T1<<FLAIR) |
| F11 | Thickness of enhancing margin | The scoring is not applicable if there is no contrast enhancement. If most of the  enhancing rim Is thin, regular, and has homogenous enhancement the grade is thin. If most of the rim demonstrates nodular and/or thick enhancement, the grade is thick. If there is only solid enhancement and no rim, the grade is None. | 0 = -  1 = n/a  2 = None  3 = Thin  4 = Thick/solid |
| F12 | Definition of the enhancing margin | The scoring is not applicable (NA) if there is no contrast enhancement. Assess if most of the outside margin of the enhancement is well defined or poorly defined. | 0= -  1= n/a  2= Well-defined  3= Poorly-defined |
| F13 | Definition of the non-enhancing  margin (e.g.Grade III) | If most of the outside nonenhancing margin of the tumor is well defined and smooth (geographic), versus if the margin is ill-defined and irregular. | 0= -  1= n/a  2= Smooth  3= Irregular |
| F14 | Proportion of Edema | What proportion of the entire abnormality is vasogenic edema? (Edema should be greater in signal than than nCET and somewhat lower in signal than CSF. Pseudopods arecharacteristic of edema). (Assuming that the the entire abnormality may be comprised of: (1) an enhancing component, (2) a non-enhancing component, (3) a necrotic component and (4) a edema component.) | 0 = -  1 = n/a  2 =None (0%)  3 = <5%  4 = 6-33%  5 = 34-67%  6 = 68-95%  7 = >95%  8 =All (100%)  9 = Indeterminate |
| F15 | Edema Crosses Midline | Edema spans white matter commissures extending into contralateral hemisphere.  (exclusive of herniated ipsilateral tissue) | 0 = -  1 = n/a  2 = No  3 = Yes |
| F16 | Hemorrhage | Intrinsic hemorrhage in the tumor matrix. Any intrinsic foci of low signal on T2WI or high signal on T1WI. (Use B0 image if necessary for confirmation.) | 0 = -  1 = No  2 = Yes |
| F17 | Diffusion | Predominantly facilitated or restricted diffusion in the enhancing or nCET portion of the tumor. (Based on ADC map). Equivocal is neither. No ADC, use no -images. Proportion of tissue not relevant. | 0= -  1 = No image  2 = Facilitated  3 = Restricted  4 =Neither/equivocal |
| F18 | Pial invasion | Enhancement of the overlying pia in continuity with enhancing or non-enhancing tumor. | 0 = -  1 = No  2 = Yes |
| F19 | Ependymal invasion | Invasion of any adjacent ependymal surface in continuity with enhancing or non-enhancing tumor matrix. | 0 = -  1 = No  2 = Yes |
| F20 | Cortical involvement | Non-enhancing or enhancing tumor extending to the cortical mantle, or cortex is no longer distinguishable relative to subjacent tumor. | 0 = -  1 = No  2 = Yes |
| F21 | Deep WM invasion | Enhancing or nCET tumor extending into the internal capsule or brainstem. | 0= -  1= No  2= Yes |
| F22 | nCET tumor Crosses Midline | nCET crosses into contralateral hemisphere through white matter commissures (exclusive of herniated ipsilateral tissue). | 0 = -  1 = n/a (no nCET)  2 = No  3 = Yes |
| F23 | Enhancing tumor Crosses Midline | Enhancing tissue crosses into contralateral hemisphere through white matter commisures (exclusive of herniated ipsilateral tissue). | 0 = -  1 = n/a (no nCET)  2 = No  3 = Yes |
| F24 | Satellites | A satellite lesion is an area of enhancement within the region of signal abnormality surrounding the dominant lesion but not contiguous in any part with the major tumor mass. | 0 = -  1 = No  2 = Yes |
| F25 | Calvarial  remodeling | Erosion of inner table of skull (possibly a secondary sign of slow growth) | 0= -  1= No  2= Yes |
| F26 | Extent of resection of enhancing tumor | Using the first postoperative scan (contrast-enhanced MR imaging) assessed for tumor residual. Estimate the proportion of enhancing tumor removed. Total resection of component should be scored 100%. Subtotal resection of enhancing tissue should be scored accordingly. | 0 = -  1 = n/a  2 =None (0%)  3 = <5%  4 = 6-33%  5 = 34-67%  6 = 68-95%  7 = >95%  8 =All (100%)  9 = Indeterminate |
| F27 | Extent resection of nCET | [indeterminate, none (0%), <5%, 6-33%, 34-67%, 68-95%, >95%, All (100%)]. Using the first postoperative scan (contrast-enhanced MR imaging) assessed for tumor residual. Estimate the proportion of non-enhancing tumor removed. Total resection of component should be scored 100%. Subtotal resection of nCET tissue should be scored accordingly. | 0 = -  1 = n/a  2 =None (0%)  3 = <5%  4 = 6-33%  5 = 34-67%  6 = 68-95%  7 = >95%  8 =All (100%)  9 = Indeterminate |
| F28 | Extent resection of vasogenic edema | Using the first postoperative scan (contrast-enhanced MR imaging) assessed for tumor residual. Estimate the proportion of edema removed. Total resection of edema should be scored 100%. Subtotal resection of edema should be scored accordingly. | 0 = -  1 = n/a  2 =None (0%)  3 = <5%  4 = 6-33%  5 = 34-67%  6 = 68-95%  7 = >95%  8 =All (100%)  9 = Indeterminate |
| F29&F30 | Lesion Size | Largest perpendicular (x-y) cross-sectional diameter of T2 signal abnormality (longest dimension X perpendicular dimension) measured on a single axial image only. | 0= -  1= <0.5cm  2= 0.5 cm  3= 1.0 cm  4= 1.5 cm  5= 2.0 cm  6= 2.5 cm  7= 3.0 cm  8= 3.5 cm  9= 4.0 cm  10= 4.5 cm  11= 5.0 cm  12 = 5.5 cm  13= 6.0 cm  14= 6.5 cm  15= 7.0 cm  16= 7.5 cm  17= 8.0 cm  18= >8.0cm |

# Details of PSM

## F2M_Side of Tumor Epicenter (unilateral vs. center/bilateral)

### Table S3 Baseline characteristics of the study population by F2M before and after propensity score matching.

| **Variables** | **Level** |  | **Before Matching** | | | |  |  | **After Matching^✝^** | | |
| --- | --- | --- | --- | --- | --- | --- | --- | --- | --- | --- | --- |
|  |  | **unilateral** | | **center/bilateral** | ***P*** | **SMD** |  | **unilateral** | **center/bilateral** | ***P*** | **SMD** |
| n |  | 313 | | 38 |  |  |  | 97 | 38 |  |  |
| Age | median (IQR) | 58 (52-66) | | 57 (43-63) | **0.035** | -0.354 |  | 57 (46-63) | 57 (43-63) | 0.493 | 0.020 |
| Gender (%) | male | 205 (65.5) | | 23 (60.5) | 0.670 | -0.102 |  | 59 (60.8) | 23 (60.5) | 1.000 | -0.045 |
|  | female | 108 (34.5) | | 15 (39.5) |  | 0.102 |  | 38 (39.2) | 15 (39.5) |  | 0.045 |
| KPS score | median (IQR) | 90 (80-90) | | 90 (80-90) | 0.291 | -0.187 |  | 90 (80-90) | 90 (80-90) | 0.901 | -0.102 |
| EOR (%) | GTR | 222 (70.9) | | 9 (23.7) | **<0.001** | -1.111 |  | 28 (28.9) | 9 (23.7) | 0.695 | -0.021 |
|  | non-GTR | 91 (29.1) | | 29 (76.3) |  | 1.111 |  | 69 (71.1) | 29 (76.3) |  | 0.021 |
| Therapy (%) | SOC | 188 (60.1) | | 16 (42.1) | 0.052 | -0.364 |  | 40 (41.2) | 16 (42.1) | 1.000 | 0.009 |
|  | non-SOC | 125 (39.9) | | 22 (57.9) |  | 0.364 |  | 57 (58.8) | 22 (57.9) |  | -0.009 |
| MGMT_Status (%) | methylation | 174 (55.6) | | 18 (47.4) | 0.430 | -0.165 |  | 50 (51.5) | 18 (47.4) | 0.806 | -0.079 |
|  | unmethylation | 139 (44.4) | | 20 (52.6) |  | 0.165 |  | 47 (48.5) | 20 (52.6) |  | 0.079 |

SMD, standardized mean difference; IQR, interquartile range; KPS, Karnofsky performance status; EOR, extent of resection; GTR, gross total resection; SOC, standard of care; MGMT, O^6^-methylguanine DNA methyltransferase.

✝Propensity score mathing was calculated using a 1:3 ratio-logistic regression with a nearest-neighbour caliper width of 0.1, matching the following variables: age, gender, KPS, therapy, EOR, MGMT_status.

The bold values represent *P* < 0.05.

### Figure S1


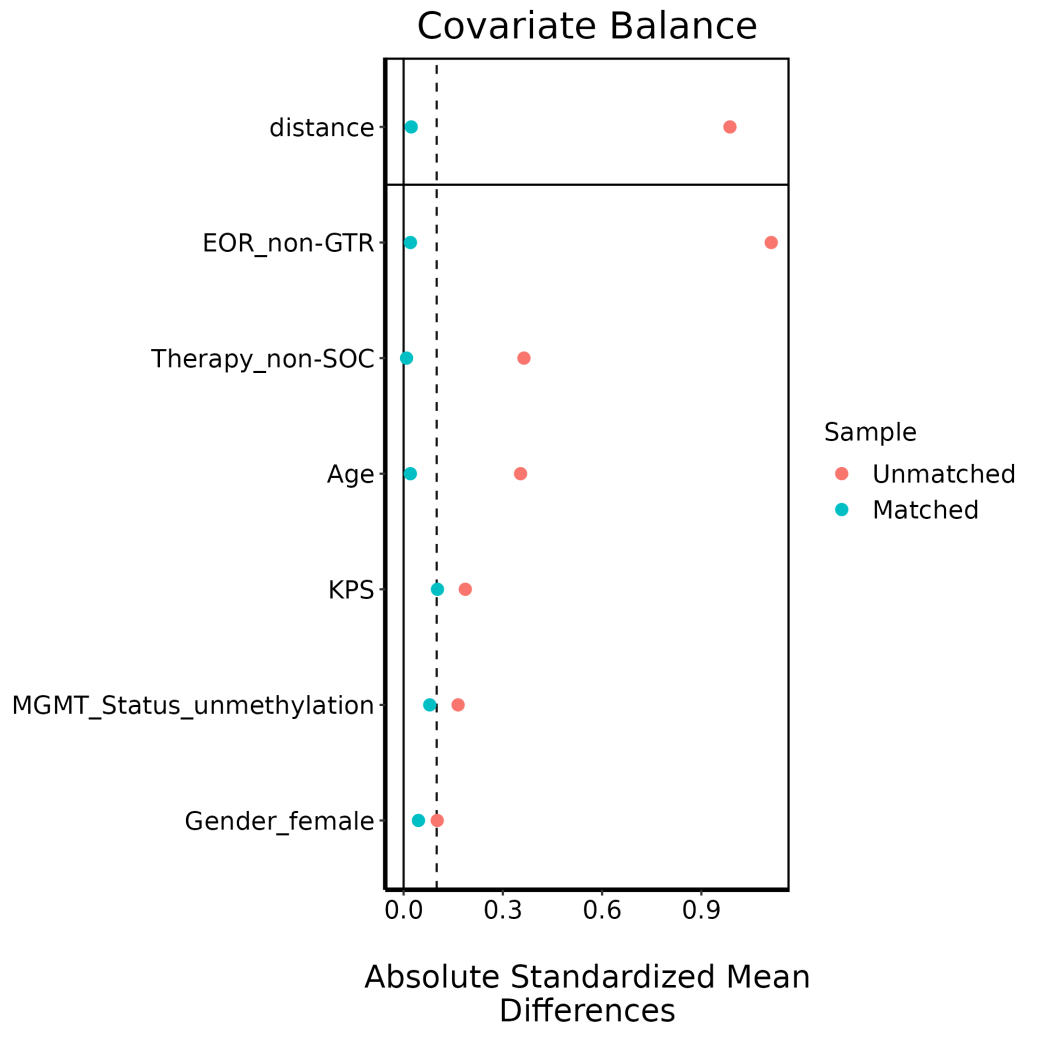

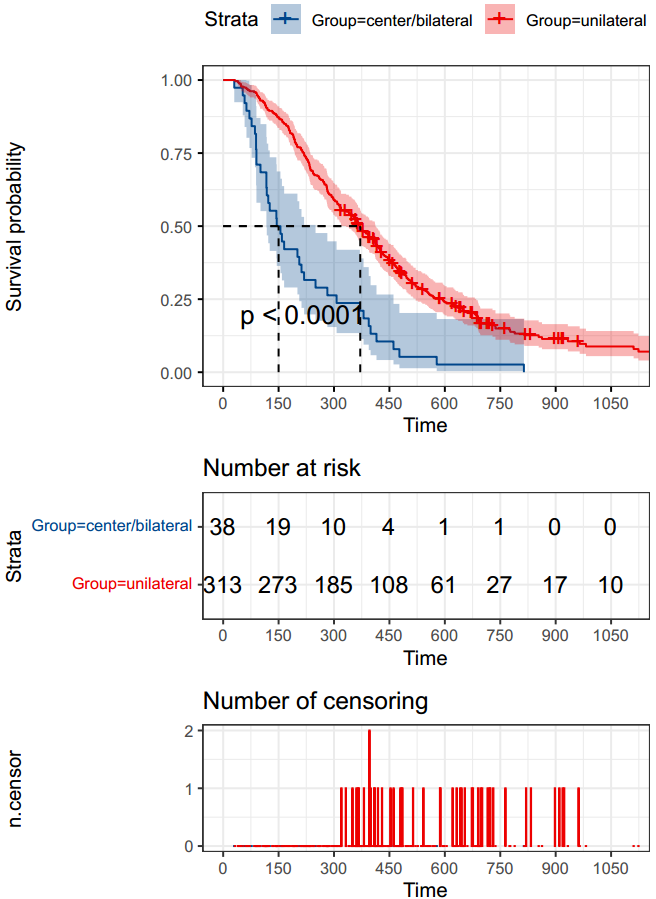

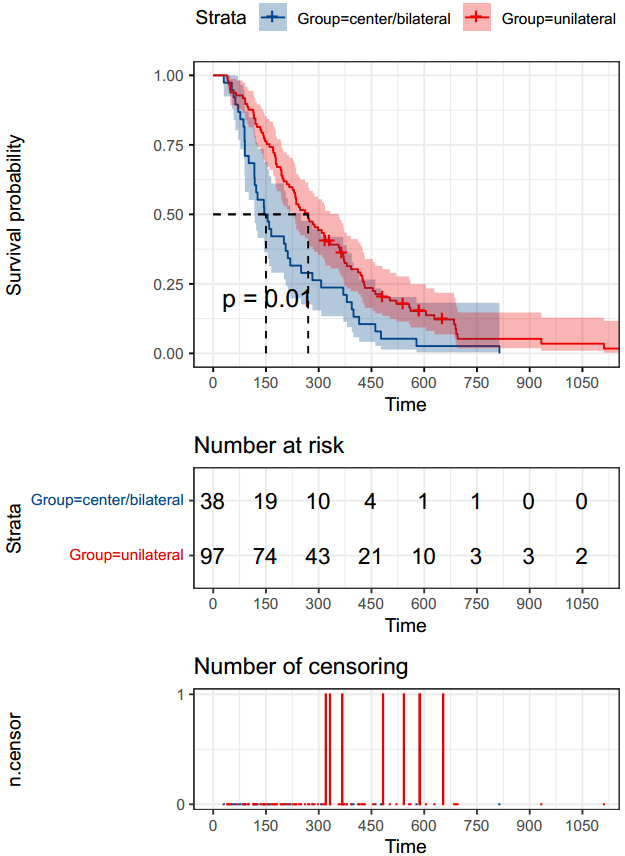


**C**

**B**

**A**

**Figure S1 (A)** love plot: the absolute standard mean differences before matching (red dots) and after matching (green dots). Kaplan-Meier curves for GBM patients stratified by the F2M in both before-matching (**B**) and after-matching (**C**) cohorts.

### Table S4 Sensitivity analysis for F2M after propensity score matching (n=135).

|  | **Univariable** | | | | |  | **Multivariable** | | | | |
| --- | --- | --- | --- | --- | --- | --- | --- | --- | --- | --- | --- |
| **Characteristic** | **N** | **Event N** | **HR** | **95% CI** | ***P*** |  | **N** | **Event N** | **HR** | **95% CI** | ***P*** |
| Age | 135 | 127 | 1.01 | 1.00, 1.03 | 0.070 |  | 135 | 127 | 1.01 | 0.99, 1.02 | 0.411 |
| Gender |  |  |  |  |  |  |  |  |  |  |  |
| female | 53 | 48 | Reference | Reference |  |  | 53 | 48 | Reference | Reference |  |
| male | 82 | 79 | 1.23 | 0.85, 1.77 | 0.268 |  | 82 | 79 | 1.36 | 0.93, 1.98 | 0.117 |
| KPS score | 135 | 127 | 0.95 | 0.92, 0.98 | **<0.001** |  | 135 | 127 | 0.95 | 0.92, 0.98 | **0.004** |
| EOR |  |  |  |  |  |  |  |  |  |  |  |
| GTR | 37 | 34 | Reference | Reference |  |  | 37 | 34 | Reference | Reference |  |
| non-GTR | 98 | 93 | 1.69 | 1.13, 2.53 | **0.010** |  | 98 | 93 | 1.83 | 1.16, 2.91 | **0.010** |
| Therapy |  |  |  |  |  |  |  |  |  |  |  |
| non-SOC | 79 | 79 | Reference | Reference |  |  | 79 | 79 | Reference | Reference |  |
| SOC | 56 | 48 | 0.34 | 0.23, 0.49 | **<0.001** |  | 56 | 48 | 0.25 | 0.17, 0.39 | **<0.001** |
| MGMT_Status |  |  |  |  |  |  |  |  |  |  |  |
| methylation | 68 | 61 | Reference | Reference |  |  | 68 | 61 | Reference | Reference |  |
| unmethylation | 67 | 66 | 1.78 | 1.24, 2.56 | **0.002** |  | 67 | 66 | 1.50 | 1.01, 2.24 | **0.044** |
| F2M |  |  |  |  |  |  |  |  |  |  |  |
| unilateral | 97 | 89 | Reference | Reference |  |  | 97 | 89 | Reference | Reference |  |
| center/bilateral | 38 | 38 | 1.77 | 1.20, 2.60 | **0.004** |  | 38 | 38 | 1.96 | 1.32, 2.89 | **<0.001** |

HR, hazard ratio; CI, confidence interval; KPS, Karnofsky performance status; EOR, extent of resection; GTR, gross total resection; SOC, standard of care; MGMT, O^6^-methylguanine DNA methyltransferase.

The bold values represent *P* < 0.05.

## F12M_Definition of the enhancing margin (poorly-defined vs. well-defined)

### Table S5 Baseline characteristics of the study population by F12M before and after propensity score matching.

| **Variables** | **Level** |  | **Before Matching** | | | |  |  | **After Matching^✝^** | | | |
| --- | --- | --- | --- | --- | --- | --- | --- | --- | --- | --- | --- | --- |
|  |  | **poorly-defined** | | **well-defined** | ***P*** | **SMD** |  | **poorly-defined** | | **well-defined** | ***P*** | **SMD** |
| n |  | 236 | | 115 |  |  |  | 113 | | 113 |  |  |
| Age | median (IQR) | 57 (50-65) | | 60 (54-67) | **0.006** | 0.418 |  | 59 (54-65) | | 59 (54-67) | 0.580 | 0.049 |
| Gender (%) | male | 155 (65.7) | | 73 (63.5) | 0.775 | -0.046 |  | 81 (71.7) | | 71 (62.8) | 0.202 | -0.184 |
|  | female | 81 (34.3) | | 42 (36.5) |  | 0.046 |  | 32 (28.3) | | 42 (37.2) |  | 0.184 |
| KPS score | median (IQR) | 90 (80-90) | | 90 (90-90) | **0.012** | 0.396 |  | 90 (90-90) | | 90 (90-90) | 0.161 | -0.132 |
| EOR (%) | GTR | 143 (60.6) | | 88 (76.5) | **0.005** | 0.376 |  | 87 (77.0) | | 86 (76.1) | 1.000 | -0.021 |
|  | non-GTR | 93 (39.4) | | 27 (23.5) |  | -0.376 |  | 26 (23.0) | | 27 (23.9) |  | 0.021 |
| Therapy (%) | SOC | 127 (53.8) | | 77 (67.0) | **0.026** | 0.279 |  | 72 (63.7) | | 75 (66.4) | 0.780 | 0.056 |
|  | non-SOC | 109 (46.2) | | 38 (33.0) |  | -0.279 |  | 41 (36.3) | | 38 (33.6) |  | -0.056 |
| MGMT_Status (%) | methylation | 124 (52.5) | | 68 (59.1) | 0.294 | 0.134 |  | 67 (59.3) | | 66 (58.4) | 1.000 | -0.018 |
|  | unmethylation | 112 (47.5) | | 47 (40.9) |  | -0.134 |  | 46 (40.7) | | 47 (41.6) |  | 0.018 |

SMD, standardized mean difference; IQR, interquartile range; KPS, Karnofsky performance status; EOR, extent of resection; GTR, gross total resection; SOC, standard of care; MGMT, O^6^-methylguanine DNA methyltransferase.

✝Propensity score mathing was calculated using a 1:1 ratio-logistic regression with a nearest-neighbour caliper width of 0.1, matching the following variables: age, gender, KPS, therapy, EOR, MGMT_status.

The bold values represent *P* < 0.05.

### Figure S2


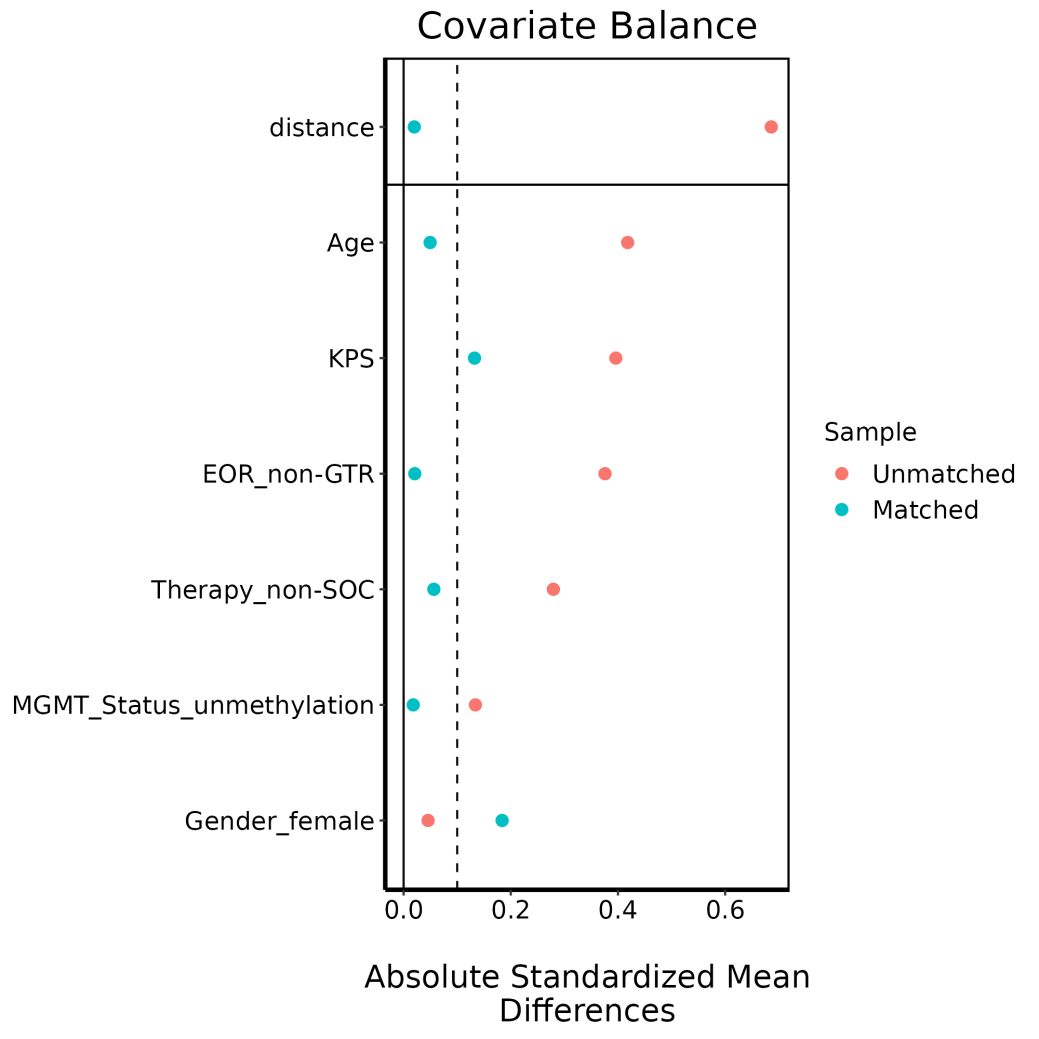

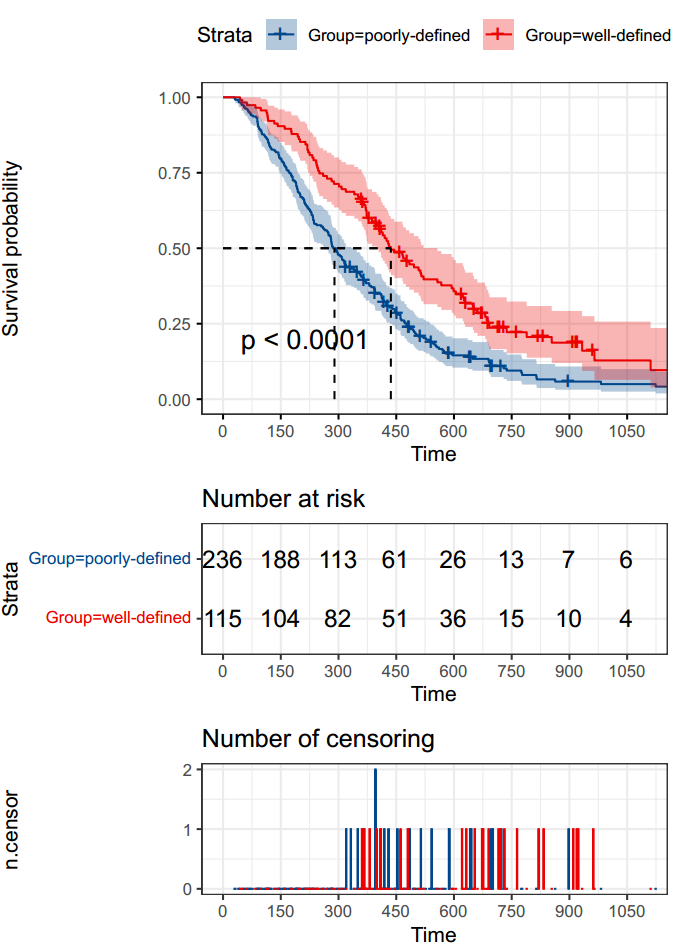

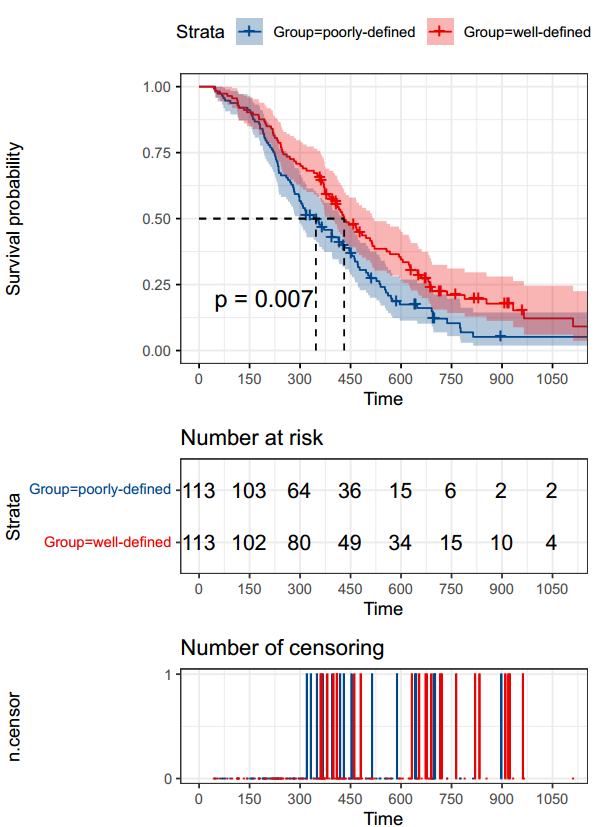


**A**

**C**

**B**

**Figure S2 (A)** love plot: the absolute standard mean differences before matching (red dots) and after matching (green dots). Kaplan-Meier curves for GBM patients stratified by the F12M in both before-matching (**B**) and after-matching (**C**) cohorts.

### Table S6 Sensitivity analysis for F12M after propensity score matching (n=226).

|  | **Univariable** | | | | |  | **Multivariable** | | | | |
| --- | --- | --- | --- | --- | --- | --- | --- | --- | --- | --- | --- |
| **Characteristic** | **N** | **Event N** | **HR** | **95% CI** | ***P*** |  | **N** | **Event N** | **HR** | **95% CI** | ***P*** |
| Age | 226 | 184 | 1.02 | 1.00, 1.03 | 0.056 |  | 226 | 184 | 1.00 | 0.99, 1.02 | 0.737 |
| Gender |  |  |  |  |  |  |  |  |  |  |  |
| male | 152 | 128 | Reference | Reference |  |  | 152 | 128 | Reference | Reference |  |
| female | 74 | 56 | 0.74 | 0.54, 1.02 | 0.066 |  | 74 | 56 | 0.82 | 0.59, 1.14 | 0.237 |
| KPS score | 226 | 184 | 0.97 | 0.94, 0.99 | **0.022** |  | 226 | 184 | 0.98 | 0.95, 1.01 | 0.236 |
| EOR |  |  |  |  |  |  |  |  |  |  |  |
| GTR | 173 | 136 | Reference | Reference |  |  | 173 | 136 | Reference | Reference |  |
| non-GTR | 53 | 48 | 2.07 | 1.48, 2.90 | **<0.001** |  | 53 | 48 | 2.73 | 1.90, 3.94 | **<0.001** |
| Therapy |  |  |  |  |  |  |  |  |  |  |  |
| SOC | 147 | 109 | Reference | Reference |  |  | 147 | 109 | Reference | Reference |  |
| non-SOC | 79 | 75 | 2.36 | 1.75, 3.18 | **<0.001** |  | 79 | 75 | 2.88 | 2.11, 3.93 | **<0.001** |
| MGMT_Status |  |  |  |  |  |  |  |  |  |  |  |
| methylation | 133 | 99 | Reference | Reference |  |  | 133 | 99 | Reference | Reference |  |
| unmethylation | 93 | 85 | 1.73 | 1.29, 2.31 | **<0.001** |  | 93 | 85 | 1.96 | 1.43, 2.68 | **<0.001** |
| F12M |  |  |  |  |  |  |  |  |  |  |  |
| well-defined | 113 | 88 | Reference | Reference |  |  | 113 | 88 | Reference | Reference |  |
| poorly-defined | 113 | 96 | 1.48 | 1.10, 1.98 | **0.009** |  | 113 | 96 | 1.46 | 1.08, 1.96 | **0.013** |

HR, hazard ratio; CI, confidence interval; KPS, Karnofsky performance status; EOR, extent of resection; GTR, gross total resection; SOC, standard of care; MGMT, O^6^-methylguanine DNA methyltransferase.

The bold values represent *P* < 0.05.

## F15M_Edema Crosses Midline (yes vs. no)

### Table S7 Baseline characteristics of the study population by F15M before and after propensity score matching.

| **Variables** | **Level** |  | **Before Matching** | | | |  |  | **After Matching^✝^** | | | |
| --- | --- | --- | --- | --- | --- | --- | --- | --- | --- | --- | --- | --- |
|  |  | **no** | | **yes** | ***P*** | **SMD** |  | **no** | | **yes** | ***P*** | **SMD** |
| n |  | 316 | | 35 |  |  |  | 93 | | 35 |  |  |
| Age | median (IQR) | 58 (52-66) | | 57 (43-64) | 0.134 | -0.270 |  | 57 (47-65) | | 57 (43-64) | 0.621 | -0.002 |
| Gender (%) | male | 206 (65.2) | | 22 (62.9) | 0.930 | -0.048 |  | 60 (64.5) | | 22 (62.9) | 1.000 | -0.020 |
|  | female | 110 (34.8) | | 13 (37.1) |  | 0.048 |  | 33 (35.5) | | 13 (37.1) |  | 0.020 |
| KPS score | median (IQR) | 90 (80-90) | | 90(80-90) | 0.404 | -0.123 |  | 90 (80-90) | | 90 (80-90) | 0.911 | 0.055 |
| EOR (%) | GTR | 219 (69.3) | | 12 (34.3) | **<0.001** | -0.738 |  | 38 (40.9) | | 12 (34.3) | 0.634 | -0.040 |
|  | non-GTR | 97 (30.7) | | 23 (65.7) |  | 0.738 |  | 55 (59.1) | | 23 (65.7) |  | 0.040 |
| Therapy (%) | SOC | 189 (59.8) | | 15 (42.9) | 0.080 | -0.343 |  | 37 (39.8) | | 15 (42.9) | 0.910 | 0.115 |
|  | non-SOC | 127 (40.2) | | 20 (57.1) |  | 0.343 |  | 56 (60.2) | | 20 (57.1) |  | -0.115 |
| MGMT_Status (%) | methylation | 174 (55.1) | | 18 (51.4) | 0.817 | -0.073 |  | 54 (58.1) | | 18 (51.4) | 0.635 | -0.105 |
|  | unmethylation | 142 (44.9) | | 17 (48.6) |  | 0.073 |  | 39 (41.9) | | 17 (48.6) |  | 0.105 |

SMD, standardized mean difference; IQR, interquartile range; KPS, Karnofsky performance status; EOR, extent of resection; GTR, gross total resection; SOC, standard of care; MGMT, O^6^-methylguanine DNA methyltransferase.

✝Propensity score mathing was calculated using a 1:3 ratio-logistic regression with a nearest-neighbour caliper width of 0.1, matching the following variables: age, gender, KPS, therapy, EOR, MGMT_status.

The bold values represent *P* < 0.05.

### Figure S3


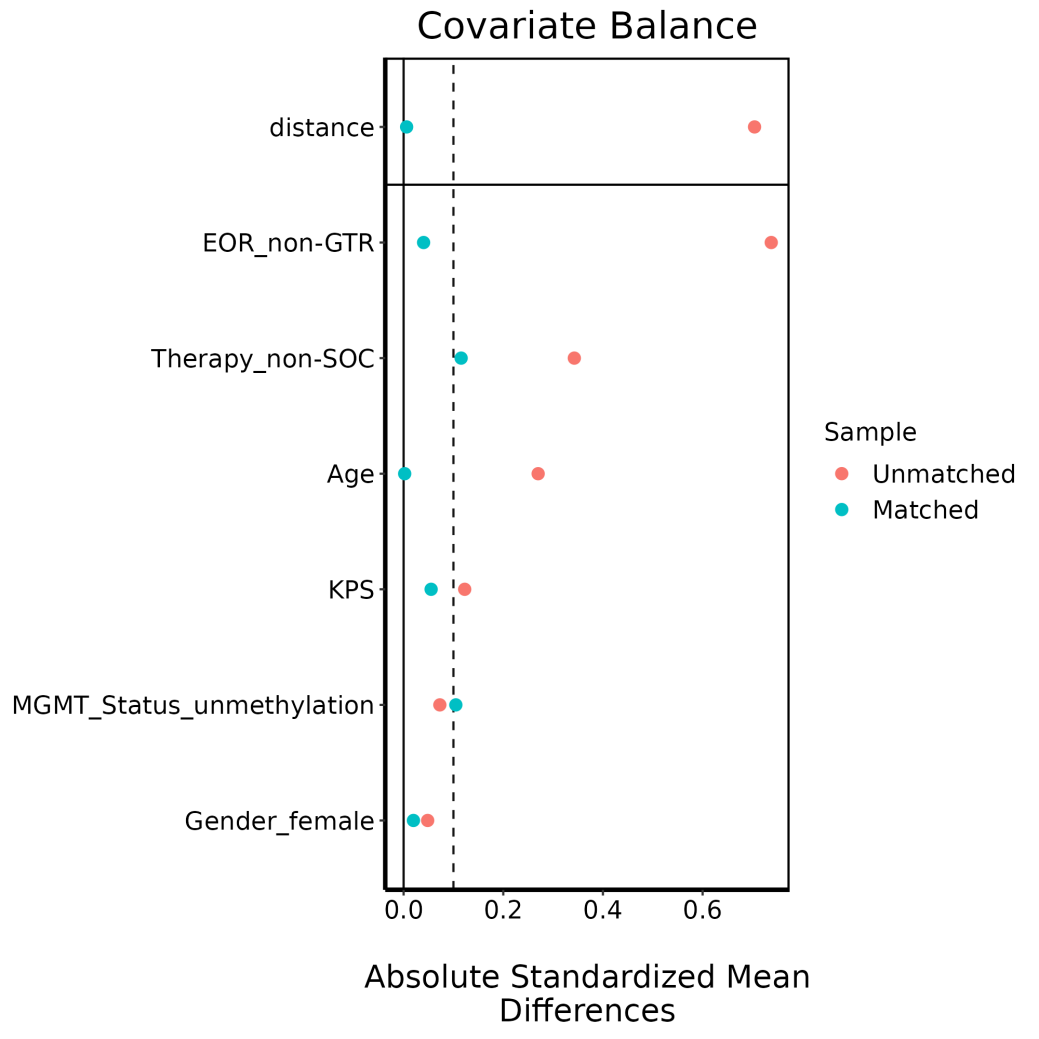

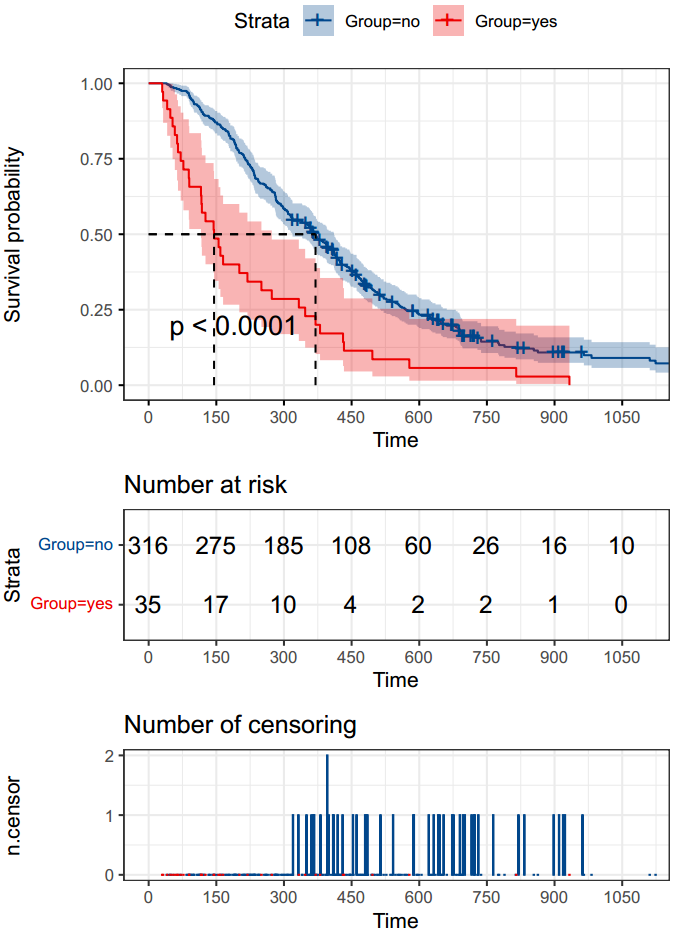

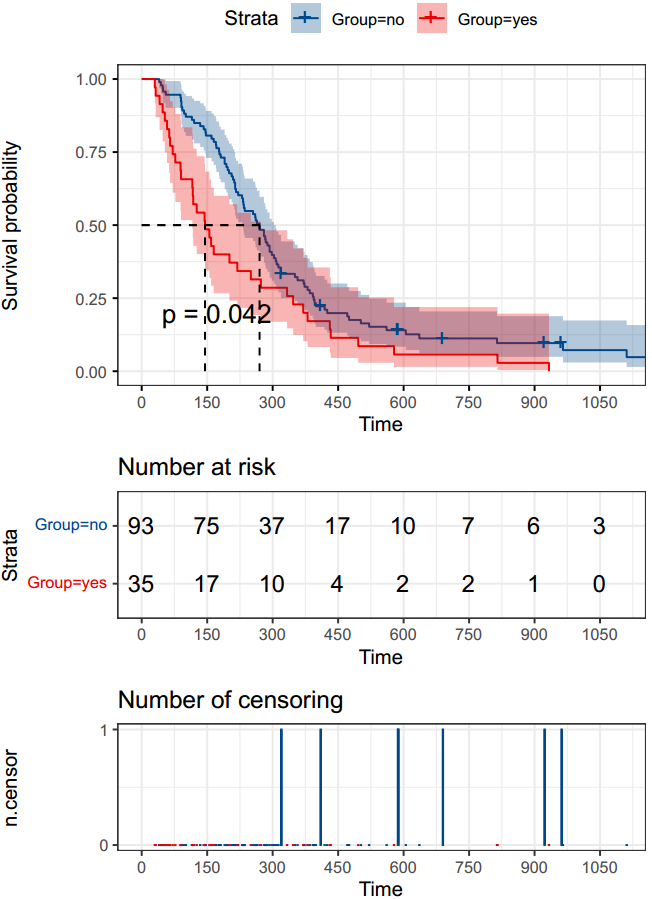


**C**

**A**

**B**

**Figure S3 (A)** love plot: the absolute standard mean differences before matching (red dots) and after matching (green dots). Kaplan-Meier curves for GBM patients stratified by the F15M in both before-matching (**B**) and after-matching (**C**) cohorts.

### Table S8 Sensitivity analysis for F15M after propensity score matching (n=128).

|  | **Univariable** | | | | |  | **Multivariable** | | | | |
| --- | --- | --- | --- | --- | --- | --- | --- | --- | --- | --- | --- |
| **Characteristic** | **N** | **Event N** | **HR** | **95% CI** | ***P*** |  | **N** | **Event N** | **HR** | **95% CI** | ***P*** |
| Age | 128 | 119 | 1.00 | 0.99, 1.02 | 0.521 |  | 128 | 119 | 0.99 | 0.98, 1.01 | 0.225 |
| Gender |  |  |  |  |  |  |  |  |  |  |  |
| male | 82 | 76 | Reference | Reference |  |  | 82 | 76 | Reference | Reference |  |
| female | 46 | 43 | 1.24 | 0.85, 1.81 | 0.263 |  | 46 | 43 | 0.99 | 0.65, 1.51 | 0.975 |
| KPS score | 128 | 119 | 0.94 | 0.92, 0.97 | **<0.001** |  | 128 | 119 | 0.95 | 0.92, 0.99 | **0.005** |
| EOR |  |  |  |  |  |  |  |  |  |  |  |
| GTR | 50 | 45 | Reference | Reference |  |  | 50 | 45 | Reference | Reference |  |
| non-GTR | 78 | 74 | 1.80 | 1.23, 2.64 | **0.003** |  | 78 | 74 | 2.04 | 1.35, 3.10 | **<0.001** |
| Therapy |  |  |  |  |  |  |  |  |  |  |  |
| SOC | 52 | 45 | Reference | Reference |  |  | 52 | 45 | Reference | Reference |  |
| non-SOC | 76 | 74 | 2.09 | 1.44, 3.05 | **<0.001** |  | 76 | 74 | 2.55 | 1.71, 3.80 | **<0.001** |
| MGMT_Status |  |  |  |  |  |  |  |  |  |  |  |
| unmethylation | 56 | 55 | Reference | Reference |  |  | 56 | 55 | Reference | Reference |  |
| methylation | 72 | 64 | 0.58 | 0.40, 0.84 | **0.004** |  | 72 | 64 | 0.72 | 0.49, 1.08 | 0.115 |
| F15M |  |  |  |  |  |  |  |  |  |  |  |
| no | 93 | 84 | Reference | Reference |  |  | 93 | 84 | Reference | Reference |  |
| yes | 35 | 35 | 1.66 | 1.12, 2.48 | **0.012** |  | 35 | 35 | 1.75 | 1.17, 2.63 | **0.007** |

HR, hazard ratio; CI, confidence interval; KPS, Karnofsky performance status; EOR, extent of resection; GTR, gross total resection; SOC, standard of care; MGMT, O^6^-methylguanine DNA methyltransferase.

The bold values represent *P* < 0.05.

## F21_Deep WM invasion (yes vs. no)

### Table S9 Baseline characteristics of the study population by F21 before and after propensity score matching.

| **Variables** | **Level** |  | **Before Matching** | | | |  |  | **After Matching^✝^** | | | |
| --- | --- | --- | --- | --- | --- | --- | --- | --- | --- | --- | --- | --- |
|  |  | **no** | | **yes** | ***P*** | **SMD** |  | **no** | | **yes** | ***P*** | **SMD** |
| n |  | 289 | | 62 |  |  |  | 114 | | 48 |  |  |
| Age | median (IQR) | 59 (53-66) | | 55 (43-65) | **0.014** | -0.357 |  | 57 (49-63) | | 57 (49-65) | 0.852 | 0.065 |
| Gender (%) | male | 192 (66.4) | | 36 (58.1) | 0.268 | -0.170 |  | 68 (59.6) | | 32 (66.7) | 0.508 | 0.155 |
|  | female | 97 (33.6) | | 26 (41.9) |  | 0.170 |  | 46 (40.4) | | 16 (33.3) |  | -0.155 |
| KPS score | median (IQR) | 90 (90-90) | | 90 (80-90) | **<0.001** | -0.464 |  | 90 (80-90) | | 90 (80-90) | 0.296 | -0.009 |
| EOR (%) | GTR | 206 (71.3) | | 25 (40.3) | **<0.001** | -0.631 |  | 68 (59.6) | | 23 (47.9) | 0.230 | -0.078 |
|  | non-GTR | 83 (28.7) | | 37 (59.7) |  | 0.631 |  | 46 (40.4) | | 25 (52.1) |  | 0.078 |
| Therapy (%) | SOC | 178 (61.6) | | 26 (41.9) | **0.007** | -0.398 |  | 65 (57.0) | | 23 (47.9) | 0.374 | -0.099 |
|  | non-SOC | 111 (38.4) | | 36 (58.1) |  | 0.398 |  | 49 (43.0) | | 25 (52.1) |  | 0.099 |
| MGMT_Status (%) | methylation | 159 (55.0) | | 33 (53.2) | 0.907 | -0.036 |  | 68 (59.6) | | 24 (50.0) | 0.338 | -0.097 |
|  | unmethylation | 130 (45.0) | | 29 (46.8) |  | 0.036 |  | 46 (40.4) | | 24 (50.0) |  | 0.097 |

SMD, standardized mean difference; IQR, interquartile range; KPS, Karnofsky performance status; EOR, extent of resection; GTR, gross total resection; SOC, standard of care; MGMT, O6-methylguanine DNA methyltransferase.

✝Propensity score mathing was calculated using a 1:3 ratio-logistic regression with a nearest-neighbour caliper width of 0.1, matching the following variables: age, gender, KPS, therapy, EOR, MGMT status.

The bold values represent *P* < 0.05.

### Figure S4


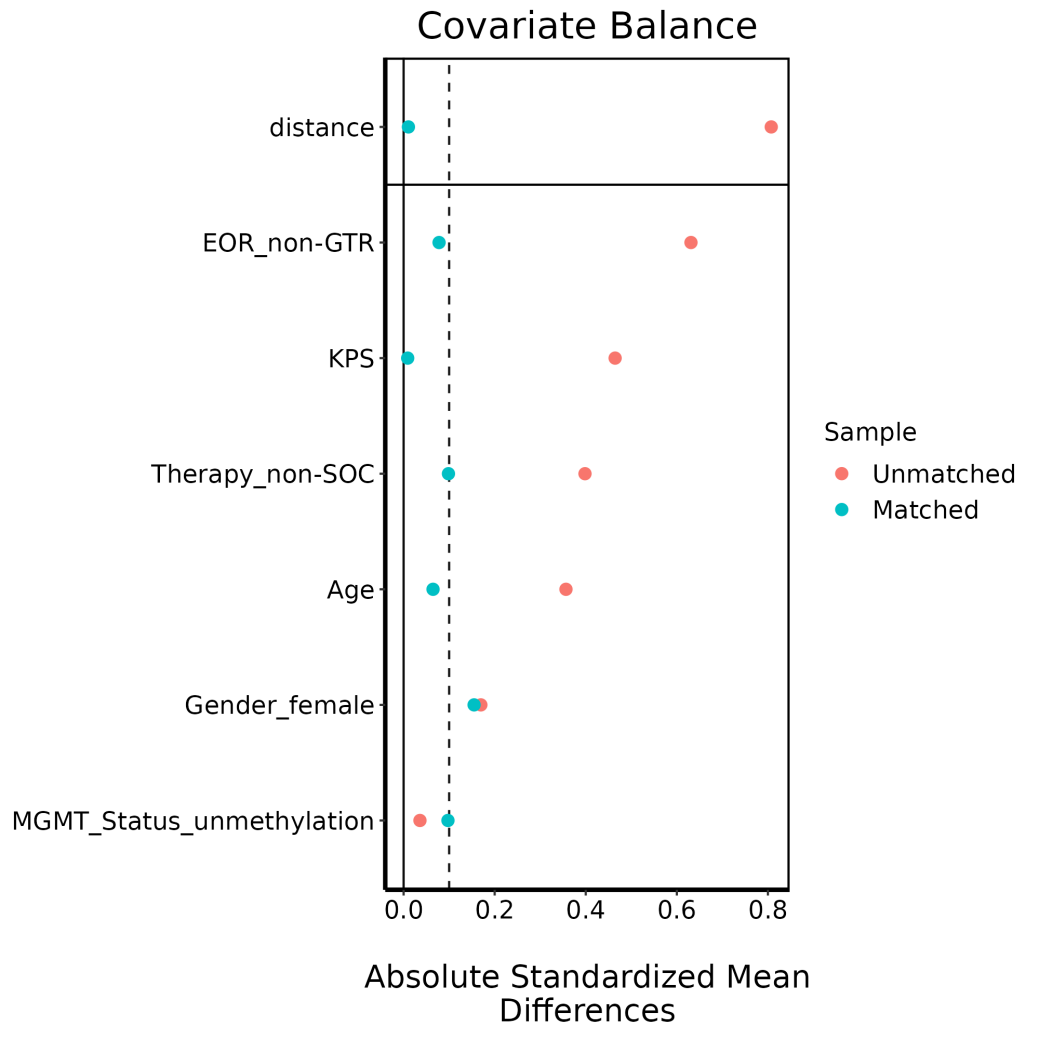

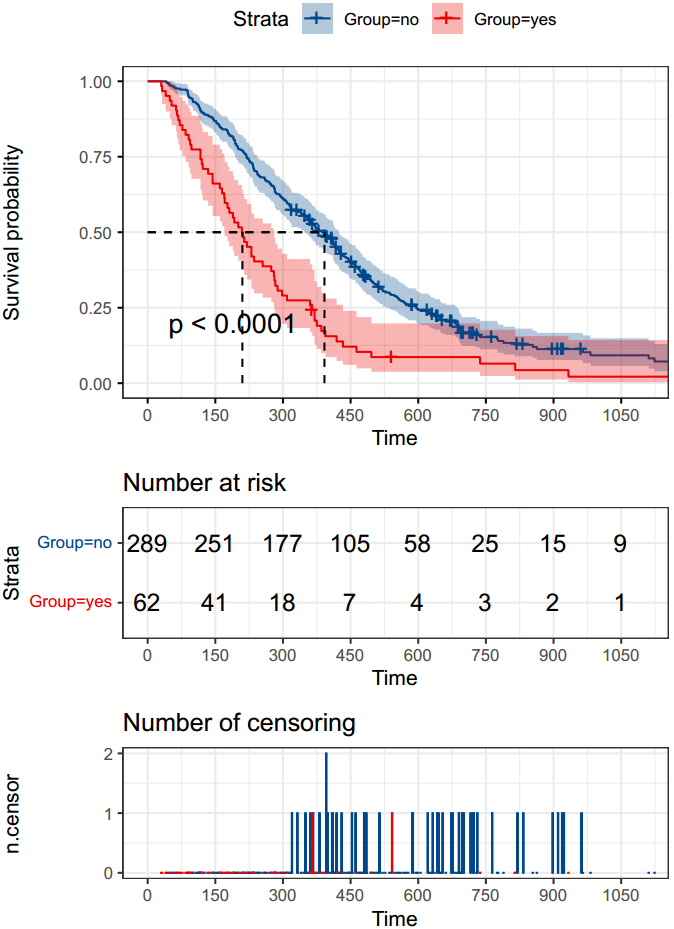

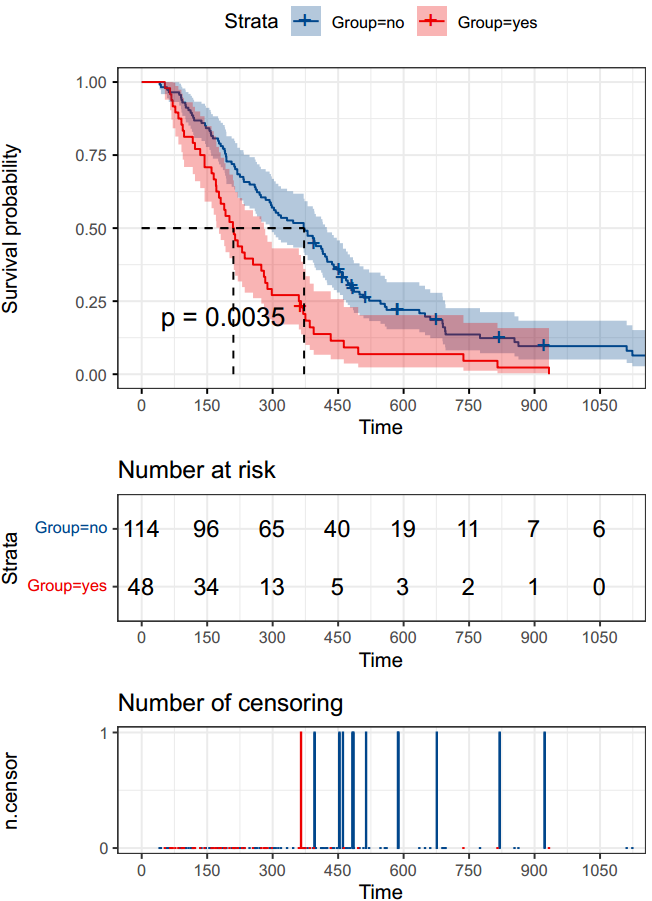


**C**

**A**

**B**

**Figure S4 (A)** love plot: the absolute standard mean differences before matching (red dots) and after matching (green dots). Kaplan-Meier curves for GBM patients stratified by the F21 in both before-matching (**B**) and after-matching (**C**) cohorts.

### Table S10 Sensitivity analysis for F21 after propensity score matching (n=162).

|  | **Univariable** | | | | |  | **Multivariable** | | | | |
| --- | --- | --- | --- | --- | --- | --- | --- | --- | --- | --- | --- |
| **Characteristic** | **N** | **Event N** | **HR** | **95% CI** | ***P*** |  | **N** | **Event N** | **HR** | **95% CI** | ***P*** |
| Age | 162 | 148 | 1.00 | 0.99, 1.02 | 0.552 |  | 162 | 148 | 0.98 | 0.97, 1.00 | 0.045 |
| Gender |  |  |  |  |  |  |  |  |  |  |  |
| male | 100 | 95 | Reference | Reference |  |  | 100 | 95 | Reference | Reference |  |
| female | 62 | 53 | 0.86 | 0.61, 1.20 | 0.380 |  | 62 | 53 | 1.15 | 0.80, 1.65 | 0.442 |
| KPS score | 162 | 148 | 0.96 | 0.94, 0.99 | **0.002** |  | 162 | 148 | 0.96 | 0.93, 0.99 | **0.008** |
| EOR |  |  |  |  |  |  |  |  |  |  |  |
| GTR | 91 | 80 | Reference | Reference |  |  | 91 | 80 | Reference | Reference |  |
| non-GTR | 71 | 68 | 2.16 | 1.54, 3.02 | **<0.001** |  | 71 | 68 | 2.59 | 1.79, 3.74 | **<0.001** |
| Therapy |  |  |  |  |  |  |  |  |  |  |  |
| SOC | 88 | 77 | Reference | Reference |  |  | 88 | 77 | Reference | Reference |  |
| non-SOC | 74 | 71 | 1.96 | 1.42, 2.72 | **<0.001** |  | 74 | 71 | 2.38 | 1.66, 3.43 | **<0.001** |
| MGMT_Status |  |  |  |  |  |  |  |  |  |  |  |
| unmethylation | 70 | 69 | Reference | Reference |  |  | 70 | 69 | Reference | Reference |  |
| methylation | 92 | 79 | 0.55 | 0.40, 0.76 | **<0.001** |  | 92 | 79 | 0.63 | 0.44, 0.89 | **0.009** |
| F21 |  |  |  |  |  |  |  |  |  |  |  |
| no | 114 | 101 | Reference | Reference |  |  | 114 | 101 | Reference | Reference |  |
| yes | 48 | 47 | 2.01 | 1.41, 2.87 | **<0.001** |  | 48 | 47 | 1.59 | 1.09, 2.30 | **0.015** |

HR, hazard ratio; CI, confidence interval; KPS, Karnofsky performance status; EOR, extent of resection; GTR, gross total resection; SOC, standard of care; MGMT, O^6^-methylguanine DNA methyltransferase.

The bold values represent *P* < 0.05.

## F23M_Enhancing tumor Crosses Midline (yes vs. no)

### Table S11 Baseline characteristics of the study population by F23M before and after propensity score matching.

| **Variables** | **Level** |  | **Before Matching** | | | |  |  | **After Matching^✝^** | | | |
| --- | --- | --- | --- | --- | --- | --- | --- | --- | --- | --- | --- | --- |
|  |  | **no** | | **yes** | ***P*** | **SMD** |  | **no** | | **yes** | ***P*** | **SMD** |
| n |  | 315 | | 36 |  |  |  | 126 | | 35 |  |  |
| Age | median (IQR) | 58 (52-66) | | 56 (46-61) | 0.062 | -0.219 |  | 58 (49-64) | | 56 (46-61) | 0.368 | 0.089 |
| Gender (%) | male | 203 (64.4) | | 25 (69.4) | 0.681 | 0.109 |  | 87 (69.0) | | 24 (68.6) | 1.000 | 0.031 |
|  | female | 112 (35.6) | | 11 (30.6) |  | -0.109 |  | 39 (31.0) | | 11 (31.4) |  | -0.031 |
| KPS score | median (IQR) | 90 (80-90) | | 90 (80-90) | 0.100 | -0.266 |  | 90 (80-90) | | 90 (80-90) | 0.998 | 0.085 |
| EOR (%) | GTR | 215 (68.3) | | 16 (44.4) | **0.008** | -0.479 |  | 73 (57.9) | | 16 (45.7) | 0.274 | -0.129 |
|  | non-GTR | 100 (31.7) | | 20 (55.6) |  | 0.479 |  | 53 (42.1) | | 19 (54.3) |  | 0.129 |
| Therapy (%) | SOC | 186 (59.0) | | 18 (50.0) | 0.388 | -0.181 |  | 63 (50.0) | | 18 (51.4) | 1.000 | 0.100 |
|  | non-SOC | 129 (41.0) | | 18 (50.0) |  | 0.181 |  | 63 (50.0) | | 17 (48.6) |  | -0.100 |
| MGMT_Status (%) | methylation | 171 (54.3) | | 21 (58.3) | 0.775 | 0.082 |  | 76 (60.3) | | 20 (57.1) | 0.886 | -0.087 |
|  | unmethylation | 144 (45.7) | | 15 (41.7) |  | -0.082 |  | 50 (39.7) | | 15 (42.9) |  | 0.087 |

SMD, standardized mean difference; IQR, interquartile range; KPS, Karnofsky performance status; EOR, extent of resection; GTR, gross total resection; SOC, standard of care; MGMT, O^6^-methylguanine DNA methyltransferase.

✝Propensity score mathing was calculated using a 1:4 ratio-logistic regression with a nearest-neighbour caliper width of 0.1, matching the following variables: age, gender, KPS, therapy, EOR, MGMT_status.

The bold values represent *P* < 0.05.

### Figure S5


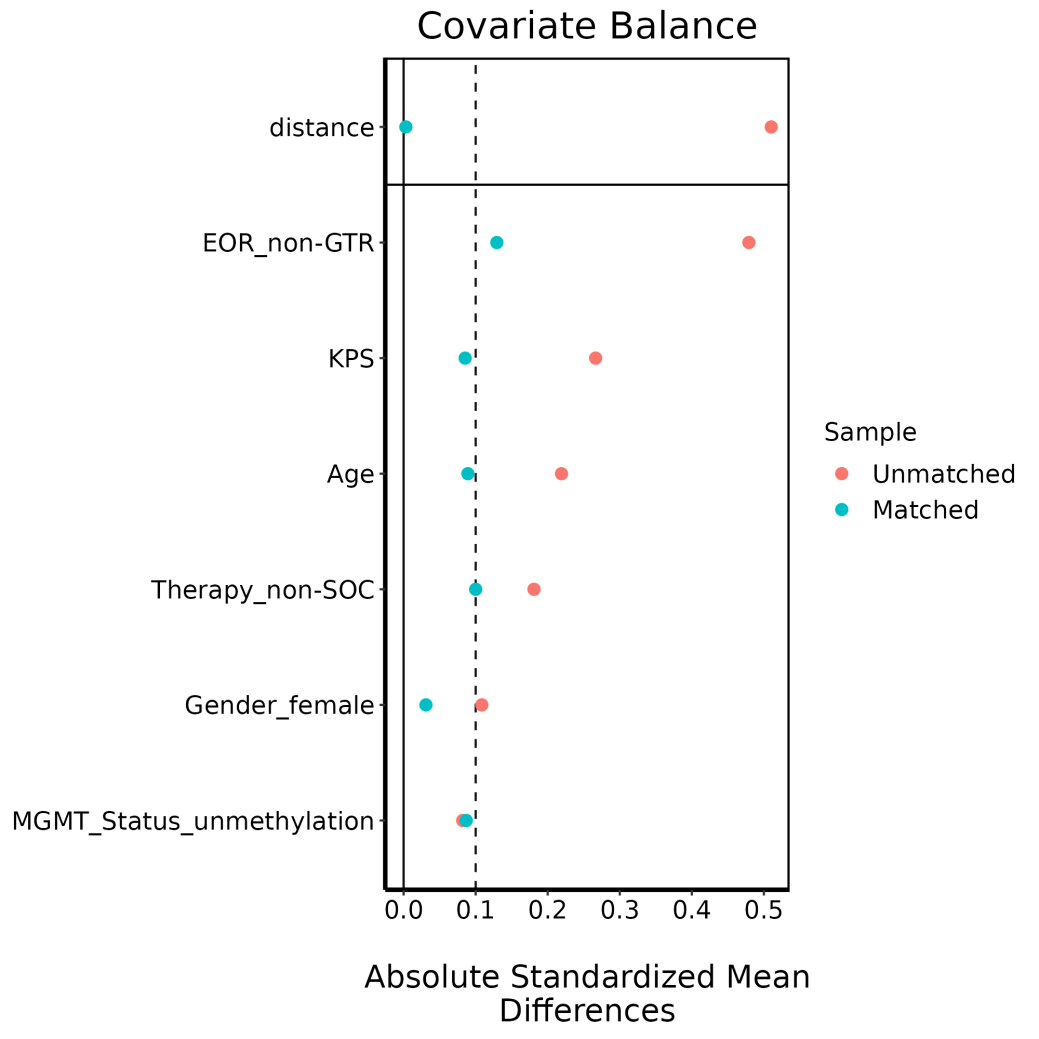

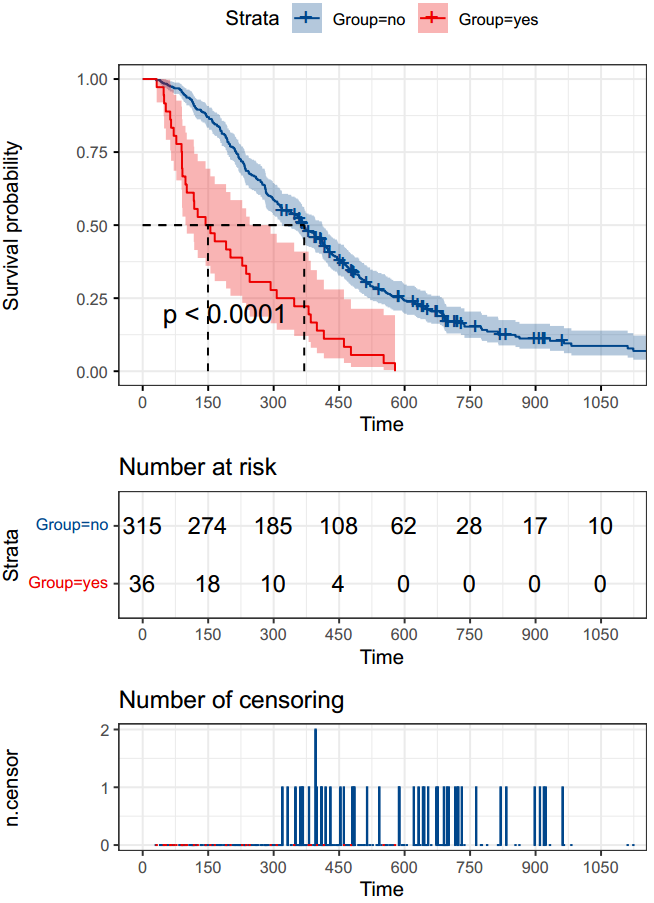

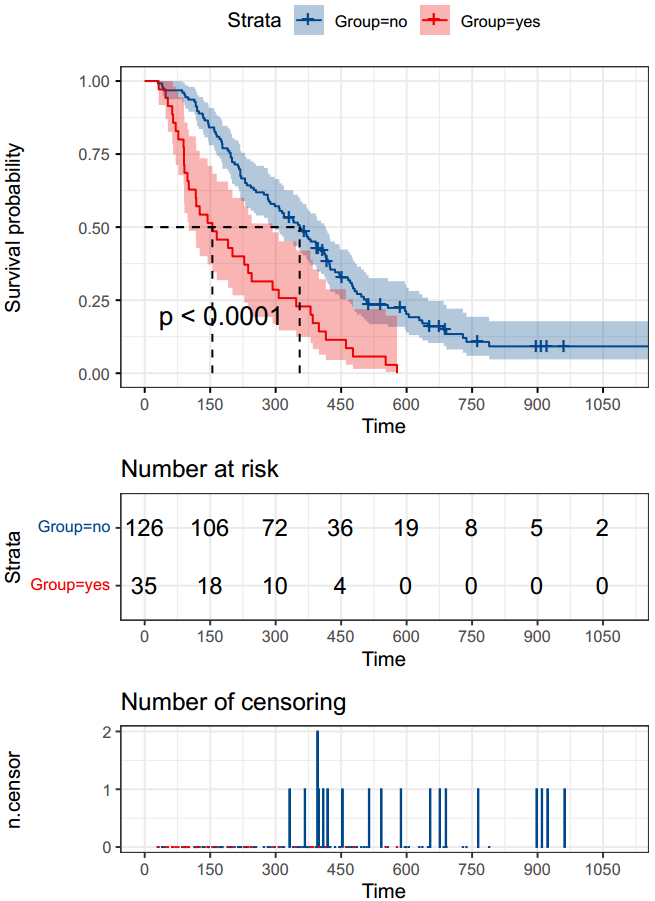


**A**

**C**

**B**

**Figure S5 (A)** love plot: the absolute standard mean differences before matching (red dots) and after matching (green dots). Kaplan-Meier curves for GBM patients stratified by the F23M in both before-matching (**B**) and after-matching (**C**) cohorts.

### Table S12 Sensitivity analysis for F23M after propensity score matching (n=161).

|  | **Univariable** | | | | |  | **Multivariable** | | | | |
| --- | --- | --- | --- | --- | --- | --- | --- | --- | --- | --- | --- |
| **Characteristic** | **N** | **Event N** | **HR** | **95% CI** | ***P*** |  | **N** | **Event N** | **HR** | **95% CI** | ***P*** |
| Age | 161 | 142 | 1.01 | 1.00, 1.03 | 0.085 |  | 161 | 142 | 1.01 | 0.99, 1.02 | 0.502 |
| Gender |  |  |  |  |  |  |  |  |  |  |  |
| male | 111 | 98 | Reference | Reference |  |  | 111 | 98 | Reference | Reference |  |
| female | 50 | 44 | 1.09 | 0.77, 1.56 | 0.622 |  | 50 | 44 | 0.80 | 0.55, 1.16 | 0.243 |
| KPS score | 161 | 142 | 0.95 | 0.92, 0.97 | **<0.001** |  | 161 | 142 | 0.95 | 0.92, 0.98 | **0.003** |
| EOR |  |  |  |  |  |  |  |  |  |  |  |
| GTR | 89 | 73 | Reference | Reference |  |  | 89 | 73 | Reference | Reference |  |
| non-GTR | 72 | 69 | 2.48 | 1.76, 3.49 | **<0.001** |  | 72 | 69 | 2.45 | 1.67, 3.61 | **<0.001** |
| Therapy |  |  |  |  |  |  |  |  |  |  |  |
| SOC | 81 | 64 | Reference | Reference |  |  | 81 | 64 | Reference | Reference |  |
| non-SOC | 80 | 78 | 3.05 | 2.15, 4.32 | **<0.001** |  | 80 | 78 | 4.17 | 2.78, 6.23 | **<0.001** |
| MGMT_Status |  |  |  |  |  |  |  |  |  |  |  |
| unmethylation | 65 | 65 | Reference | Reference |  |  | 65 | 65 | Reference | Reference |  |
| methylation | 96 | 77 | 0.52 | 0.37, 0.73 | **<0.001** |  | 96 | 77 | 0.71 | 0.49, 1.04 | 0.080 |
| F23M |  |  |  |  |  |  |  |  |  |  |  |
| no | 126 | 107 | Reference | Reference |  |  | 126 | 107 | Reference | Reference |  |
| yes | 35 | 35 | 2.50 | 1.69, 3.70 | **<0.001** |  | 35 | 35 | 3.47 | 2.29, 5.27 | **<0.001** |

HR, hazard ratio; CI, confidence interval; KPS, Karnofsky performance status; EOR, extent of resection; GTR, gross total resection; SOC, standard of care; MGMT, O^6^-methylguanine DNA methyltransferase.

The bold values represent *P* < 0.05.

## F19_ Ependymal invasion (yes vs. no)

### Table S13 Baseline characteristics of the study population by F19 before and after propensity score matching.

| **Variables** | **Level** |  | **Before Matching** | | | |  |  | **After Matching^✝^** | | | |
| --- | --- | --- | --- | --- | --- | --- | --- | --- | --- | --- | --- | --- |
|  |  | **yes** | | **no** | ***P*** | **SMD** |  | **yes** | | **no** | ***P*** | **SMD** |
| n |  | 200 | | 151 |  |  |  | 130 | | 130 |  |  |
| Age | median (IQR) | 58 (49-65) | | 58 (53-66) | 0.164 | 0.254 |  | 61 (53-66) | | 59 (53-66) | 0.920 | 0.018 |
| Gender (%) | male | 137 (68.5) | | 91 (60.3) | 0.137 | -0.168 |  | 89 (68.5) | | 81 (62.3) | 0.361 | -0.126 |
|  | female | 63 (31.5) | | 60 (39.7) |  | 0.168 |  | 41 (31.5) | | 49 (37.7) |  | 0.126 |
| KPS score | median (IQR) | 90 (80-90) | | 90 (90-90) | **0.048** | 0.291 |  | 90 (90-90) | | 90 (90-90) | 0.855 | 0.031 |
| EOR (%) | GTR | 108 (54.0) | | 123 (81.5) | **<0.001** | 0.706 |  | 103 (79.2) | | 102 (78.5) | 1.000 | -0.020 |
|  | non-GTR | 92 (46.0) | | 28 (18.5) |  | -0.706 |  | 27 (20.8) | | 28 (21.5) |  | 0.020 |
| Therapy (%) | SOC | 104 (52.0) | | 100 (66.2) | **0.010** | 0.301 |  | 77 (59.2) | | 84 (64.6) | 0.443 | 0.114 |
|  | non-SOC | 96 (48.0) | | 51 (33.8) |  | -0.301 |  | 53 (40.8) | | 46 (35.4) |  | -0.114 |
| MGMT_Status (%) | methylation | 100 (50.0) | | 92 (60.9) | 0.054 | 0.224 |  | 70 (53.8) | | 79 (60.8) | 0.316 | 0.142 |
|  | unmethylation | 100 (50.0) | | 59 (39.1) |  | -0.224 |  | 60 (46.2) | | 51 (39.2) |  | -0.142 |

SMD, standardized mean difference; IQR, interquartile range; KPS, Karnofsky performance status; EOR, extent of resection; GTR, gross total resection; SOC, standard of care; MGMT, O^6^-methylguanine DNA methyltransferase.

✝Propensity score mathing was calculated using a 1:1 ratio-logistic regression with a nearest-neighbour caliper width of 0.1, matching the following variables: age, gender, KPS, therapy, EOR, MGMT_status.

The bold values represent *P* < 0.05.

### Figure S6


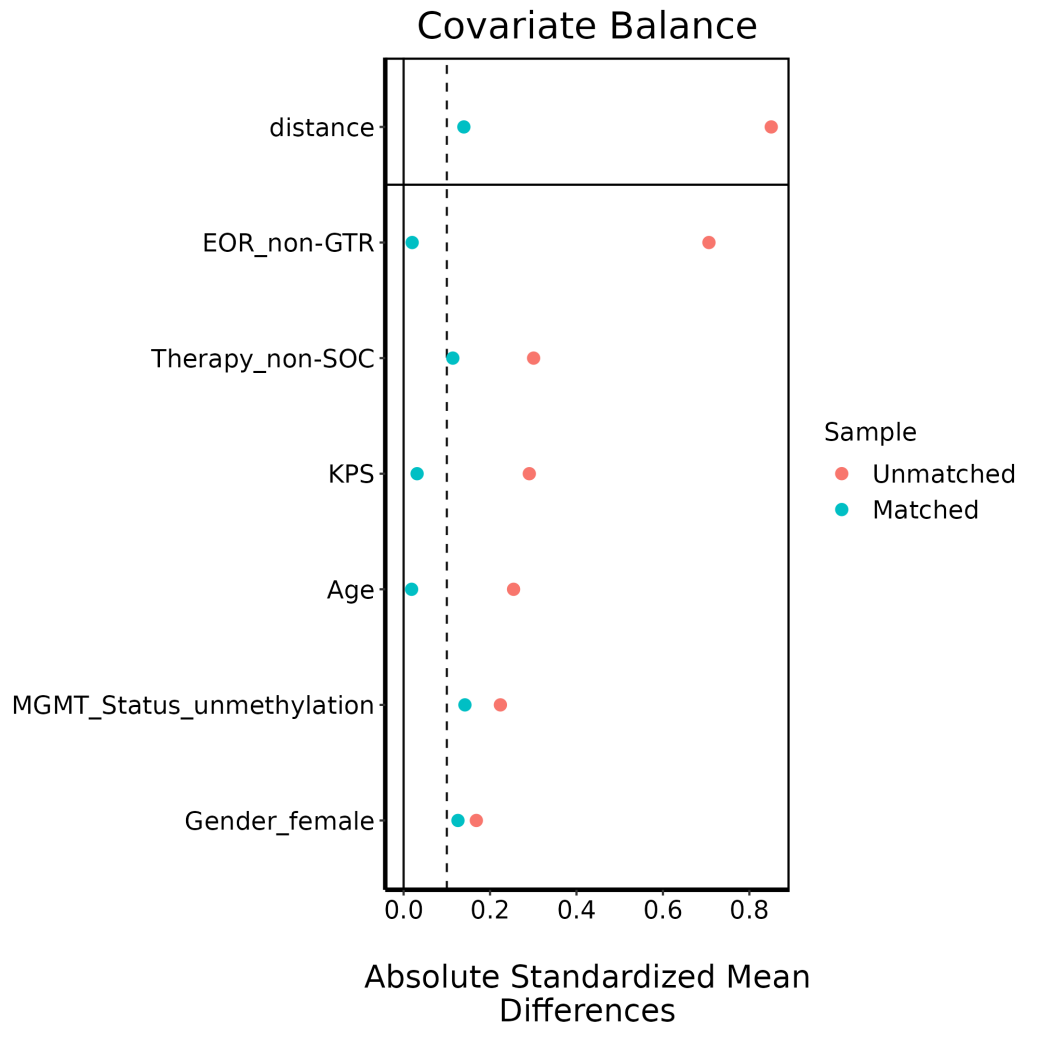

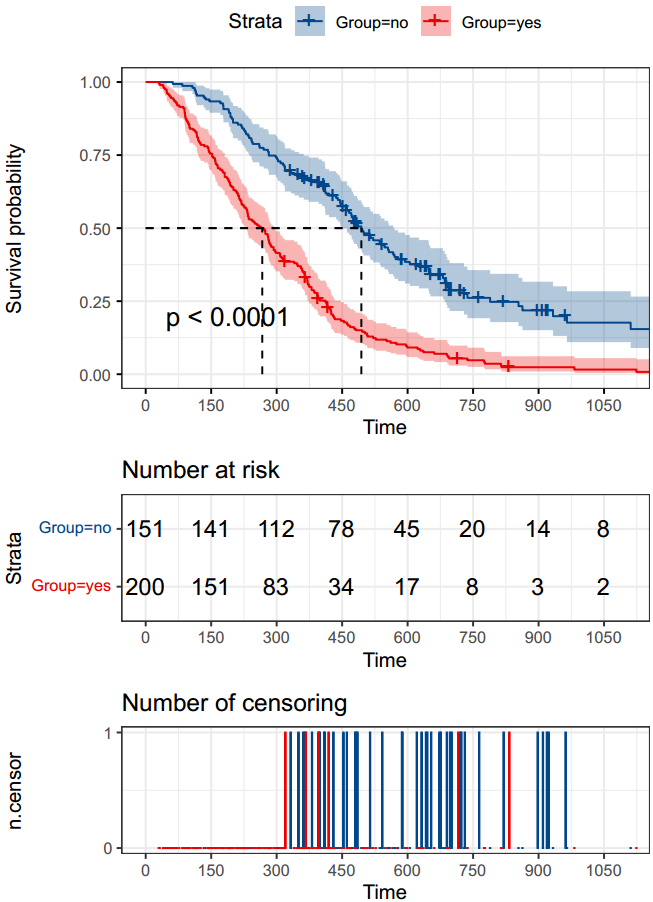

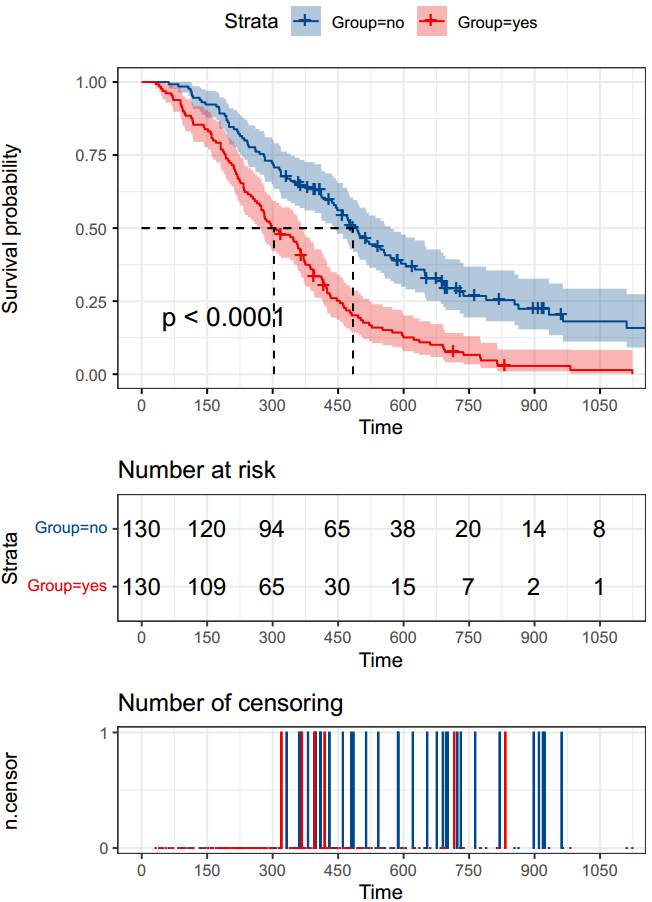


**C**

**A**

**B**

**Figure S6 (A)** love plot: the absolute standard mean differences before matching (red dots) and after matching (green dots). Kaplan-Meier curves for GBM patients stratified by the F19 in both before-matching (**B**) and after-matching (**C**) cohorts.

### Table S14 Sensitivity analysis for F19 after propensity score matching (n=260).

|  | **Univariable** | | | | |  | **Multivariable** | | | | |
| --- | --- | --- | --- | --- | --- | --- | --- | --- | --- | --- | --- |
| **Characteristic** | **N** | **Event N** | **HR** | **95% CI** | ***P*** |  | **N** | **Event N** | **HR** | **95% CI** | ***P*** |
| Age | 260 | 218 | 1.00 | 0.99, 1.02 | 0.460 |  | 260 | 218 | 1.00 | 0.98, 1.01 | 0.457 |
| Gender |  |  |  |  |  |  |  |  |  |  |  |
| male | 170 | 145 | Reference | Reference |  |  | 170 | 145 | Reference | Reference |  |
| female | 90 | 73 | 1.02 | 0.77, 1.35 | 0.906 |  | 90 | 73 | 1.04 | 0.78, 1.39 | 0.778 |
| KPS score | 260 | 218 | 0.96 | 0.94, 0.98 | **0.001** |  | 260 | 218 | 0.97 | 0.95, 1.00 | **0.019** |
| EOR |  |  |  |  |  |  |  |  |  |  |  |
| GTR | 205 | 170 | Reference | Reference |  |  | 205 | 170 | Reference | Reference |  |
| non-GTR | 55 | 48 | 1.78 | 1.28, 2.46 | **<0.001** |  | 55 | 48 | 1.89 | 1.35, 2.64 | **<0.001** |
| Therapy |  |  |  |  |  |  |  |  |  |  |  |
| SOC | 161 | 123 | Reference | Reference |  |  | 161 | 123 | Reference | Reference |  |
| non-SOC | 99 | 95 | 2.60 | 1.98, 3.41 | **<0.001** |  | 99 | 95 | 2.59 | 1.96, 3.42 | **<0.001** |
| MGMT_Status |  |  |  |  |  |  |  |  |  |  |  |
| methylation | 149 | 114 | Reference | Reference |  |  | 149 | 114 | Reference | Reference |  |
| unmethylation | 111 | 104 | 1.62 | 1.24, 2.12 | **<0.001** |  | 111 | 104 | 1.46 | 1.11, 1.92 | **0.007** |
| F19 |  |  |  |  |  |  |  |  |  |  |  |
| no | 130 | 94 | Reference | Reference |  |  | 130 | 94 | Reference | Reference |  |
| yes | 130 | 124 | 2.24 | 1.70, 2.95 | **<0.001** |  | 130 | 124 | 2.02 | 1.53, 2.68 | **<0.001** |

HR, hazard ratio; CI, confidence interval; KPS, Karnofsky performance status; EOR, extent of resection; GTR, gross total resection; SOC, standard of care; MGMT, O^6^-methylguanine DNA methyltransferase.

The bold values represent *P* < 0.05.

## F8_Cyst (yes vs. no)

### Table S15 Baseline characteristics of the study population by F8 before and after propensity score matching.

| **Variables** | **Level** |  | **Before Matching** | | | |  |  | **After Matching^✝^** | | | |
| --- | --- | --- | --- | --- | --- | --- | --- | --- | --- | --- | --- | --- |
|  |  | **no** | | **yes** | ***P*** | **SMD** |  | **no** | | **yes** | ***P*** | **SMD** |
| n |  | 303 | | 48 |  |  |  | 131 | | 48 |  |  |
| Age | median (IQR) | 58 (51-66) | | 58 (53-65) | 0.705 | 0.052 |  | 59 (53-66) | | 58 (53-65) | 0.407 | -0.057 |
| Gender (%) | male | 198 (65.3) | | 30 (62.5) | 0.825 | -0.059 |  | 80 (61.1) | | 30 (62.5) | 0.999 | 0.022 |
|  | female | 105 (34.7) | | 18 (37.5) |  | 0.059 |  | 51 (38.9) | | 18 (37.5) |  | -0.022 |
| KPS score | median (IQR) | 90 (80-90) | | 90 (90-90) | **0.038** | 0.471 |  | 88.02 (4.87) | | 88.54 (4.61) | 0.749 | 0.158 |
| EOR (%) | GTR | 188 (62.0) | | 43 (89.6) | **<0.001** | 0.901 |  | 119 (90.8) | | 43 (89.6) | 0.779 | -0.068 |
|  | non-GTR | 115 (38.0) | | 5 (10.4) |  | -0.901 |  | 12 (9.2) | | 5 (10.4) |  | 0.068 |
| Therapy (%) | SOC | 173 (57.1) | | 31 (64.6) | 0.413 | 0.157 |  | 86 (65.6) | | 31 (64.6) | 1.000 | -0.007 |
|  | non-SOC | 130 (42.9) | | 17 (35.4) |  | -0.157 |  | 45 (34.4) | | 17 (35.4) |  | 0.007 |
| MGMT_Status (%) | methylation | 165 (54.5) | | 27 (56.2) | 0.939 | 0.036 |  | 76 (58.0) | | 27 (56.2) | 0.967 | 0.035 |
|  | unmethylation | 138 (45.5) | | 21 (43.8) |  | -0.036 |  | 55 (42.0) | | 21 (43.8) |  | -0.035 |

SMD, standardized mean difference; IQR, interquartile range; KPS, Karnofsky performance status; EOR, extent of resection; GTR, gross total resection; SOC, standard of care; MGMT, O^6^-methylguanine DNA methyltransferase.

✝Propensity score mathing was calculated using a 1:3 ratio-logistic regression with a nearest-neighbour caliper width of 0.1, matching the following variables: age, gender, KPS, therapy, EOR, MGMT_status.

The bold values represent *P* < 0.05.

### Figure S7


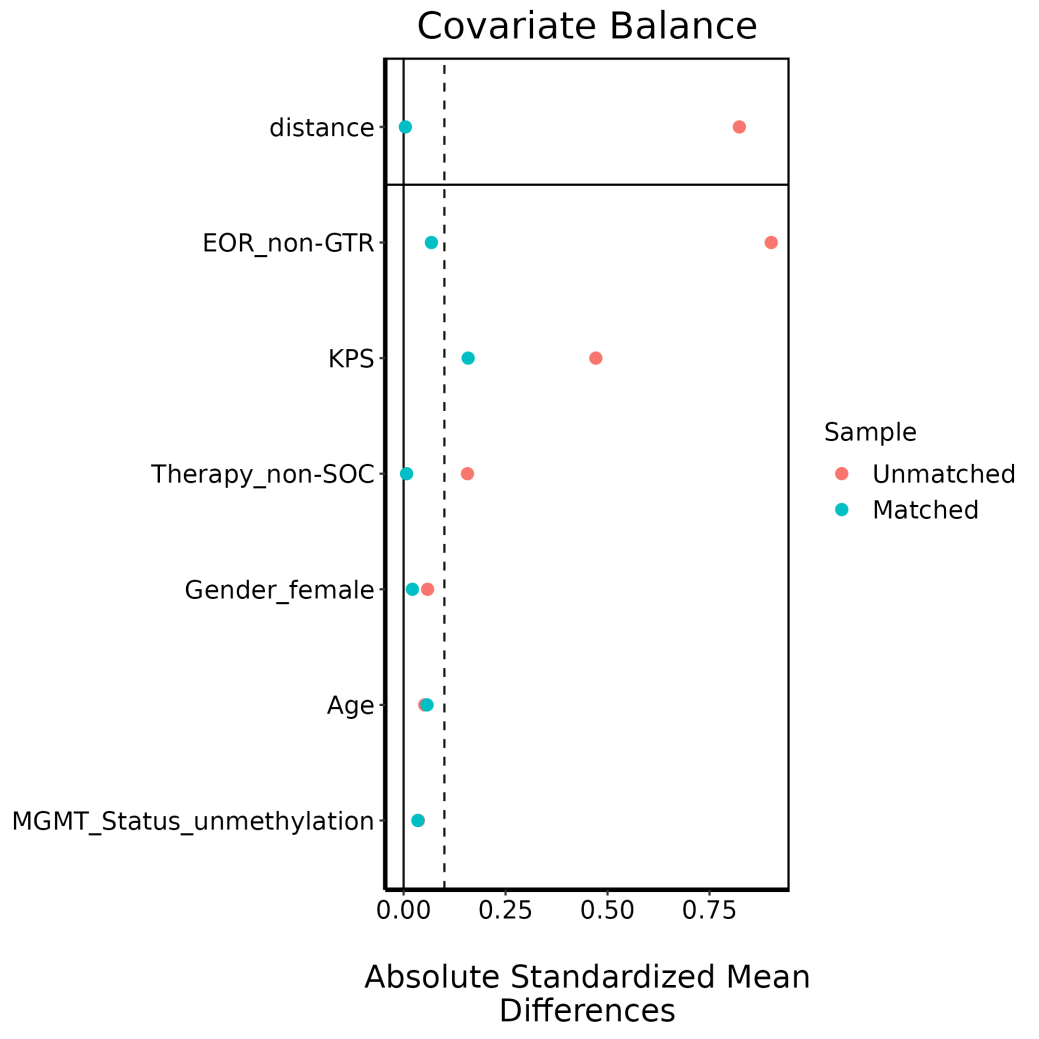

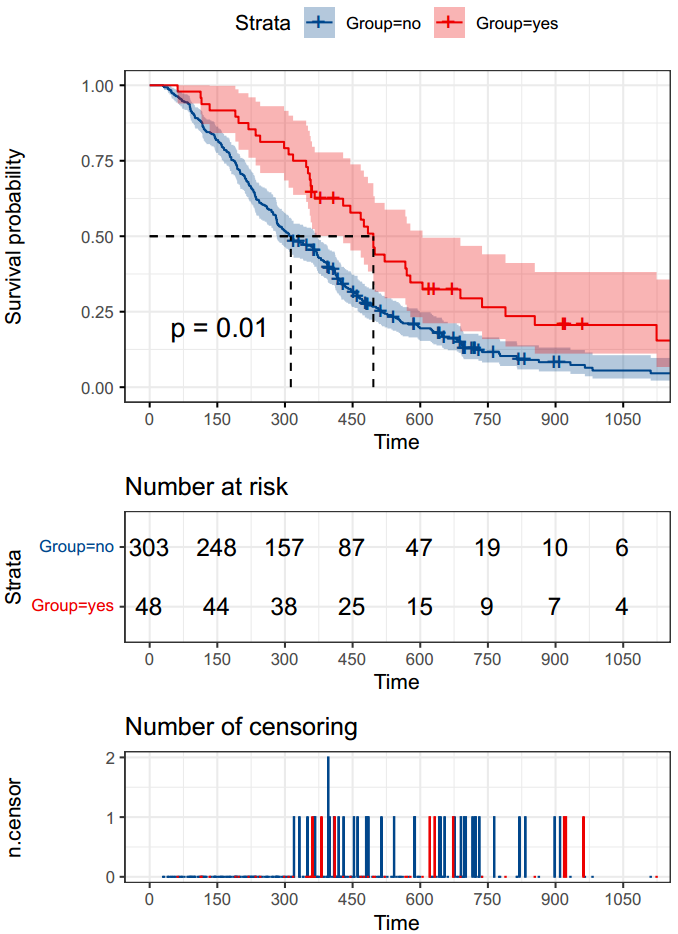

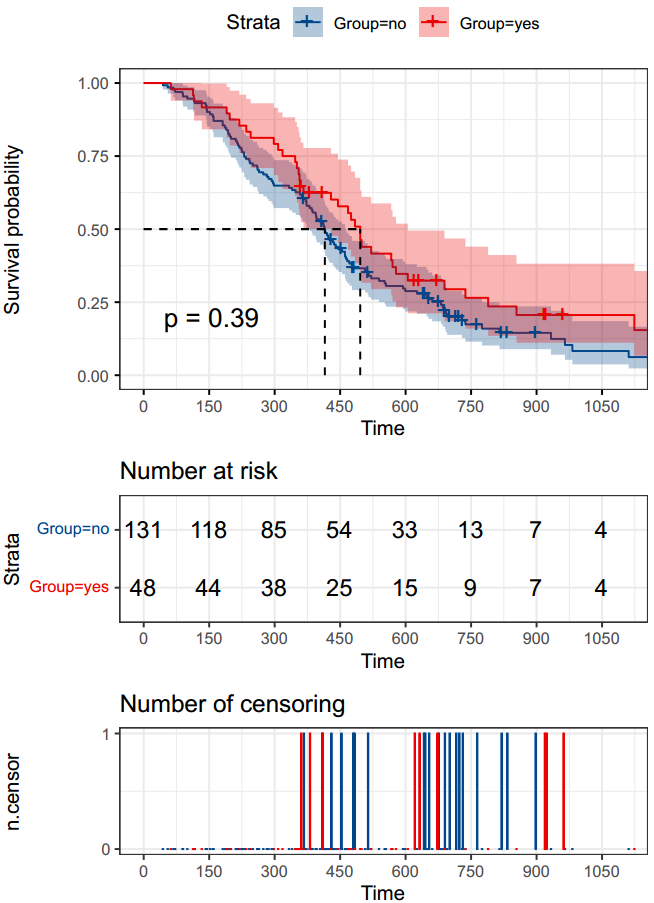


**A**

**C**

**B**

**Figure S7 (A)** love plot: the absolute standard mean differences before matching (red dots) and after matching (green dots). Kaplan-Meier curves for GBM patients stratified by the F8 in both before-matching (**B**) and after-matching (**C**) cohorts.

### Table S16 Sensitivity analysis for F8 after propensity score matching (n=179).

|  | **Univariable** | | | | |  | **Multivariable** | | | | |
| --- | --- | --- | --- | --- | --- | --- | --- | --- | --- | --- | --- |
| **Characteristic** | **N** | **Event N** | **HR** | **95% CI** | ***P*** |  | **N** | **Event N** | **HR** | **95% CI** | ***P*** |
| Age | 179 | 145 | 1.00 | 0.99, 1.02 | 0.826 |  | 179 | 145 | 0.99 | 0.97, 1.00 | 0.148 |
| Gender |  |  |  |  |  |  |  |  |  |  |  |
| male | 110 | 93 | Reference | Reference |  |  | 110 | 93 | Reference | Reference |  |
| female | 69 | 52 | 0.80 | 0.57, 1.12 | 0.188 |  | 69 | 52 | 0.77 | 0.54, 1.10 | 0.145 |
| KPS score | 179 | 145 | 0.97 | 0.93, 1.00 | 0.056 |  | 179 | 145 | 0.99 | 0.95, 1.03 | 0.583 |
| EOR |  |  |  |  |  |  |  |  |  |  |  |
| GTR | 162 | 129 | Reference | Reference |  |  | 162 | 129 | Reference | Reference |  |
| non-GTR | 17 | 16 | 2.04 | 1.21, 3.46 | **0.008** |  | 17 | 16 | 2.26 | 1.33, 3.86 | **0.003** |
| Therapy |  |  |  |  |  |  |  |  |  |  |  |
| SOC | 117 | 86 | Reference | Reference |  |  | 117 | 86 | Reference | Reference |  |
| non-SOC | 62 | 59 | 2.45 | 1.75, 3.43 | **<0.001** |  | 62 | 59 | 2.74 | 1.92, 3.91 | **<0.001** |
| MGMT_Status |  |  |  |  |  |  |  |  |  |  |  |
| methylation | 103 | 78 | Reference | Reference |  |  | 103 | 78 | Reference | Reference |  |
| unmethylation | 76 | 67 | 1.65 | 1.19, 2.29 | **0.003** |  | 76 | 67 | 1.76 | 1.25, 2.48 | **0.001** |
| F8 |  |  |  |  |  |  |  |  |  |  |  |
| no | 131 | 109 | Reference | Reference |  |  | 131 | 109 | Reference | Reference |  |
| yes | 48 | 36 | 0.73 | 0.50, 1.07 | 0.103 |  | 48 | 36 | 0.69 | 0.47, 1.01 | 0.057 |

HR, hazard ratio; CI, confidence interval; KPS, Karnofsky performance status; EOR, extent of resection; GTR, gross total resection; SOC, standard of care; MGMT, O^6^-methylguanine DNA methyltransferase.

The bold values represent *P* < 0.05.

## F9M_Multifocal or Multicentric (none vs. Multifocal/multicentric)

### Table S17 Baseline characteristics of the study population by F9M before and after propensity score matching.

| **Variables** |  |  | | **Before Matching** | | | |  |  | | **After Matching^✝^** | | | |
| --- | --- | --- | --- | --- | --- | --- | --- | --- | --- | --- | --- | --- | --- | --- |
|  | **level** | | **none** | | **Multifocal**  **/multicentric** | ***P*** | **SMD** |  | **none** | | | **Multifocal**  **/multicentric** | ***P*** | **SMD** |
| n |  | | 286 | | 65 |  |  |  | 142 | | | 62 |  |  |
| Age | median (IQR) | | 58 (51-65) | | 58 (51-66) | 0.981 | -0.014 |  | 58 (51-66) | | | 58 (49-66) | 0.836 | 0.045 |
| Gender (%) | male | | 185 (64.7) | | 43 (66.2) | 0.936 | 0.031 |  | 85 (59.9) | | | 40 (64.5) | 0.637 | 0.017 |
|  | female | | 101 (35.3) | | 22 (33.8) |  | -0.031 |  | 57 (40.1) | | | 22 (35.5) |  | -0.017 |
| KPS score | median (IQR) | | 90 (80-90) | | 90 (80-90) | **0.012** | -0.318 |  | 90 (80-90) | | | 90 (80-90) | 0.726 | 0.044 |
| EOR (%) | GTR | | 207 (72.4) | | 24 (36.9) | **<0.001** | -0.735 |  | 69 (48.6) | | | 23 (37.1) | 0.172 | 0.000 |
|  | non-GTR | | 79 (27.6) | | 41 (63.1) |  | 0.735 |  | 73 (51.4) | | | 39 (62.9) |  | -0.000 |
| Therapy (%) | SOC | | 170 (59.4) | | 34 (52.3) | 0.361 | -0.143 |  | 68 (47.9) | | | 33 (53.2) | 0.583 | 0.194 |
|  | non-SOC | | 116 (40.6) | | 31 (47.7) |  | 0.143 |  | 74 (52.1) | | | 29 (46.8) |  | -0.194 |
| MGMT_Status (%) | methylation | | 162 (56.6) | | 30 (46.2) | 0.163 | -0.210 |  | | 74 (52.1) | | 28 (45.2) | 0.447 | -0.049 |
|  | unmethylation | | 124 (43.4) | | 35 (53.8) |  | 0.210 |  | | 68 (47.9) | | 34 (54.8) |  | 0.049 |

SMD, standardized mean difference; IQR, interquartile range; KPS, Karnofsky performance status; EOR, extent of resection; GTR, gross total resection; SOC, standard of care; MGMT, O^6^-methylguanine DNA methyltransferase.

✝Propensity score mathing was calculated using a 1:3 ratio-logistic regression with a nearest-neighbour caliper width of 0.1, matching the following variables: age, gender, KPS, therapy, EOR, MGMT status.

The bold values represent *P* < 0.05.

### Figure S8


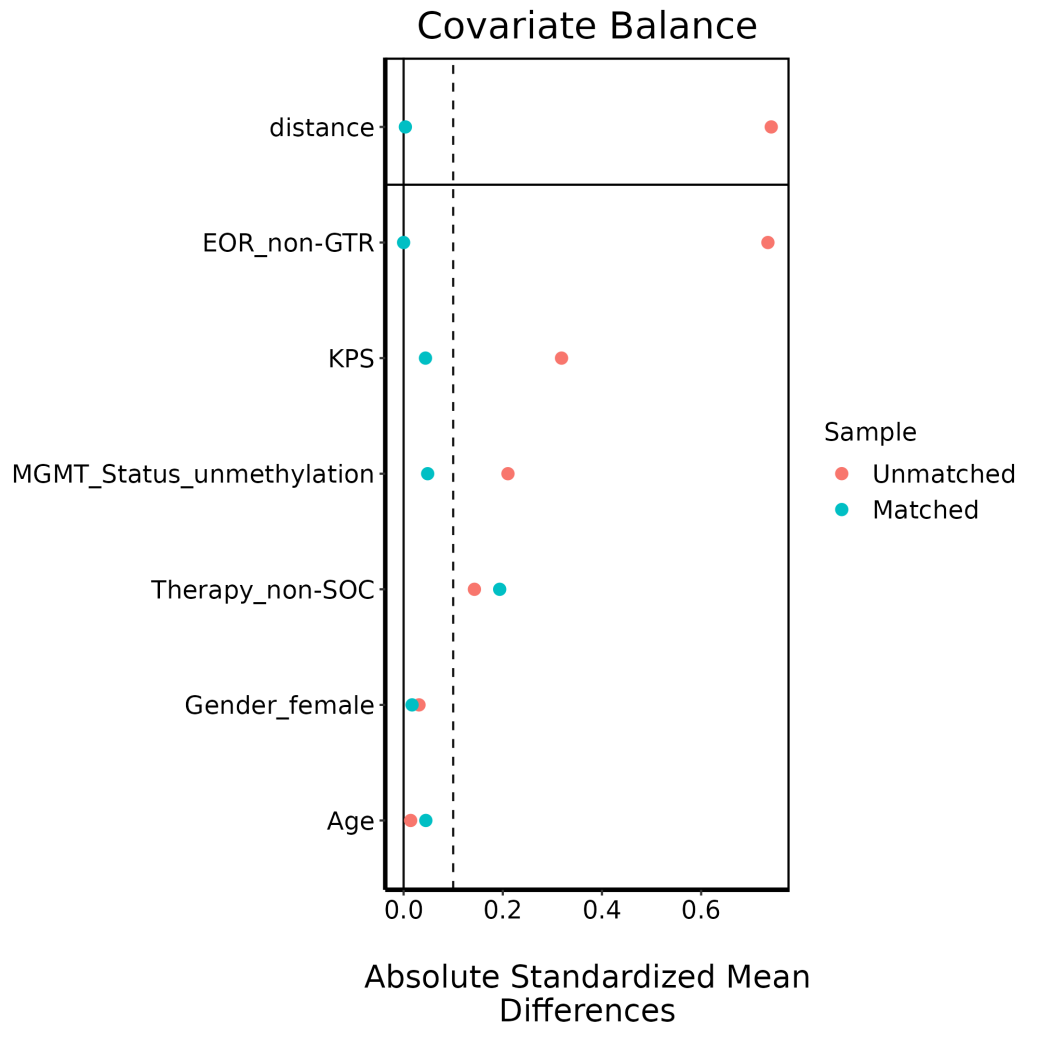

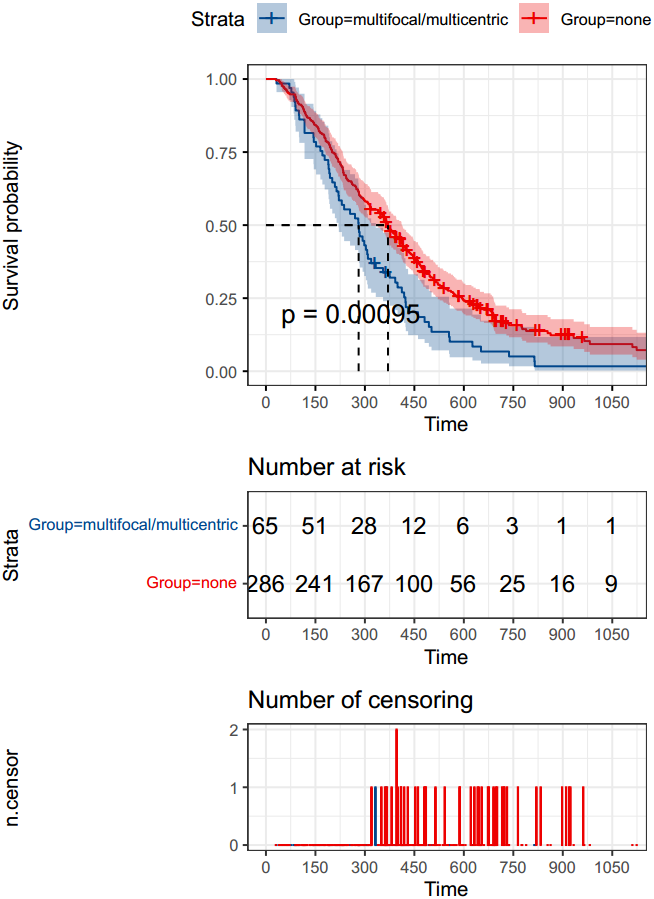

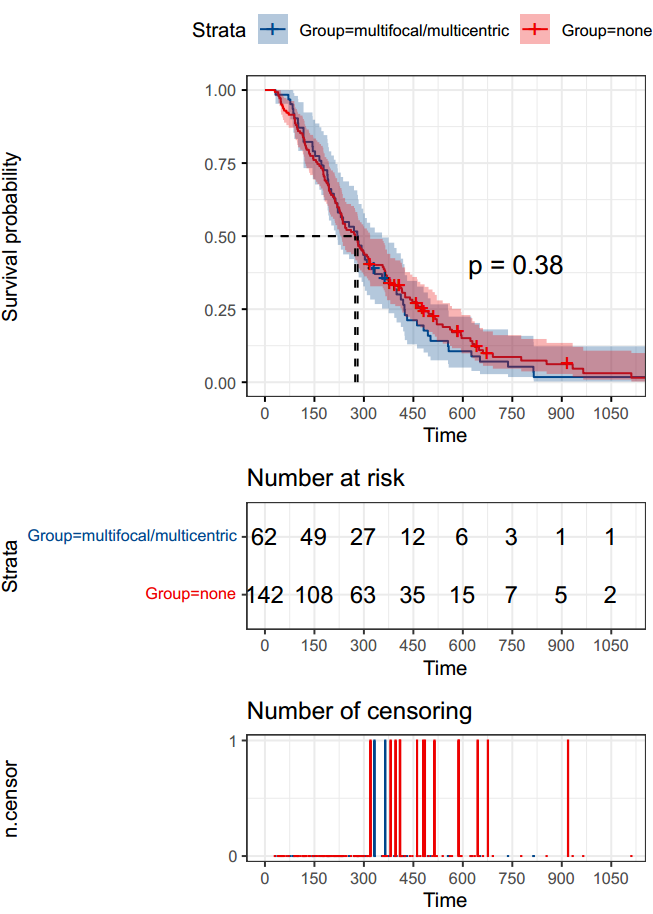


**C**

**B**

**A**

**Figure S8 (A)** love plot: the absolute standard mean differences before matching (red dots) and after matching (green dots). Kaplan-Meier curves for GBM patients stratified by the F9M in both before-matching (**B**) and after-matching (**C**) cohorts.

### Table S18 Sensitivity analysis for F9M after propensity score matching (n=204).

|  | **Univariable** | | | | |  | **Multivariable** | | | | |
| --- | --- | --- | --- | --- | --- | --- | --- | --- | --- | --- | --- |
| **Characteristic** | **N** | **Event N** | **HR** | **95% CI** | ***P*** |  | **N** | **Event N** | **HR** | **95% CI** | ***P*** |
| Age | 204 | 187 | 1.00 | 0.99, 1.01 | 0.501 |  | 204 | 187 | 1.00 | 0.98, 1.01 | 0.408 |
| Gender |  |  |  |  |  |  |  |  |  |  |  |
| female | 79 | 71 | Reference | Reference |  |  | 79 | 71 | Reference | Reference |  |
| male | 125 | 116 | 1.02 | 0.76, 1.38 | 0.883 |  | 125 | 116 | 1.08 | 0.80, 1.46 | 0.623 |
| KPS score | 204 | 187 | 0.94 | 0.92, 0.97 | **<0.001** |  | 204 | 187 | 0.95 | 0.93, 0.97 | **<0.001** |
| EOR |  |  |  |  |  |  |  |  |  |  |  |
| non-GTR | 112 | 106 | Reference | Reference |  |  | 112 | 106 | Reference | Reference |  |
| GTR | 92 | 81 | 0.49 | 0.36, 0.66 | **<0.001** |  | 92 | 81 | 0.42 | 0.30, 0.57 | **<0.001** |
| Therapy |  |  |  |  |  |  |  |  |  |  |  |
| non-SOC | 103 | 101 | Reference | Reference |  |  | 103 | 101 | Reference | Reference |  |
| SOC | 101 | 86 | 0.36 | 0.26, 0.48 | **<0.001** |  | 101 | 86 | 0.32 | 0.24, 0.44 | **<0.001** |
| MGMT_Status |  |  |  |  |  |  |  |  |  |  |  |
| unmethylation | 102 | 98 | Reference | Reference |  |  | 102 | 98 | Reference | Reference |  |
| methylation | 102 | 89 | 0.63 | 0.47, 0.84 | **0.002** |  | 102 | 89 | 0.70 | 0.52, 0.96 | **0.027** |
| F9M |  |  |  |  |  |  |  |  |  |  |  |
| none | 142 | 128 | Reference | Reference |  |  | 142 | 128 | Reference | Reference |  |
| multifocal/multicentric | 62 | 59 | 1.11 | 0.81, 1.51 | 0.514 |  | 62 | 59 | 0.86 | 0.63, 1.19 | 0.372 |

HR, hazard ratio; CI, confidence interval; KPS, Karnofsky performance status; EOR, extent of resection; GTR, gross total resection; SOC, standard of care; MGMT, O^6^-methylguanine DNA methyltransferase.

The bold values represent *P* < 0.05.

## F16_Hemorrhage (yes vs. no)

### Table S19 Baseline characteristics of the study population by F16 before and after propensity score matching.

| **Variables** | **Level** |  | **Before Matching** | | | |  |  | **After Matching^✝^** | | | |
| --- | --- | --- | --- | --- | --- | --- | --- | --- | --- | --- | --- | --- |
|  |  | **no** | | **yes** | ***P*** | **SMD** |  | **no** | | **yes** | ***P*** | **SMD** |
| n |  | 227 | | 124 |  |  |  | 109 | | 109 |  |  |
| Age | median (IQR) | 59 (55-67) | | 55 (46-62) | **<0.001** | -0.460 |  | 58 (51-64) | | 57 (50-63) | 0.611 | -0.021 |
| Gender (%) | male | 138 (60.8) | | 90 (72.6) | **0.036** | 0.264 |  | 78 (71.6) | | 78 (71.6) | 1.000 | 0.000 |
|  | female | 89 (39.2) | | 34 (27.4) |  | -0.264 |  | 31 (28.4) | | 31 (28.4) |  | 0.000 |
| KPS score | median (IQR) | 90 (80-90) | | 90 (80-90) | 0.460 | 0.066 |  | 87.43 (5.68) | | 86.97 (6.16) | 0.491 | -0.073 |
| EOR (%) | GTR | 144 (63.4) | | 87 (70.2) | 0.249 | 0.147 |  | 77 (70.6) | | 76 (69.7) | 1.000 | -0.020 |
|  | non-GTR | 83 (36.6) | | 37 (29.8) |  | -0.147 |  | 32 (29.4) | | 33 (30.3) |  | 0.020 |
| Therapy (%) | SOC | 134 (59.0) | | 70 (56.5) | 0.723 | -0.052 |  | 61 (56.0) | | 61 (56.0) | 1.000 | 0.000 |
|  | non-SOC | 93 (41.0) | | 54 (43.5) |  | 0.052 |  | 48 (44.0) | | 48 (44.0) |  | 0.000 |
| MGMT_Status (%) | methylation | 130 (57.3) | | 62 (50.0) | 0.232 | -0.145 |  | 58 (53.2) | | 57 (52.3) | 1.000 | -0.018 |
|  | unmethylation | 97 (42.7) | | 62 (50.0) |  | 0.145 |  | 51 (46.8) | | 52 (47.7) |  | 0.018 |

SMD, standardized mean difference; IQR, interquartile range; KPS, Karnofsky performance status; EOR, extent of resection; GTR, gross total resection; SOC, standard of care; MGMT, O^6^-methylguanine DNA methyltransferase.

✝Propensity score mathing was calculated using a 1:1 ratio-logistic regression with a nearest-neighbour caliper width of 0.1, matching the following variables: age, gender, KPS, therapy, EOR, MGMT_status.

The bold values represent *P* < 0.05.

### Figure S9


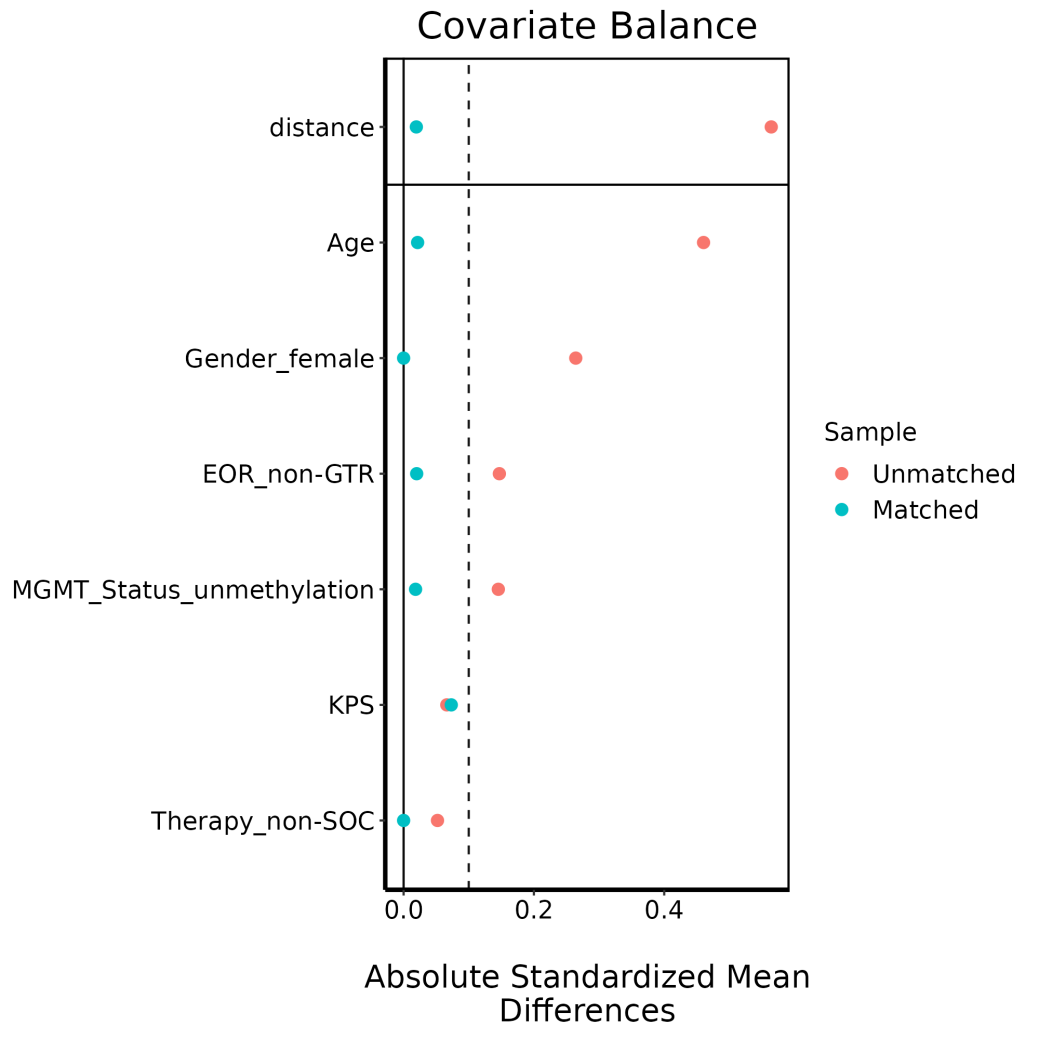

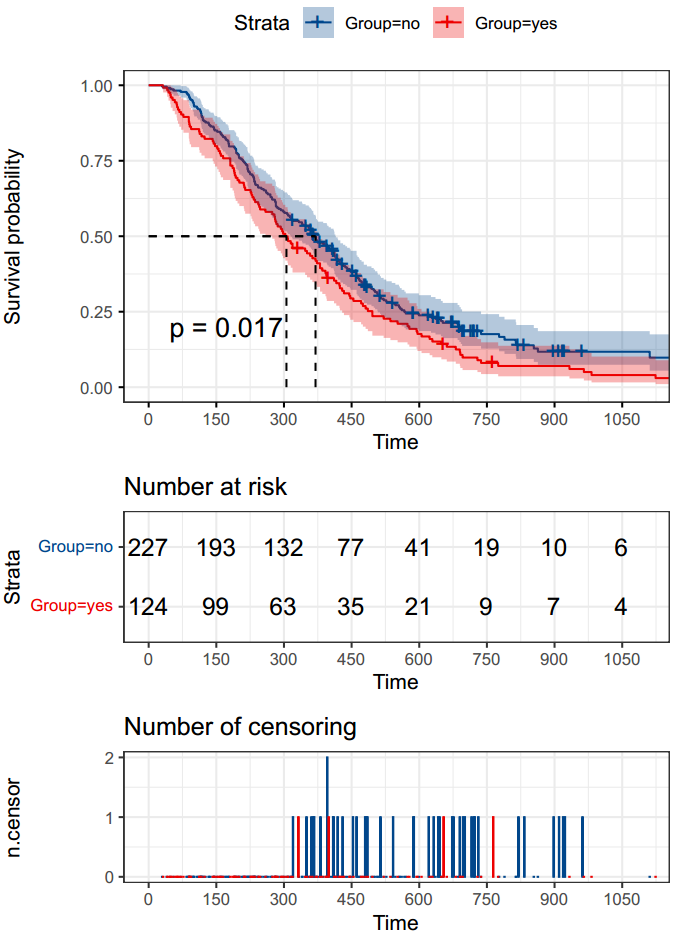

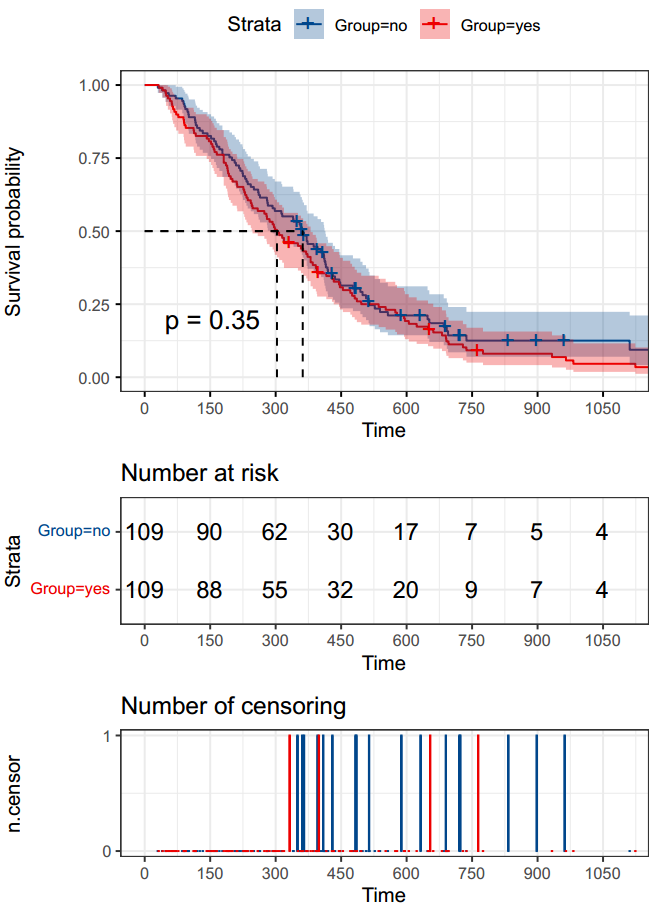


**C**

**A**

**B**

**Figure S9 (A)** love plot: the absolute standard mean differences before matching (red dots) and after matching (green dots). Kaplan-Meier curves for GBM patients stratified by the F16 in both before-matching (**B**) and after-matching (**C**) cohorts.

### Table S20 Sensitivity analysis for F16 after propensity score matching (n=218).

|  | **Univariable** | | | | |  | **Multivariable** | | | | |
| --- | --- | --- | --- | --- | --- | --- | --- | --- | --- | --- | --- |
| **Characteristic** | **N** | **Event N** | **HR** | **95% CI** | ***P*** |  | **N** | **Event N** | **HR** | **95% CI** | ***P*** |
| Age | 218 | 193 | 1.01 | 1.00, 1.02 | 0.140 |  | 218 | 193 | 1.01 | 0.99, 1.02 | 0.424 |
| Gender |  |  |  |  |  |  |  |  |  |  |  |
| male | 156 | 137 | Reference | Reference |  |  | 156 | 137 | Reference | Reference |  |
| female | 62 | 56 | 1.16 | 0.85, 1.58 | 0.357 |  | 62 | 56 | 0.99 | 0.72, 1.36 | 0.940 |
| KPS score | 218 | 193 | 0.96 | 0.94, 0.98 | **0.001** |  | 218 | 193 | 0.96 | 0.94, 0.99 | **0.004** |
| EOR |  |  |  |  |  |  |  |  |  |  |  |
| GTR | 153 | 131 | Reference | Reference |  |  | 153 | 131 | Reference | Reference |  |
| non-GTR | 65 | 62 | 2.54 | 1.86, 3.48 | **<0.001** |  | 65 | 62 | 2.58 | 1.85, 3.59 | **<0.001** |
| Therapy |  |  |  |  |  |  |  |  |  |  |  |
| SOC | 122 | 99 | Reference | Reference |  |  | 122 | 99 | Reference | Reference |  |
| non-SOC | 96 | 94 | 2.65 | 1.98, 3.54 | **<0.001** |  | 96 | 94 | 2.46 | 1.82, 3.32 | **<0.001** |
| MGMT_Status |  |  |  |  |  |  |  |  |  |  |  |
| methylation | 115 | 95 | Reference | Reference |  |  | 115 | 95 | Reference | Reference |  |
| unmethylation | 103 | 98 | 1.47 | 1.11, 1.95 | **0.008** |  | 103 | 98 | 1.46 | 1.09, 1.94 | **0.011** |
| F16 |  |  |  |  |  |  |  |  |  |  |  |
| no | 109 | 91 | Reference | Reference |  |  | 109 | 91 | Reference | Reference |  |
| yes | 109 | 102 | 1.15 | 0.86, 1.52 | 0.346 |  | 109 | 102 | 1.06 | 0.79, 1.41 | 0.704 |

HR, hazard ratio; CI, confidence interval; KPS, Karnofsky performance status; EOR, extent of resection; GTR, gross total resection; SOC, standard of care; MGMT, O^6^-methylguanine DNA methyltransferase.

The bold values represent *P* < 0.05.

## F22M_nCET tumor Crosses Midline (yes vs. no)

### Table S21 Baseline characteristics of the study population by F22M before and after propensity score matching.

| **Variables** | **Level** |  | **Before Matching** | | | |  |  | **After Matching^✝^** | | | |
| --- | --- | --- | --- | --- | --- | --- | --- | --- | --- | --- | --- | --- |
|  |  | **no** | | **yes** | ***P*** | **SMD** |  | **no** | | **yes** | ***P*** | **SMD** |
| n |  | 281 | | 70 |  |  |  | 150 | | 66 |  |  |
| Age | median (IQR) | 59 (52-66) | | 56 (43-63) | **0.007** | -0.369 |  | 57 (48-61) | | 57 (46-63) | 0.903 | 0.010 |
| Gender (%) | male | 184 (65.5) | | 44 (62.9) | 0.786 | -0.054 |  | 99 (66.0) | | 43 (65.2) | 1.000 | -0.000 |
|  | female | 97 (34.5) | | 26 (37.1) |  | 0.054 |  | 51 (34.0) | | 23 (34.8) |  | 0.000 |
| KPS score | median (IQR) | 90 (90-90) | | 90 (80-90) | **<0.001** | -0.380 |  | 90 (80-90) | | 90 (80-90) | 0.147 | -0.041 |
| EOR (%) | GTR | 201 (71.5) | | 30 (42.9) | **<0.001** | -0.579 |  | 85 (56.7) | | 30 (45.5) | 0.170 | 0.005 |
|  | non-GTR | 80 (28.5) | | 40 (57.1) |  | 0.579 |  | 65 (43.3) | | 36 (54.5) |  | -0.005 |
| Therapy (%) | SOC | 175 (62.3) | | 29 (41.4) | **0.002** | -0.423 |  | 73 (48.7) | | 28 (42.4) | 0.485 | -0.041 |
|  | non-SOC | 106 (37.7) | | 41 (58.6) |  | 0.423 |  | 77 (51.3) | | 38 (57.6) |  | 0.041 |
| MGMT_Status (%) | methylation | 158 (56.2) | | 34 (48.6) | 0.309 | -0.153 |  | 82 (54.7) | | 33 (50.0) | 0.628 | -0.071 |
|  | unmethylation | 123 (43.8) | | 36 (51.4) |  | 0.153 |  | 68 (45.3) | | 33 (50.0) |  | 0.071 |

SMD, standardized mean difference; IQR, interquartile range; KPS, Karnofsky performance status; EOR, extent of resection; GTR, gross total resection; SOC, standard of care; MGMT, O^6^-methylguanine DNA methyltransferase.

✝Propensity score mathing was calculated using a 1:3 ratio-logistic regression with a nearest-neighbour caliper width of 0.1, matching the following variables: age, gender, KPS, therapy, EOR, MGMT_status.

The bold values represent *P* < 0.05.

### Figure S10


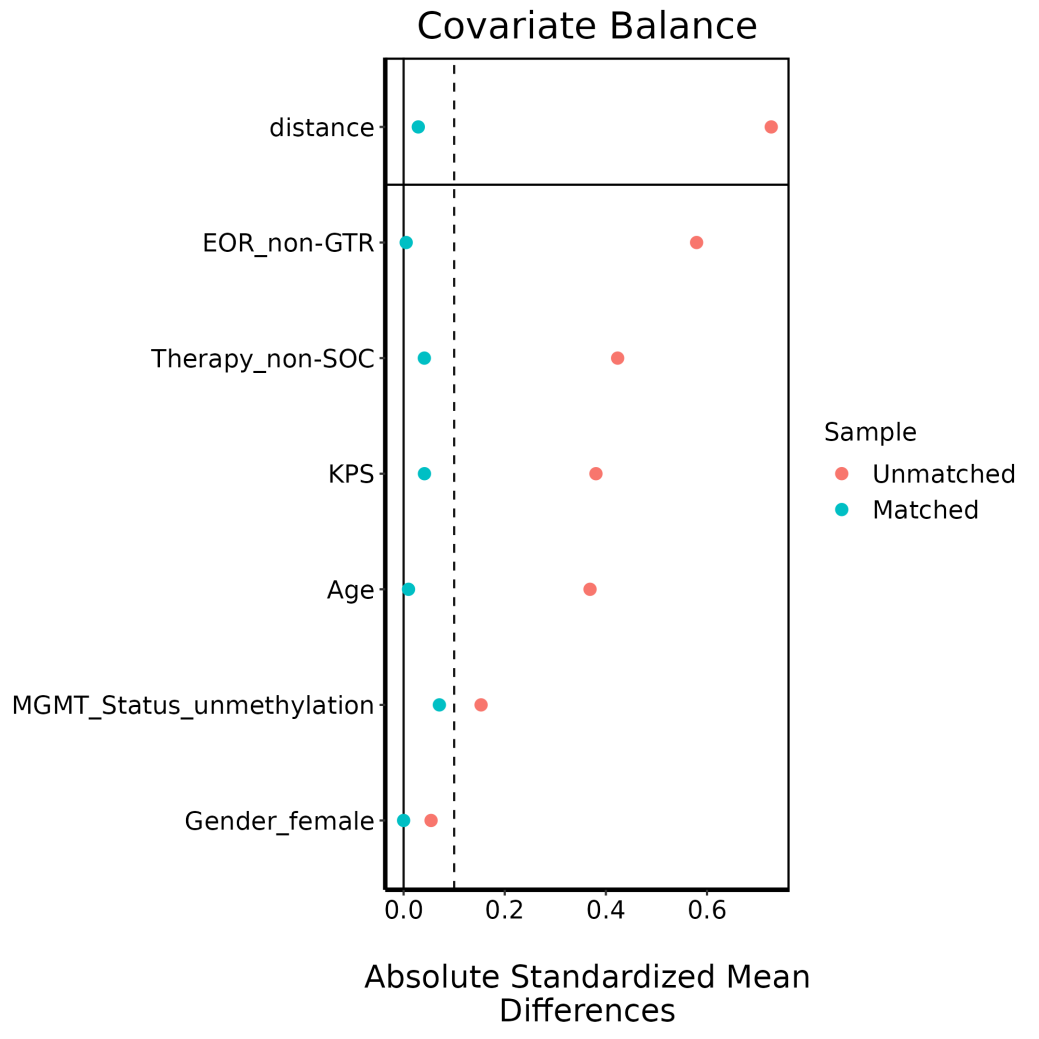

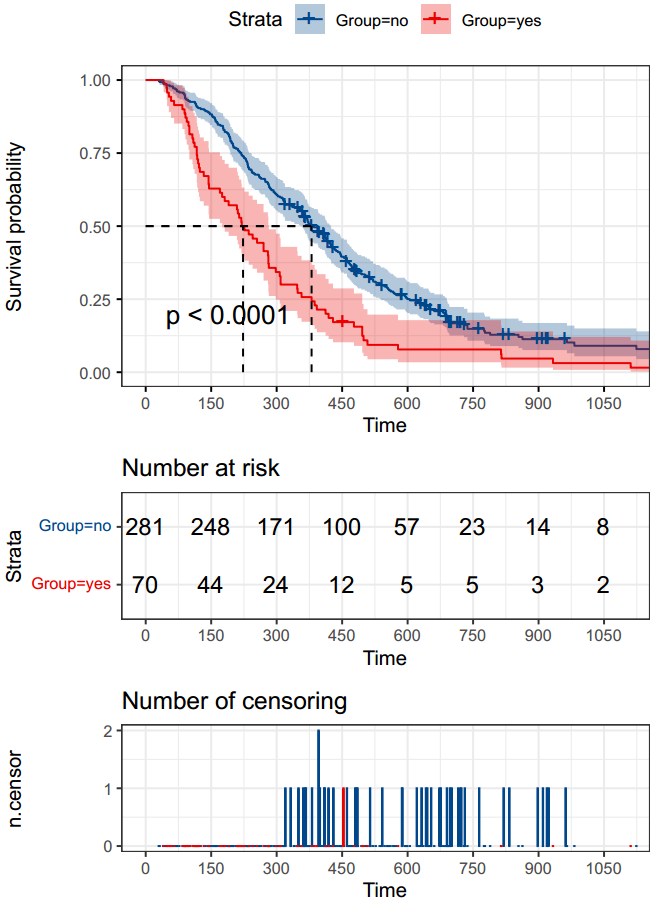

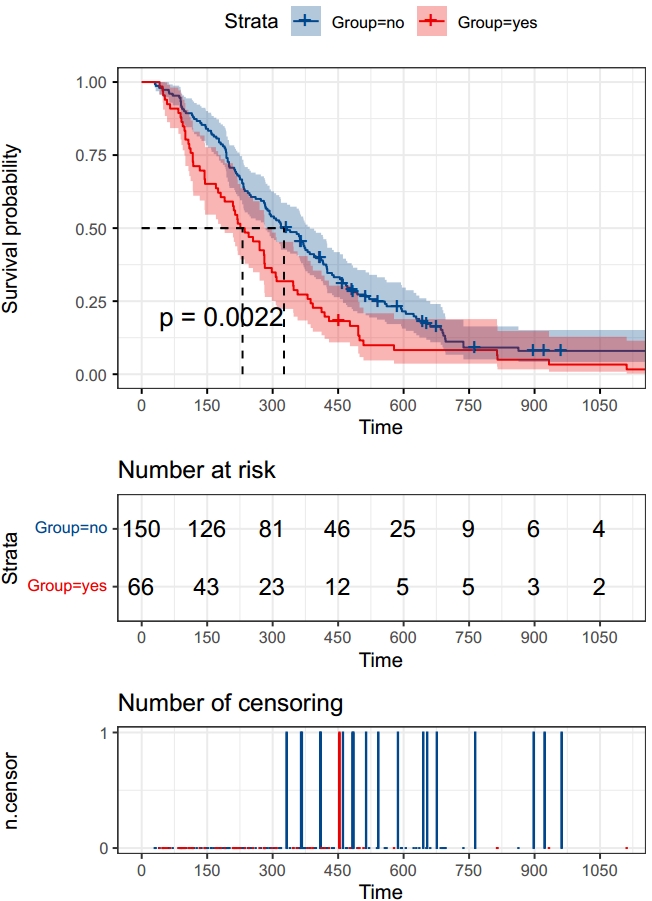


**C**

**A**

**B**

**Figure S10 (A)** love plot: the absolute standard mean differences before matching (red dots) and after matching (green dots). Kaplan-Meier curves for GBM patients stratified by the F22M in both before-matching (**B**) and after-matching (**C**) cohorts.

### Table S22 Sensitivity analysis for F22M after propensity score matching (n=216).

|  | **Univariable** | | | | |  | **Multivariable** | | | | |
| --- | --- | --- | --- | --- | --- | --- | --- | --- | --- | --- | --- |
| **Characteristic** | **N** | **Event N** | **HR** | **95% CI** | ***P*** |  | **N** | **Event N** | **HR** | **95% CI** | ***P*** |
| Age | 216 | 195 | 1.01 | 0.99, 1.02 | 0.328 |  | 216 | 195 | 0.99 | 0.98, 1.00 | 0.213 |
| Gender |  |  |  |  |  |  |  |  |  |  |  |
| male | 142 | 131 | Reference | Reference |  |  | 142 | 131 | Reference | Reference |  |
| female | 74 | 64 | 1.04 | 0.77, 1.40 | 0.819 |  | 74 | 64 | 0.93 | 0.68, 1.27 | 0.654 |
| KPS score | 216 | 195 | 0.95 | 0.93, 0.97 | **<0.001** |  | 216 | 195 | 0.96 | 0.94, 0.99 | **0.003** |
| EOR |  |  |  |  |  |  |  |  |  |  |  |
| GTR | 115 | 99 | Reference | Reference |  |  | 115 | 99 | Reference | Reference |  |
| non-GTR | 101 | 96 | 2.02 | 1.52, 2.70 | **<0.001** |  | 101 | 96 | 2.42 | 1.76, 3.33 | **<0.001** |
| Therapy |  |  |  |  |  |  |  |  |  |  |  |
| SOC | 101 | 84 | Reference | Reference |  |  | 101 | 84 | Reference | Reference |  |
| non-SOC | 115 | 111 | 2.16 | 1.62, 2.88 | **<0.001** |  | 115 | 111 | 2.73 | 1.98, 3.75 | **<0.001** |
| MGMT_Status |  |  |  |  |  |  |  |  |  |  |  |
| methylation | 115 | 97 | Reference | Reference |  |  | 115 | 97 | Reference | Reference |  |
| unmethylation | 101 | 98 | 1.77 | 1.33, 2.35 | **<0.001** |  | 101 | 98 | 1.47 | 1.09, 1.98 | **0.011** |
| F22M |  |  |  |  |  |  |  |  |  |  |  |
| no | 150 | 130 | Reference | Reference |  |  | 150 | 130 | Reference | Reference |  |
| yes | 66 | 65 | 1.56 | 1.16, 2.10 | **0.004** |  | 66 | 65 | 0.89 | 0.89, 1.67 | 0.218 |

HR, hazard ratio; CI, confidence interval; KPS, Karnofsky performance status; EOR, extent of resection; GTR, gross total resection; SOC, standard of care; MGMT, O^6^-methylguanine DNA methyltransferase.

The bold values represent *P* < 0.05.

## F24_Satellites (yes vs. no)

### Table S23 Baseline characteristics of the study population by F24 before and after propensity score matching.

| **Variables** | **Level** |  | **Before Matching** | | | |  |  | **After Matching^✝^** | | | |
| --- | --- | --- | --- | --- | --- | --- | --- | --- | --- | --- | --- | --- |
|  |  | **no** | | **yes** | ***P*** | **SMD** |  | **no** | | **yes** | ***P*** | **SMD** |
| n |  | 320 | | 31 |  |  |  | 109 | | 31 |  |  |
| Age | median (IQR) | 58 (51-65) | | 62 (57-67) | 0.069 | 0.394 |  | 59 (55-66) | | 62 (57-67) | 0.340 | 0.175 |
| Gender (%) | male | 209 (65.3) | | 19 (61.3) | 0.802 | -0.083 |  | 73 (67.0) | | 19 (61.3) | 0.709 | -0.072 |
|  | female | 111 (34.7) | | 12 (38.7) |  | 0.083 |  | 36 (33.0) | | 12 (38.7) |  | 0.072 |
| KPS score | median (IQR) | 90 (80-90) | | 90 (80-90) | 0.240 | -0.204 |  | 90 (80-90) | | 90 (80-90) | 0.986 | 0.020 |
| EOR (%) | GTR | 219 (68.4) | | 12 (38.7) | **0.002** | -0.610 |  | 43 (39.4) | | 12 (38.7) | 1.000 | 0.083 |
|  | non-GTR | 101 (31.6) | | 19 (61.3) |  | 0.610 |  | 66 (60.6) | | 19 (61.3) |  | -0.083 |
| Therapy (%) | SOC | 191 (59.7) | | 13 (41.9) | 0.085 | -0.360 |  | 42 (38.5) | | 13 (41.9) | 0.893 | 0.049 |
|  | non-SOC | 129 (40.3) | | 18 (58.1) |  | 0.360 |  | 67 (61.5) | | 18 (58.1) |  | -0.049 |
| MGMT_Status (%) | methylation | 181 (56.6) | | 11 (35.5) | **0.039** | -0.441 |  | 46 (42.2) | | 11 (35.5) | 0.642 | -0.056 |
|  | unmethylation | 139 (43.4) | | 20 (64.5) |  | 0.441 |  | 63 (57.8) | | 20 (64.5) |  | 0.056 |

SMD, standardized mean difference; IQR, interquartile range; KPS, Karnofsky performance status; EOR, extent of resection; GTR, gross total resection; SOC, standard of care; MGMT, O^6^-methylguanine DNA methyltransferase.

✝Propensity score mathing was calculated using a 1:4 ratio-logistic regression with a nearest-neighbour caliper width of 0.1, matching the following variables: age, gender, KPS, therapy, EOR, MGMT status.

The bold values represent *P* < 0.05.

### Figure S11


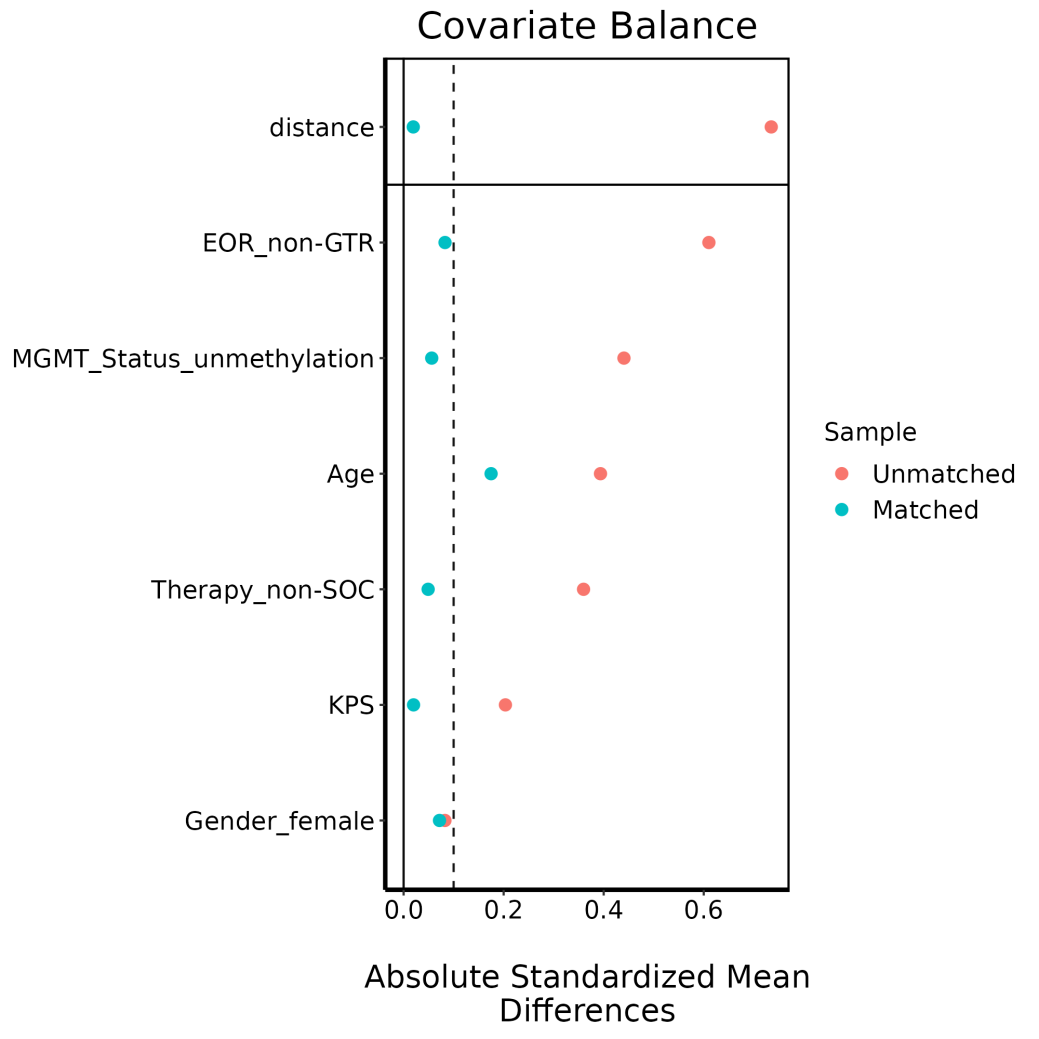

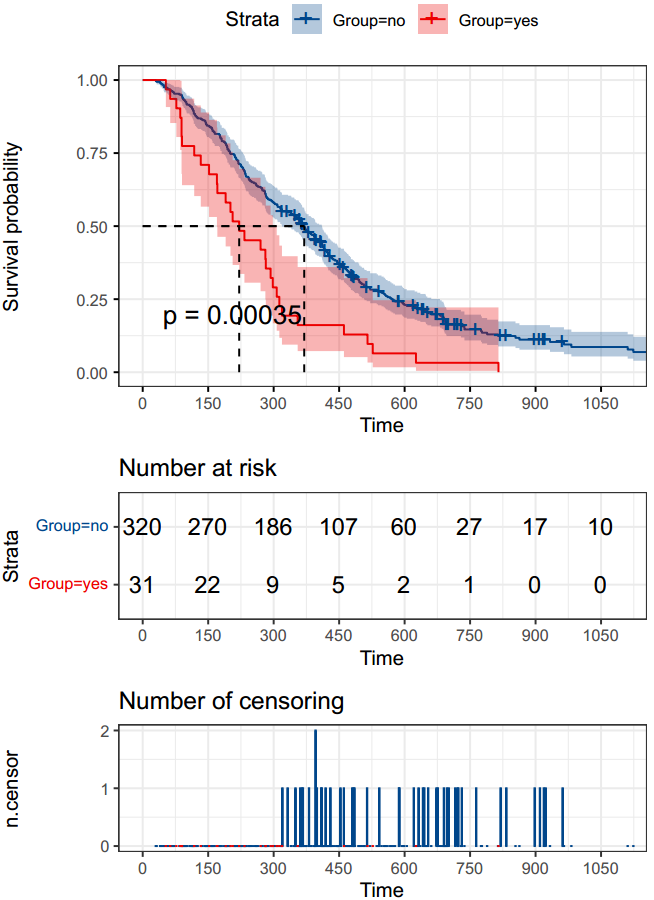

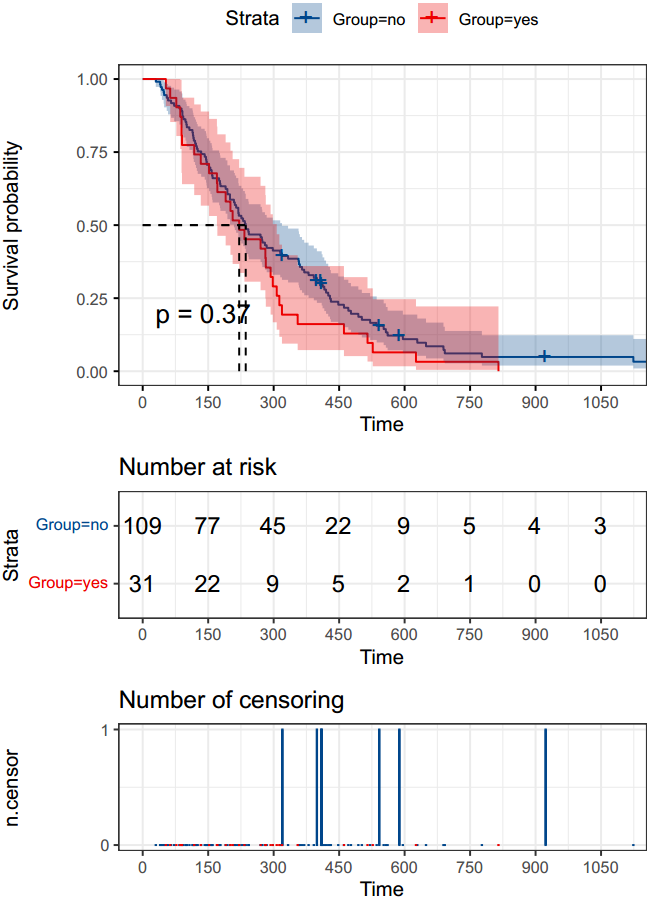


**A**

**C**

**B**

**Figure S11 (A)** love plot: the absolute standard mean differences before matching (red dots) and after matching (green dots). Kaplan-Meier curves for GBM patients stratified by the F24 in both before-matching (**B**) and after-matching (**C**) cohorts.

### Table S24 Sensitivity analysis for F24 after propensity score matching (n=140).

|  | **Univariable** | | | | |  | **Multivariable** | | | | |
| --- | --- | --- | --- | --- | --- | --- | --- | --- | --- | --- | --- |
| **Characteristic** | **N** | **Event N** | **HR** | **95% CI** | ***P*** |  | **N** | **Event N** | **HR** | **95% CI** | ***P*** |
| Age | 140 | 132 | 1.01 | 0.99, 1.03 | 0.249 |  | 140 | 132 | 1.01 | 0.99, 1.03 | 0.322 |
| Gender |  |  |  |  |  |  |  |  |  |  |  |
| female | 48 | 43 | Reference | Reference |  |  | 48 | 43 | Reference | Reference |  |
| male | 92 | 89 | 1.16 | 0.80, 1.67 | 0.444 |  | 92 | 89 | 1.25 | 0.85, 1.82 | 0.253 |
| KPS score | 140 | 132 | 0.96 | 0.94, 0.98 | **<0.001** |  | 140 | 132 | 0.97 | 0.94, 0.99 | **0.009** |
| EOR |  |  |  |  |  |  |  |  |  |  |  |
| non-GTR | 85 | 81 | Reference | Reference |  |  | 85 | 81 | Reference | Reference |  |
| GTR | 55 | 51 | 0.49 | 0.34, 0.70 | **<0.001** |  | 55 | 51 | 0.36 | 0.24, 0.54 | **<0.001** |
| Therapy |  |  |  |  |  |  |  |  |  |  |  |
| non-SOC | 85 | 83 | Reference | Reference |  |  | 85 | 83 | Reference | Reference |  |
| SOC | 55 | 49 | 0.41 | 0.29, 0.59 | **<0.001** |  | 55 | 49 | 0.35 | 0.24, 0.51 | **<0.001** |
| MGMT_Status |  |  |  |  |  |  |  |  |  |  |  |
| unmethylation | 83 | 81 | Reference | Reference |  |  | 83 | 81 | Reference | Reference |  |
| methylation | 57 | 51 | 0.87 | 0.61, 1.24 | 0.439 |  | 57 | 51 | 0.65 | 0.45, 0.95 | **0.025** |
| F24 |  |  |  |  |  |  |  |  |  |  |  |
| no | 109 | 101 | Reference | Reference |  |  | 109 | 101 | Reference | Reference |  |
| yes | 31 | 31 | 1.34 | 0.89, 2.00 | 0.161 |  | 31 | 31 | 1.18 | 0.76, 1.82 | 0.468 |

HR, hazard ratio; CI, confidence interval; KPS, Karnofsky performance status; EOR, extent of resection; GTR, gross total resection; SOC, standard of care; MGMT, O^6^-methylguanine DNA methyltransferase.

The bold values represent *P* < 0.05.

# Clinical model construction

### Table S25 Uni- and multivariate Cox regression analyses of demographic, clinical, and pathological parameters.

| **Characteristic** | **Univariable** | | | | |  | **Multivariable** | | | | |
| --- | --- | --- | --- | --- | --- | --- | --- | --- | --- | --- | --- |
|  | **N** | **Event N** | **HR** | **95% CI** | ***P*** |  | **N** | **Event N** | **HR** | **95% CI** | ***P*** |
| Age | 211 | 182 | 1.00 | 0.99, 1.01 | 0.928 |  | 211 | 182 | 1.00 | 0.99, 1.01 | 0.772 |
| Gender |  |  |  |  |  |  |  |  |  |  |  |
| male | 143 | 127 | Reference | Reference |  |  | 143 | 127 | Reference | Reference |  |
| female | 68 | 55 | 0.77 | 0.56, 1.06 | 0.114 |  | 68 | 55 | 0.69 | 0.50, 0.95 | **0.022** |
| KPS score | 211 | 182 | 0.95 | 0.93, 0.97 | **<0.001** |  | 211 | 182 | 0.97 | 0.94, 0.99 | **0.011** |
| EOR |  |  |  |  |  |  |  |  |  |  |  |
| GTR | 134 | 111 | Reference | Reference |  |  | 134 | 111 | Reference | Reference |  |
| non-GTR | 77 | 71 | 2.08 | 1.53, 2.82 | **<0.001** |  | 77 | 71 | 2.28 | 1.65, 3.15 | **<0.001** |
| Therapy |  |  |  |  |  |  |  |  |  |  |  |
| SOC | 120 | 95 | Reference | Reference |  |  | 120 | 95 | Reference | Reference |  |
| non-SOC | 91 | 87 | 2.47 | 1.83, 3.32 | **<0.001** |  | 91 | 87 | 2.70 | 1.99, 3.67 | **<0.001** |
| MGMT_Status |  |  |  |  |  |  |  |  |  |  |  |
| methylation | 119 | 94 | Reference | Reference |  |  | 119 | 94 | Reference | Reference |  |
| unmethylation | 92 | 88 | 1.80 | 1.34, 2.42 | **<0.001** |  | 92 | 88 | 1.83 | 1.35, 2.47 | **<0.001** |

HR, hazard ratio; CI, confidence interval; KPS, Karnofsky performance status; EOR, extent of resection; GTR, gross total resection; SOC, standard of care; MGMT, O^6^-methylguanine DNA methyltransferase.

The bold values represent *P* < 0.05.

Note: Clinical model was constructed based on age, gender, KPS, EOR, therapy, and MGMT_status.

# Combined model construction

### Table S26 Uni- and multivariate Cox regression analyses of demographic, clinical, pathological, and VASARI parameters.

| **Characteristic** | **Univariable** | | | | | |  | **Multivariable** | | | | | |
| --- | --- | --- | --- | --- | --- | --- | --- | --- | --- | --- | --- | --- | --- |
|  | **N** | **Event N** | **HR**^1^ | **95% CI**^1^ | ***P*** |  | | | **N** | **Event N** | **HR**^1^ | **95% CI**^1^ | ***P*** |
| Age | 211 | 182 | 1.00 | 0.99, 1.01 | 0.928 |  | | | 211 | 182 | 1.01 | 0.99, 1.02 | 0.241 |
| Gender |  |  |  |  |  |  | | |  |  |  |  |  |
| male | 143 | 127 | Reference | Reference |  |  | | | 143 | 127 | Reference | Reference |  |
| female | 68 | 55 | 0.77 | 0.56, 1.06 | 0.114 |  | | | 68 | 55 | 0.76 | 0.54, 1.05 | 0.099 |
| KPS score | 211 | 182 | 0.95 | 0.93, 0.97 | **<0.001** |  | | | 211 | 182 | 0.97 | 0.95, 1.00 | **0.040** |
| EOR |  |  |  |  |  |  | | |  |  |  |  |  |
| GTR | 134 | 111 | Reference | Reference |  |  | | | 134 | 111 | Reference | Reference |  |
| non-GTR | 77 | 71 | 2.08 | 1.53, 2.82 | **<0.001** |  | | | 77 | 71 | 1.78 | 1.26, 2.51 | **0.001** |
| Therapy |  |  |  |  |  |  | | |  |  |  |  |  |
| SOC | 120 | 95 | Reference | Reference |  |  | | | 120 | 95 | Reference | Reference |  |
| non-SOC | 91 | 87 | 2.47 | 1.83, 3.32 | **<0.001** |  | | | 91 | 87 | 2.77 | 2.02, 3.80 | **<0.001** |
| MGMT_Status |  |  |  |  |  |  | | |  |  |  |  |  |
| methylation | 119 | 94 | Reference | Reference |  |  | | | 119 | 94 | Reference | Reference |  |
| unmethylation | 92 | 88 | 1.80 | 1.34, 2.42 | **<0.001** |  | | | 92 | 88 | 1.73 | 1.25, 2.38 | **<0.001** |
| F2M (Side of Tumor Epicenter) |  |  |  |  |  |  | | |  |  |  |  |  |
| unilateral | 183 | 154 | Reference | Reference |  |  | | | 183 | 154 | Reference | Reference |  |
| center/bilateral | 28 | 28 | 2.14 | 1.43, 3.22 | **<0.001** |  | | | 28 | 28 | 0.70 | 0.39, 1.26 | 0.232 |
| F12M (Definition of the enhancing margin) |  |  |  |  |  |  | | |  |  |  |  |  |
| well-defined | 66 | 50 | Reference | Reference |  |  | | | 66 | 50 | Reference | Reference |  |
| poorly-defined | 145 | 132 | 1.82 | 1.31, 2.52 | **<0.001** |  | | | 145 | 132 | 1.17 | 0.81, 1.69 | 0.400 |
| F15M (Edema Crosses Midline) |  |  |  |  |  |  | | |  |  |  |  |  |
| no | 185 | 156 | Reference | Reference |  |  | | | 185 | 156 | Reference | Reference |  |
| yes | 26 | 26 | 2.44 | 1.60, 3.70 | **<0.001** |  | | | 26 | 26 | 1.75 | 1.05, 2.90 | **0.031** |
| F21 (Deep WM invasion) |  |  |  |  |  |  | | |  |  |  |  |  |
| no | 169 | 142 | Reference | Reference |  |  | | | 169 | 142 | Reference | Reference |  |
| yes | 42 | 40 | 2.12 | 1.49, 3.03 | **<0.001** |  | | | 42 | 40 | 1.45 | 1.04, 1.86 | **0.007** |
| F23M (Enhancing tumor Crosses Midline) |  |  |  |  |  |  | | |  |  |  |  |  |
| no | 185 | 156 | Reference | Reference |  |  | | | 185 | 156 | Reference | Reference |  |
| yes | 26 | 26 | 2.46 | 1.61, 3.76 | **<0.001** |  | | | 26 | 26 | 2.09 | 1.30, 3.36 | **0.002** |
| F19 (Ependymal invasion) |  |  |  |  |  |  | | |  |  |  |  |  |
| no | 84 | 59 | Reference | Reference |  |  | | | 84 | 59 | Reference | Reference |  |
| yes | 127 | 123 | 2.62 | 1.91, 3.59 | **<0.001** |  | | | 127 | 123 | 1.70 | 1.20, 2.43 | **0.003** |

HR, hazard ratio; CI, confidence interval; KPS, Karnofsky performance status; EOR, extent of resection; GTR, gross total resection; SOC, standard of care; MGMT, O^6^-methylguanine DNA methyltransferase.

The bold values represent *P* < 0.05.

Note: Combined model was constructed based on age, gender, KPS, EOR, therapy, MGMT_status, F21, F15M, F23M, and F19.
